# Supplementary material for: Prenatal opioid exposure alters pain perception and increases long-term health risks in infants with neonatal opioid withdrawal syndrome
Source: Front Pain Res (Lausanne). 2025 Apr 17;6:1497801. doi: 10.3389/fpain.2025.1497801 (PMC12043715; doi:10.3389/fpain.2025.1497801)
Supplement: Supplementary file 5 [file Table1.docx]

**Table-1.** A comprehensive list of 111 CpG targets exhibiting significant methylation differences in NOWS is provided. This includes CpG sites with Target ID, pain-associated gene IDs, chromosome locations, p-values, FDR p-values, and the percentage of methylation difference.

| **TargetID** | **Gene** | **location** | **p-Val** | **FDR p-Val** | **% Methylation** | | | **AUC** | **CI** | |
| --- | --- | --- | --- | --- | --- | --- | --- | --- | --- | --- |
|  |  |  |  |  | **Cases** | **Control** | **Difference** |  | **lower** | **upper** |
| cg26635219 | CFTR | 7q31.2 | 9.67 × 10⁻³⁹ | 8.36 × 10⁻³³ | 24.59 | 14.19 | 10.40 | 0.76 | 0.64 | 0.88 |
| cg13999099 | IL6ST | 5q11.2 | 2.41 × 10⁻³⁸ | 2.08 × 10⁻³² | 26.41 | 16.95 | 9.46 | 0.92 | 0.85 | 0.99 |
| cg22018329 | GNA11 | 19p13.3 | 1.03 × 10⁻³⁷ | 8.91 × 10⁻³² | 85.92 | 78.79 | 7.13 | 0.77 | 0.65 | 0.88 |
| cg03659519 | GALR1 | 18q23 | 5.45 × 10⁻³⁷ | 4.72 × 10⁻³¹ | 17.00 | 10.84 | 6.16 | 0.68 | 0.56 | 0.80 |
| cg15677797 | KLF11 | 2p25.1 | 7.74 × 10⁻²¹ | 6.70 × 10⁻¹⁵ | 6.37 | 13.28 | -6.91 | 0.57 | 0.45 | 0.69 |
| cg02904605 | NOTCH3 | 19p13.12 | 6.01 × 10⁻¹⁷ | 5.20 × 10⁻¹¹ | 64.42 | 74.86 | -10.43 | 0.78 | 0.67 | 0.89 |
| cg21517792 | MTA1 | 14q32.33 | 5.80 × 10⁻¹⁵ | 5.01 × 10⁻⁹ | 78.15 | 85.62 | -7.46 | 0.82 | 0.71 | 0.92 |
| cg14117934 | IL1B | 2q14.1 | 1.07 × 10⁻¹⁴ | 9.26 × 10⁻⁹ | 77.75 | 69.36 | 8.39 | 0.81 | 0.70 | 0.91 |
| cg14929554 | N4BP1 | 16q12.1 | 2.57 × 10⁻¹⁴ | 2.22 × 10⁻⁸ | 60.20 | 70.37 | -10.17 | 0.76 | 0.65 | 0.88 |
| cg13377102 | KCNAB3 | 17p13.1 | 6.63 × 10⁻¹⁴ | 5.73 × 10⁻⁸ | 70.71 | 79.30 | -8.59 | 0.80 | 0.69 | 0.91 |
| cg04708753 | CASP9 | 1p36.21 | 1.07 × 10⁻¹³ | 9.24 × 10⁻⁸ | 5.83 | 11.12 | -5.28 | 0.61 | 0.49 | 0.74 |
| cg15929698 | NPY | 7p15.3 | 1.25 × 10⁻¹³ | 1.08 × 10⁻⁷ | 50.16 | 40.25 | 9.90 | 0.75 | 0.63 | 0.87 |
| cg08929188 | CALCA | 11p15.2 | 2.00 × 10⁻¹³ | 1.73 × 10⁻⁷ | 25.95 | 17.92 | 8.02 | 0.75 | 0.63 | 0.87 |
| cg16839955 | ARNTL | 11p15.3 | 4.03 × 10⁻¹³ | 3.48 × 10⁻⁷ | 28.33 | 20.13 | 8.20 | 0.81 | 0.71 | 0.92 |
| cg15442907 | CACNA1C | 12p13.33 | 5.63 × 10⁻¹³ | 4.87 × 10⁻⁷ | 45.17 | 55.79 | -10.62 | 0.78 | 0.67 | 0.90 |
| cg06976250 | ANKK1 | 11q23.2 | 5.99 × 10⁻¹² | 5.18 × 10⁻⁶ | 16.26 | 10.96 | 5.30 | 0.59 | 0.46 | 0.71 |
| cg22623236 | PDE10A | 6q27 | 9.31 × 10⁻¹² | 8.06 × 10⁻⁶ | 62.35 | 71.33 | -8.98 | 0.72 | 0.60 | 0.85 |
| cg09711113 | NLGN2 | 17p13.1 | 1.11 × 10⁻¹¹ | 9.60 × 10⁻⁶ | 53.95 | 63.63 | -9.69 | 0.75 | 0.62 | 0.87 |
| cg25885356 | RUNX1 | 21q22.12 | 1.19 × 10⁻¹¹ | 1.03 × 10⁻⁵ | 44.32 | 35.28 | 9.04 | 0.72 | 0.59 | 0.85 |
| cg27331241 | PRKAR1B | 7p22.3 | 1.77 × 10⁻¹¹ | 1.54 × 10⁻⁵ | 52.56 | 62.30 | -9.74 | 0.71 | 0.59 | 0.84 |
| cg22197205 | CLIC4 | 1p36.11 | 1.83 × 10⁻¹¹ | 1.58 × 10⁻⁵ | 75.08 | 82.18 | -7.10 | 0.75 | 0.64 | 0.87 |
| cg08253824 | SCN8A | 12q13.13 | 1.85 × 10⁻¹¹ | 1.60 × 10⁻⁵ | 60.21 | 69.29 | -9.09 | 0.71 | 0.59 | 0.84 |
| cg03037030 | TNF | 6p21.33 | 3.01 × 10⁻¹¹ | 2.61 × 10⁻⁵ | 17.77 | 12.39 | 5.38 | 0.73 | 0.62 | 0.85 |
| cg16243402 | OPRM1 | [6q25.2](https://www.omim.org/geneMap/6/964?start=-3&limit=10&highlight=964) | 3.25 × 10⁻¹¹ | 2.81 × 10⁻⁵ | 22.90 | 30.46 | -7.56 | 0.62 | 0.50 | 0.74 |
| cg17546721 | TGFBR2 | 3p24.1 | 5.00 × 10⁻¹¹ | 4.32 × 10⁻⁵ | 53.38 | 44.44 | 8.95 | 0.75 | 0.63 | 0.87 |
| cg03652989 | ULK1 | 12q24.33 | 5.15 × 10⁻¹¹ | 4.45 × 10⁻⁵ | 80.91 | 74.25 | 6.66 | 0.77 | 0.65 | 0.89 |
| cg11978118 | ADORA2A | 22q11.23 | 6.95 × 10⁻¹¹ | 6.01 × 10⁻⁵ | 71.99 | 79.40 | -7.40 | 0.77 | 0.66 | 0.89 |
| cg00248439 | GRK5 | 10q26.11 | 8.53 × 10⁻¹¹ | 7.38 × 10⁻⁵ | 89.72 | 84.80 | 4.93 | 0.70 | 0.57 | 0.83 |
| cg16293347 | TAOK3 | 12q24.23 | 1.15 × 10⁻¹⁰ | 9.95 × 10⁻⁵ | 80.32 | 73.63 | 6.68 | 0.77 | 0.65 | 0.88 |
| cg17782167 | PLCE1 | 10q23.33 | 1.20 × 10⁻¹⁰ | 1.03 × 10⁻⁴ | 63.77 | 55.31 | 8.46 | 0.75 | 0.63 | 0.87 |
| cg15118537 | PRKG1 | 10q11.23-q21.1 | 1.31 × 10⁻¹⁰ | 1.13 × 10⁻⁴ | 72.65 | 64.95 | 7.69 | 0.73 | 0.60 | 0.85 |
| cg09778136 | ATG5 | 6q21 | 1.32 × 10⁻¹⁰ | 1.14 × 10⁻⁴ | 49.51 | 58.94 | -9.42 | 0.75 | 0.63 | 0.87 |
| cg11211173 | ATP1A2 | 1q23.2 | 1.47 × 10⁻¹⁰ | 1.27 × 10⁻⁴ | 75.90 | 68.59 | 7.31 | 0.74 | 0.61 | 0.86 |
| cg24062706 | OSM | 22q12.2 | 1.60 × 10⁻¹⁰ | 1.38 × 10⁻⁴ | 36.26 | 28.16 | 8.10 | 0.71 | 0.59 | 0.84 |
| cg20629735 | IL18R1 | [2q12.1](https://www.omim.org/geneMap/2/512?start=-3&limit=10&highlight=512) | 1.72 × 10⁻¹⁰ | 1.49 × 10⁻⁴ | 73.99 | 80.95 | -6.96 | 0.76 | 0.65 | 0.88 |
| cg24368031 | MRC2 | 17q23.2 | 1.75 × 10⁻¹⁰ | 1.52 × 10⁻⁴ | 28.42 | 20.97 | 7.45 | 0.75 | 0.63 | 0.87 |
| cg12339476 | MAPK10 | 4q21.3 | 2.34 × 10⁻¹⁰ | 2.02 × 10⁻⁴ | 63.21 | 71.53 | -8.32 | 0.73 | 0.60 | 0.85 |
| cg25387779 | GNAO1 | 16q13 | 4.10 × 10⁻¹⁰ | 3.55 × 10⁻⁴ | 18.94 | 26.52 | -7.58 | 0.65 | 0.51 | 0.78 |
| cg01015652 | ESR2 | 14q23.2-q23.3 | 4.44 × 10⁻¹⁰ | 3.84 × 10⁻⁴ | 16.52 | 10.78 | 5.74 | 0.78 | 0.66 | 0.89 |
| cg09031352 | PTN | 7q33 | 5.47 × 10⁻¹⁰ | 4.73 × 10⁻⁴ | 38.19 | 47.35 | -9.16 | 0.75 | 0.63 | 0.87 |
| cg14299235 | ABCA1 | 9q31.1 | 5.51 × 10⁻¹⁰ | 4.77 × 10⁻⁴ | 67.75 | 59.84 | 7.91 | 0.68 | 0.55 | 0.81 |
| cg05313261 | MAPK3 | 16p11.2 | 5.72 × 10⁻¹⁰ | 4.95 × 10⁻⁴ | 6.60 | 11.74 | -5.14 | 0.88 | 0.80 | 0.97 |
| cg03402505 | GRIA1 | 5q33.2 | 5.84 × 10⁻¹⁰ | 5.05 × 10⁻⁴ | 57.48 | 49.10 | 8.38 | 0.78 | 0.67 | 0.90 |
| cg10274696 | C7orf10 | 7p14.1 | 5.86 × 10⁻¹⁰ | 5.07 × 10⁻⁴ | 64.73 | 72.72 | -7.99 | 0.78 | 0.67 | 0.90 |
| cg06942027 | KCNJ2 | 17q24.3 | 6.04 × 10⁻¹⁰ | 5.23 × 10⁻⁴ | 20.18 | 14.00 | 6.19 | 0.70 | 0.57 | 0.83 |
| cg19690051 | CAPN1 | 11q13.1 | 8.10 × 10⁻¹⁰ | 7.00 × 10⁻⁴ | 15.48 | 9.98 | 5.51 | 0.89 | 0.80 | 0.97 |
| cg12578250 | PRDM16 | 1p36.32 | 1.10 × 10⁻⁹ | 9.50 × 10⁻⁴ | 58.24 | 66.72 | -8.48 | 0.73 | 0.60 | 0.85 |
| cg18302652 | CXCL8 | 4q13.3 | 1.24 × 10⁻⁹ | 1.07 × 10⁻³ | 15.36 | 21.35 | -5.99 | 0.65 | 0.53 | 0.77 |
| cg02081889 | PCSK5 | [9q21.13](https://www.omim.org/geneMap/9/246?start=-3&limit=10&highlight=246) | 1.32 × 10⁻⁹ | 1.14 × 10⁻³ | 14.37 | 9.15 | 5.22 | 0.79 | 0.68 | 0.90 |
| cg07616332 | SHMT1 | 17p11.2 | 1.35 × 10⁻⁹ | 1.17 × 10⁻³ | 63.11 | 55.05 | 8.06 | 0.71 | 0.59 | 0.84 |
| cg12602112 | EDNRB | 13q22.3 | 1.41 × 10⁻⁹ | 1.22 × 10⁻³ | 11.09 | 17.06 | -5.97 | 0.70 | 0.58 | 0.83 |
| cg15787454 | CPQ | 8q22.1 | 1.53 × 10⁻⁹ | 1.33 × 10⁻³ | 76.04 | 82.37 | -6.32 | 0.74 | 0.62 | 0.86 |
| cg16910896 | LPAR1 | 9q31.3 | 1.55 × 10⁻⁹ | 1.34 × 10⁻³ | 64.85 | 72.65 | -7.80 | 0.72 | 0.59 | 0.84 |
| cg12056044 | IL23R | 1p31.3 | 1.78 × 10⁻⁹ | 1.54 × 10⁻³ | 60.52 | 68.71 | -8.20 | 0.75 | 0.63 | 0.87 |
| cg20583095 | ESR1 | 6q25.1-q25.2 | 1.84 × 10⁻⁹ | 1.59 × 10⁻³ | 34.11 | 26.51 | 7.61 | 0.77 | 0.65 | 0.88 |
| cg13480658 | AJAP1 | 1p36.32 | 2.05 × 10⁻⁹ | 1.77 × 10⁻³ | 89.37 | 93.23 | -3.87 | 0.72 | 0.59 | 0.84 |
| cg04329125 | CRIP2 | [14q32.33](https://www.omim.org/geneMap/14/596?start=-3&limit=10&highlight=596) | 2.05 × 10⁻⁹ | 1.77 × 10⁻³ | 47.33 | 39.10 | 8.23 | 0.70 | 0.58 | 0.83 |
| cg21176263 | LMX1B | 9q33.3 | 2.65 × 10⁻⁹ | 2.29 × 10⁻³ | 64.92 | 72.69 | -7.77 | 0.75 | 0.62 | 0.87 |
| cg09021274 | DLG2 | 11q14.1 | 2.98 × 10⁻⁹ | 2.58 × 10⁻³ | 84.55 | 79.06 | 5.50 | 0.86 | 0.77 | 0.95 |
| cg17500968 | TRPV4 | 12q24.11 | 3.37 × 10⁻⁹ | 2.91 × 10⁻³ | 84.86 | 80.02 | 4.83 | 0.68 | 0.56 | 0.80 |
| cg09964361 | CAMK4 | 5q22.1 | 3.42 × 10⁻⁹ | 2.96 × 10⁻³ | 63.94 | 56.11 | 7.83 | 0.71 | 0.58 | 0.84 |
| cg07164211 | CACNA2D1 | 7q21.11 | 4.58 × 10⁻⁹ | 3.96 × 10⁻³ | 79.12 | 84.79 | -5.67 | 0.75 | 0.63 | 0.87 |
| cg14972143 | EIF4E | 4q23 | 4.70 × 10⁻⁹ | 4.07 × 10⁻³ | 16.56 | 11.10 | 5.46 | 0.84 | 0.74 | 0.94 |
| cg02695252 | PRLR | 5p13.2 | 4.71 × 10⁻⁹ | 4.07 × 10⁻³ | 72.17 | 78.88 | -6.71 | 0.81 | 0.70 | 0.92 |
| cg17369032 | NGFR | 17q21.33 | 5.51 × 10⁻⁹ | 4.76 × 10⁻³ | 75.13 | 81.46 | -6.33 | 0.77 | 0.65 | 0.89 |
| cg25067242 | NGF | 1p13.2 | 5.88 × 10⁻⁹ | 5.09 × 10⁻³ | 48.57 | 57.28 | -8.72 | 0.77 | 0.65 | 0.88 |
| cg25944168 | EIF2AK3 | 2p11.2 | 6.28 × 10⁻⁹ | 5.43 × 10⁻³ | 63.88 | 56.16 | 7.72 | 0.71 | 0.58 | 0.84 |
| cg21685789 | GABRG2 | 5q34 | 6.54 × 10⁻⁹ | 5.65 × 10⁻³ | 57.62 | 49.72 | 7.90 | 0.73 | 0.60 | 0.85 |
| cg06444178 | ANKH | 5p15.2 | 7.39 × 10⁻⁹ | 6.39 × 10⁻³ | 15.10 | 9.97 | 5.13 | 0.78 | 0.66 | 0.89 |
| cg17349736 | NR3C1 | 5q31.3 | 7.40 × 10⁻⁹ | 6.40 × 10⁻³ | 58.87 | 66.91 | -8.04 | 0.76 | 0.64 | 0.88 |
| cg21486834 | RHBDF2 | 17q25.1 | 8.81 × 10⁻⁹ | 7.62 × 10⁻³ | 81.54 | 86.77 | -5.23 | 0.76 | 0.64 | 0.87 |
| cg02111786 | NRXN3 | 14q24.3-q31.1 | 9.60 × 10⁻⁹ | 8.31 × 10⁻³ | 85.00 | 79.74 | 5.26 | 0.73 | 0.60 | 0.85 |
| cg14129053 | MYT1L | 2p25.3 | 1.02 × 10⁻⁸ | 8.86 × 10⁻³ | 72.28 | 66.10 | 6.18 | 0.65 | 0.53 | 0.77 |
| cg11590170 | GJA1 | 6q22.31 | 1.03 × 10⁻⁸ | 8.87 × 10⁻³ | 79.56 | 73.47 | 6.09 | 0.74 | 0.61 | 0.86 |
| cg21963925 | CACNA1H | 16p13.3 | 1.06 × 10⁻⁸ | 9.16 × 10⁻³ | 90.85 | 87.19 | 3.66 | 0.60 | 0.48 | 0.73 |
| cg24397382 | STX1A | 7q11.23 | 1.10 × 10⁻⁸ | 9.53 × 10⁻³ | 71.48 | 64.43 | 7.05 | 0.70 | 0.57 | 0.83 |
| cg15002761 | IGSF9B | 11q25 | 1.14 × 10⁻⁸ | 9.87 × 10⁻³ | 87.39 | 91.52 | -4.14 | 0.76 | 0.64 | 0.88 |
| cg22849544 | THRB | 3p24.2 | 1.16 × 10⁻⁸ | 1.00 × 10⁻² | 18.73 | 24.80 | -6.07 | 0.57 | 0.45 | 0.69 |
| cg12078872 | DDO | 6q21 | 1.33 × 10⁻⁸ | 1.15 × 10⁻² | 80.37 | 85.75 | -5.38 | 0.80 | 0.70 | 0.91 |
| ch.12.28033R | WNK1 | 12p13.33 | 1.48 × 10⁻⁸ | 1.28 × 10⁻² | 11.78 | 17.49 | -5.71 | 0.61 | 0.48 | 0.75 |
| cg23817893 | CCDC81 | 11q14.2 | 1.50 × 10⁻⁸ | 1.30 × 10⁻² | 42.44 | 50.91 | -8.46 | 0.75 | 0.63 | 0.87 |
| cg08408433 | PTGIR | 19q13.32 | 1.54 × 10⁻⁸ | 1.34 × 10⁻² | 71.09 | 77.74 | -6.65 | 0.72 | 0.59 | 0.84 |
| cg09713515 | DOCK4 | 7q31.1 | 1.56 × 10⁻⁸ | 1.35 × 10⁻² | 81.31 | 75.54 | 5.77 | 0.76 | 0.65 | 0.88 |
| cg00781169 | PTGER3 | 1p31.1 | 1.76 × 10⁻⁸ | 1.52 × 10⁻² | 60.07 | 67.91 | -7.85 | 0.70 | 0.57 | 0.83 |
| cg09070522 | REST | 4q12 | 1.91 × 10⁻⁸ | 1.66 × 10⁻² | 14.44 | 9.48 | 4.96 | 0.88 | 0.79 | 0.97 |
| cg09397542 | PHACTR1 | 6p24.1 | 1.97 × 10⁻⁸ | 1.70 × 10⁻² | 14.23 | 9.36 | 4.87 | 0.77 | 0.65 | 0.89 |
| cg23947039 | BDNF | 11p14.1 | 2.04 × 10⁻⁸ | 1.76 × 10⁻² | 7.56 | 3.79 | 3.78 | 0.87 | 0.78 | 0.96 |
| cg07539983 | SPARC | 5q33.1 | 2.17 × 10⁻⁸ | 1.88 × 10⁻² | 79.24 | 73.23 | 6.01 | 0.74 | 0.61 | 0.86 |
| cg02726883 | NF1 | 17q11.2 | 2.37 × 10⁻⁸ | 2.05 × 10⁻² | 14.59 | 9.63 | 4.96 | 0.91 | 0.84 | 0.99 |
| cg05931684 | EHMT2 | 6p21.33 | 2.48 × 10⁻⁸ | 2.15 × 10⁻² | 17.20 | 11.93 | 5.27 | 0.86 | 0.76 | 0.95 |
| cg10439765 | SLC12A5 | 20q13.12 | 2.61 × 10⁻⁸ | 2.26 × 10⁻² | 15.53 | 21.81 | -6.27 | 0.70 | 0.57 | 0.82 |
| cg19621317 | ASIC1 | 12q13.12 | 2.63 × 10⁻⁸ | 2.27 × 10⁻² | 71.62 | 78.17 | -6.56 | 0.80 | 0.70 | 0.91 |
| cg19753937 | NRG1 | 8p12 | 2.63 × 10⁻⁸ | 2.28 × 10⁻² | 74.82 | 80.88 | -6.06 | 0.73 | 0.61 | 0.85 |
| cg08644772 | IKBKAP | [9q31.3,](https://www.omim.org/geneMap/9/392?start=-3&limit=10&highlight=392) | 2.82 × 10⁻⁸ | 2.44 × 10⁻² | 83.19 | 87.94 | -4.75 | 0.76 | 0.64 | 0.88 |
| cg22952017 | CTSS | 1q21.3 | 2.99 × 10⁻⁸ | 2.59 × 10⁻² | 31.95 | 25.90 | 6.05 | 0.76 | 0.65 | 0.87 |
| cg18793036 | MME | 3q25.2 | 3.02 × 10⁻⁸ | 2.61 × 10⁻² | 14.89 | 21.02 | -6.12 | 0.65 | 0.52 | 0.79 |
| cg09045305 | ADARB2 | 10p15.3 | 3.20 × 10⁻⁸ | 2.77 × 10⁻² | 69.88 | 76.54 | -6.66 | 0.74 | 0.62 | 0.86 |
| cg21295398 | BECN1 | 17q21.31 | 3.33 × 10⁻⁸ | 2.88 × 10⁻² | 74.35 | 67.82 | 6.53 | 0.83 | 0.73 | 0.94 |
| cg08914905 | PIK3C3 | 18q12.3 | 3.43 × 10⁻⁸ | 2.97 × 10⁻² | 75.40 | 68.99 | 6.41 | 0.71 | 0.58 | 0.84 |
| cg01183713 | ULK4 | 3p22.1 | 3.50 × 10⁻⁸ | 3.03 × 10⁻² | 67.27 | 74.21 | -6.94 | 0.71 | 0.59 | 0.84 |
| cg19137569 | KCNN3 | 1q21.3 | 3.52 × 10⁻⁸ | 3.04 × 10⁻² | 32.61 | 40.65 | -8.04 | 0.78 | 0.66 | 0.89 |
| cg22762326 | OXR1 | 8q23.1 | 3.59 × 10⁻⁸ | 3.10 × 10⁻² | 82.45 | 76.99 | 5.46 | 0.75 | 0.62 | 0.87 |
| cg16298405 | RUNX2 | 6p21.1 | 3.85 × 10⁻⁸ | 3.33 × 10⁻² | 9.95 | 14.56 | -4.61 | 0.76 | 0.66 | 0.87 |
| cg06069187 | SARM1 | 17q11.2 | 4.15 × 10⁻⁸ | 3.59 × 10⁻² | 21.62 | 28.62 | -7.00 | 0.67 | 0.54 | 0.80 |
| cg18501142 | MALAT1 | 11q13.1 | 4.46 × 10⁻⁸ | 3.86 × 10⁻² | 18.71 | 13.32 | 5.39 | 0.79 | 0.68 | 0.90 |
| cg17457918 | SCN1A | 2q24.3 | 4.48 × 10⁻⁸ | 3.87 × 10⁻² | 41.52 | 49.70 | -8.19 | 0.73 | 0.60 | 0.85 |
| cg05337454 | NOS3 | 7q36.1 | 4.58 × 10⁻⁸ | 3.97 × 10⁻² | 88.15 | 83.70 | 4.45 | 0.70 | 0.57 | 0.83 |
| cg03786924 | KCNQ5 | 6q13 | 4.94 × 10⁻⁸ | 4.27 × 10⁻² | 29.22 | 22.61 | 6.61 | 0.74 | 0.62 | 0.86 |
| cg17403731 | HCN2 | 19p13.3 | 5.12 × 10⁻⁸ | 4.43 × 10⁻² | 67.65 | 60.59 | 7.05 | 0.70 | 0.57 | 0.83 |
| cg26701226 | WSCD1 | 17p13.2 | 5.62 × 10⁻⁸ | 4.86 × 10⁻² | 4.69 | 8.29 | -3.60 | 0.57 | 0.44 | 0.69 |
| cg06422471 | SHANK3 | 22q13.33 | 5.69 × 10⁻⁸ | 4.93 × 10⁻² | 21.70 | 15.91 | 5.80 | 0.71 | 0.58 | 0.83 |
