# Supplementary material for: Prenatal opioid exposure alters pain perception and increases long-term health risks in infants with neonatal opioid withdrawal syndrome
Source: Front Pain Res (Lausanne). 2025 Apr 17;6:1497801. doi: 10.3389/fpain.2025.1497801 (PMC12043715; doi:10.3389/fpain.2025.1497801)
Supplement: Supplementary file 6 [file Table2.docx]

**Supplementary Table S1**. Comprehensive list of pain-associated genes retrieved from published data and relevant references, compared with findings from our study.

| **Gene Pain associated** | **Reference** |
| --- | --- |
| ABAT | PMID:30277654 |
| ABCB1 | PMID:31755291 |
| ABCB1B |  |
| ABCC2 | PMID:22454423 |
| ABCC4 | PMID:22454423 |
| ABCG1 |  |
| AC005592.2 | PMID:34450027 |
| AC005592.3 | PMID:34450027 |
| ACAN | PMID:28742099 |
| ACE | PMID:19298544 |
| ACE2 | PMID:24275226 |
| ACO1 | PMID:25144566 |
| ADAMTS4 | PMID:28081267 |
| ADAMTS5 | PMID:28081267 |
| ADAMTSL1 | PMID:22678113 |
| ADAMTSL4 |  |
| ADARB2 | PMID:22678113 |
| ADCYAP1 |  |
| ADCYAP1R1 | PMID:25169732 |
| ADH1B | PMID:30371560 |
| ADORA1 |  |
| ADORA2A |  |
| ADORA2A-AS1 | PMID:17257240 |
| ADORA3 |  |
| ADRA1A | PMID:19565482 |
| ADRA1D | PMID:30904518 |
| ADRA2A | PMID:27805929 |
| ADRA2C |  |
| ADRB1 | PMID:26694722 |
| ADRB2 | PMID:31285095 |
| AGTR2 |  |
| AHR |  |
| AJAP1 |  |
| ALDH1A2 | PMID:34450027 |
| ALOX12 |  |
| AMIGO3 | PMID:32587327 |
| ANAPC4 | PMID:31194737 |
| ANKH |  |
| ANKK1 | PMID:22875483 |
| ANKTM1 |  |
| ANRIL |  |
| AOC1 | PMID:34450027 |
| APOA1BP |  |
| APOE | PMID:21453125 |
| APOL3 |  |
| APP |  |
| AQP2 | PMID:32130259 |
| AQP4 |  |
| AQP5-AS1 | PMID:32130259 |
| AQPEP | PMID:34450027 |
| AR |  |
| ARID4A | PMID:32632093 |
| ARL14EP | PMID:32121467 |
| ARL14EP-DT | PMID:32121467 |
| ARMC12 | PMID:23707272 |
| ARMS2 |  |
| ARNTL |  |
| ARRB2 | PMID:23167532 |
| ARVCF | PMID:31285095 |
| ASIC1 |  |
| ASIC3 |  |
| ASTN1 | PMID:22678113 |
| ASTN2 | PMID:34450027 |
| ATAT1 |  |
| ATF4 |  |
| ATG5 |  |
| ATL1 |  |
| ATP1A2 |  |
| ATP2C1 | PMID:33926923 |
| ATP2C2 | PMID:18676988 |
| ATP5B |  |
| ATXN1 | PMID:18403122 |
| AVIL |  |
| AVPR1A | PMID:24373571 |
| B2M | PMID:25144566 |
| BACE1 |  |
| BAHCC1 | PMID:28081371 |
| BBX | PMID:31194737 |
| BDKRB1 |  |
| BDKRB2 |  |
| BDNF | PMID:32736598 |
| BDNF-AS | PMID:19517061 |
| BECN1 |  |
| BEGAIN |  |
| BHLHA9 |  |
| BMP5 | PMID:34450027 |
| BMP6 | PMID:34450027 |
| BRWD1 | PMID:34450027 |
| BUD23 | PMID:22250207 |
| C12orf60 | PMID:34450027 |
| C12orf65 | PMID:34450027 |
| C17orf67 | PMID:34450027 |
| C1orf177 | PMID:34450027 |
| C21orf91 |  |
| C2orf40 | PMID:34450027 |
| C2orf41 | PMID:34450027 |
| C6orf106 | PMID:33830993 |
| C7orf10 |  |
| C7orf50 | PMID:25169732 |
| C8orf34 | PMID:34450027 |
| CA10; snoZ178 | PMID:33830993 |
| CACNA1A | PMID:19429006 |
| CACNA1B |  |
| CACNA1C |  |
| CACNA1E | PMID:28573794 |
| CACNA1F |  |
| CACNA1H |  |
| CACNA1I |  |
| CACNA1S |  |
| CACNA2D2 | PMID:23402298 |
| CACNA2D3 | PMID:22678113 |
| CACNB2 | PMID:18676988 |
| CACNB3 |  |
| CACNG2 | PMID:30371558 |
| CALCA | PMID:29148033 |
| CALCRL | PMID:25169732 |
| CAMK2A |  |
| CAMK4 | PMID:22074755 |
| CAMKIV |  |
| CAPN1 |  |
| CARF | PMID:32046629 |
| CASC16 | PMID:31706190 |
| CASP9 |  |
| CBR3-AS1 | PMID:31056713 |
| CBS | PMID:21402364 |
| CCDC190 | PMID:21905019 |
| CCDC81 |  |
| CCKBR |  |
| CCL2 |  |
| CCM2L |  |
| CCR2 | PMID:19559392 |
| CCT5 |  |
| CD247 | PMID:26872611 |
| CDCA3 | PMID:20102604 |
| CDH12 | PMID:22678113 |
| CDH18 | PMID:28051079 |
| CDK5R1 |  |
| CDKL5 |  |
| CEP120 | PMID:31194737 |
| CFAP418-AS1 | PMID:31903573 |
| CFDP1 | PMID:32632093 |
| CFTR |  |
| CGRP |  |
| CHRM2 | PMID:24275226 |
| CHRM3 | PMID:21570824 |
| CHRNA1 | PMID:22678113 |
| CHRNA3 | PMID:31362771 |
| CHRNA5 | PMID:31362771 |
| CHST3 | PMID:34450027 |
| CLCN6 |  |
| CLIC1 |  |
| CLIC4 |  |
| CLIC5 | PMID:18676988 |
| CLOCK | PMID:25169732 |
| CN5A |  |
| CNR1 | PMID:19539700 |
| CNR2 |  |
| COL11A1 | PMID:34450027 |
| COL27A1 | PMID:34450027 |
| COL4A1 | PMID:32632093 |
| COLGALT2 | PMID:34450027 |
| COMT | PMID:22718527 |
| COX1 |  |
| COX2 |  |
| CP | PMID:25144566 |
| CPQ |  |
| CRADD | PMID:34450027 |
| CRHBP | PMID:26447706 |
| CRHR2 | PMID:22074755 |
| CRIP2 |  |
| CSF1 |  |
| CSF2 |  |
| CSNK1D |  |
| CTC-537E7.1 | PMID:34450027 |
| CTD-2334D19.1;AC008565.1 | PMID:33830993 |
| CTD-2623N2.11 | PMID:34450027 |
| CTNNA2 | PMID:31194737 |
| CTSB |  |
| CTSG | PMID:26270939 |
| CTSS |  |
| CUX1 | PMID:34450027 |
| CX3CL1 |  |
| CX3CR1 |  |
| CXCL8 |  |
| CYP19A1 | PMID:22511967 |
| CYP1A2 | PMID:20652353 |
| CYP2B6 | PMID:31056713 |
| CYP2D6 |  |
| CYP3A4 | PMID:30381583 |
| DAN |  |
| DAO |  |
| DBH | PMID:19152006 |
| DBH-AS1 | PMID:26868704 |
| DCC | PMID:30261039 |
| DCDC1 | PMID:29995844 |
| DDO |  |
| DGKI | PMID:34450027 |
| DICER1 |  |
| DLG2 |  |
| DMD | PMID:28081371 |
| DMPIEZO |  |
| DNAJA3 | PMID:32632093 |
| DNMT1 |  |
| DOCK4 |  |
| DPP4 |  |
| DRD1 |  |
| DRD2 | PMID:31144779 |
| DRD3 | PMID:19464960 |
| DRD4 |  |
| DYNC1I1 | PMID:31194737 |
| EAR2 |  |
| ECM1 | PMID:32587327 |
| EDN1 |  |
| EDNRA | PMID:25169732 |
| EDNRB | PMID:19661472 |
| EFNB1 |  |
| EFNB2 | PMID:33021770 |
| EGR1 |  |
| EHBP1L1 | PMID:34450027 |
| EHBP1L2 | PMID:34450027 |
| EHMT2 |  |
| EIF2 |  |
| EIF2AK2 |  |
| EIF2AK3 |  |
| EIF4E |  |
| EIF4EBP1 |  |
| ELAC2 | PMID:21402364 |
| ENPP1 | PMID:27519661 |
| ENT1 |  |
| EREG | PMID:24275226 |
| ERG | PMID:34450027 |
| ESR1 | PMID:24698360 |
| ESR2 | PMID:19093296 |
| ESRRB | PMID:26584852 |
| EXD3 | PMID:33830993 |
| EXT2 |  |
| F2 | PMID:25158985 |
| F2RL1 |  |
| F5 | PMID:25158985 |
| FAAH | PMID:31335650 |
| FABP3P2 | PMID:34450027 |
| FAF1 | PMID:33830993 |
| FAF2 | PMID:32587327 |
| FAM101A | PMID:34450027 |
| FAM134B |  |
| FAM183B |  |
| FANCL | PMID:34450027 |
| FGF10 | PMID:31574782 |
| FGF13 | PMID:31574782 |
| FGF18 | PMID:34450027 |
| FGF2 |  |
| FGF3 | PMID:31574782 |
| FGF6 |  |
| FHL5 | PMID:32632093 |
| FILIP1 | PMID:34450027 |
| FKBP5 | PMID:30150364 |
| FNDC3B | PMID:31903573 |
| FOXP2 | PMID:31194737 |
| FPR1 |  |
| FRMD4A |  |
| FSHR | PMID:19093296 |
| FSTL4 |  |
| FTO | PMID:34450027 |
| FUT9 |  |
| GABBR1 |  |
| GABRA3 | PMID:24040174 |
| GABRB1 | PMID:17655760 |
| GABRB2 | PMID:31194737 |
| GABRB3 |  |
| GABRG2 |  |
| GABRR1 | PMID:28699326 |
| GAL |  |
| GALR1 |  |
| GALR2 |  |
| GAS5 |  |
| GBP1 |  |
| GCH1 | PMID:17057711 |
| GDF5 | PMID:34450027 |
| GDF6 | PMID:34450027 |
| GDF7 | PMID:34450027 |
| GFAP |  |
| GFRA2 |  |
| GJA1 |  |
| GLIS3 | PMID:34450027 |
| GLIS4 | PMID:34450027 |
| GNA11 |  |
| GNAO1 |  |
| GNAQ |  |
| GNAS | PMID:17388805 |
| GNAZ |  |
| GPD2 | PMID:28051079 |
| GPR149 |  |
| GPR34 |  |
| GPR55 |  |
| GRIA1 | PMID:32046629 |
| GRIA3 | PMID:24275226 |
| GRIN1 |  |
| GRIN2A | PMID:24275226 |
| GRIN2B |  |
| GRK2 |  |
| GRK3 | PMID:30904518 |
| GRK5 | PMID:24275226 |
| GRM1 |  |
| GRM5 |  |
| GRM7 | PMID:22678113 |
| GSDMC | PMID:30747904 |
| GSTM1 |  |
| H1F0 | PMID:34450027 |
| H2TRA |  |
| HAMLET |  |
| HCN2 |  |
| HCRTR1 | PMID:21344296 |
| HCRTR2 | PMID:26289589 |
| HDAC9 | PMID:34450027 |
| HDC |  |
| HEY2 |  |
| HFE | PMID:34450027 |
| HIC1 | PMID:32632093 |
| HLA-B |  |
| HLA-DPA1 | PMID:34450027 |
| HLA-DQ |  |
| HLA-DRB1 |  |
| HMOX2 |  |
| HOTAIR |  |
| HOXB8 |  |
| HPSE2 | PMID:32632093 |
| HRH1 |  |
| HRH2 |  |
| HSN2 |  |
| HTR1A | PMID:30904518 |
| HTR2A | PMID:33171011 |
| HTR2C | PMID:21614492 |
| HTR3A | PMID:30904518 |
| HTR3B | PMID:21570824 |
| HTR7 |  |
| HTRA1 | PMID:30747904 |
| IAPP |  |
| ICA1 |  |
| ICA1L | PMID:32632093 |
| ICAM1 | PMID:25145994 |
| IFNG | PMID:28651128 |
| IFNGR |  |
| IFRD1 | PMID:24275226 |
| IGSF9B |  |
| IKBKAP |  |
| IL10 | PMID:22074755 |
| IL10R1 |  |
| IL10RB | PMID:23852407 |
| IL11 | PMID:34450027 |
| IL12B | PMID:32620160 |
| IL13 | PMID:25304131 |
| IL16 | PMID:20662556 |
| IL18R1 |  |
| IL18RAP | PMID:23522322 |
| IL19 | PMID:29636026 |
| IL1A | PMID:28081267 |
| IL1B | PMID:27649267 |
| IL1R1 | PMID:22515947 |
| IL1R2 | PMID:24411993 |
| IL1RN | PMID:25207923 |
| IL23 |  |
| IL23R |  |
| IL37 | PMID:23317890 |
| IL4 | PMID:32141366 |
| IL6 | PMID:27048515 |
| IL6-AS1 | PMID:15733644 |
| IL6ST |  |
| IL8 |  |
| IL9 | PMID:19559392 |
| ILRUN | PMID:31194737 |
| INSR | PMID:18455362 |
| IQGAP1 | PMID:31903573 |
| IRAG1 | PMID:32632093 |
| IRF2BP1 | PMID:34450027 |
| IRS1 |  |
| ITGB2 |  |
| ITGB5 | PMID:32632093 |
| ITIH1 | PMID:34450027 |
| ITIH2 | PMID:34450027 |
| ITPK1 |  |
| JAG1 |  |
| JAKMIP3 | PMID:30747904 |
| KCNA1 | PMID:25599232 |
| KCNA2 |  |
| KCNAB3 |  |
| KCNB2 | PMID:18676988 |
| KCND3 | PMID:31194737 |
| KCNG4 | PMID:32697988 |
| KCNJ2 |  |
| KCNJ3 | PMID:25599232 |
| KCNJ6 | PMID:31269327 |
| KCNK18 |  |
| KCNK2 | PMID:24275226 |
| KCNK5 | PMID:27322543 |
| KCNK9 | PMID:25599232 |
| KCNMA1 | PMID:34450027 |
| KCNN3 | PMID:22030984 |
| KCNQ2 |  |
| KCNQ3 | PMID:18676988 |
| KCNQ4 |  |
| KCNQ5 |  |
| KCNS1 | PMID:24392765 |
| KDM2A | PMID:34450027 |
| KDR | PMID:31118800 |
| KIAA0040 | PMID:32632093 |
| KIF1A |  |
| KLF11 |  |
| KNDC1 | PMID:31194737 |
| LAMB3 | PMID:26566055 |
| LDLR |  |
| LEMD2 | PMID:33830993 |
| LEP |  |
| LEPREL1 | PMID:34450027 |
| LINC00342 | PMID:32632093 |
| LINC00568;RP11-54A4.2 | PMID:33830993 |
| LINC00842 | PMID:31903573 |
| LINC01347 | PMID:31194737 |
| LINC01572 | PMID:32246137 |
| LINC02029 | PMID:31903573 |
| LMX1B |  |
| LOC100287329 | PMID:30129153 |
| LOC100506 |  |
| LOC101448202 | PMID:25896984 |
| LOC101926964 | PMID:31903573 |
| LOC101927025 | PMID:31903573 |
| LOC101927066 | PMID:22103325 |
| LOC101927995 | PMID:27322543 |
| LOC101929309 | PMID:23707272 |
| LOC102724058 | PMID:24275226 |
| LOC105369501 | PMID:22875483 |
| LOC105369944 | PMID:25724697 |
| LOC105370032 | PMID:29470314 |
| LOC105370955 | PMID:21448238 |
| LOC105371394 | PMID:28051079 |
| LOC105371720 | PMID:27043930 |
| LOC105371818 | PMID:28081371 |
| LOC105372112 | PMID:21448238 |
| LOC105373313 | PMID:15147464 |
| LOC105373370 | PMID:24040174 |
| LOC105373786 | PMID:25169732 |
| LOC105373891 | PMID:31903573 |
| LOC105375078 | PMID:18676988 |
| LOC105375350 | PMID:19368856 |
| LOC105375457 | PMID:22074755 |
| LOC105375567 | PMID:25612138 |
| LOC105375629 | PMID:23793025 |
| LOC105375630 | PMID:25388962 |
| LOC105375655 | PMID:24674449 |
| LOC105375836 | PMID:22730276 |
| LOC105375897 | PMID:18676988 |
| LOC105376225 | PMID:32218487 |
| LOC105376360 | PMID:22678113 |
| LOC105377013 | PMID:32046629 |
| LOC105377703 | PMID:28051079 |
| LOC105377864 | PMID:17417740 |
| LOC105377951 | PMID:21622719 |
| LOC105377986 | PMID:32046629 |
| LOC105378525 | PMID:32046629 |
| LOC105378606 | PMID:32046629 |
| LOC105378841 | PMID:21905019 |
| LOC105379109 | PMID:31194737 |
| LOC105379318 | PMID:24974787 |
| LOC107985507 | PMID:25599232 |
| LOC107986832 | PMID:33021770 |
| LOC107986931 | PMID:22683712 |
| LOC112267867 | PMID:26872611 |
| LOC112267956 | PMID:23707272 |
| LOC112268294 | PMID:16960721 |
| LOC349160 | PMID:22074755 |
| LPAR1 |  |
| LPAR5 |  |
| LPP | PMID:34450027 |
| LRFN5 | PMID:31748543 |
| LRIG3 | PMID:34450027 |
| LRP1 | PMID:32632093 |
| LRRIQ3 |  |
| LTA | PMID:24959879 |
| LTBP1 | PMID:34450027 |
| LTBP2 | PMID:34450027 |
| MACC1-AS1 | PMID:21448238 |
| MAKP14 |  |
| MALAT1 |  |
| MAML2 | PMID:34450027 |
| MAML3 | PMID:31194737 |
| MAOA |  |
| MAOB |  |
| MAOB | PMID:16807522 |
| MAP1LC3B |  |
| MAP2K1 | PMID:23867732 |
| MAP2K6 | PMID:34450027 |
| MAPK1 | PMID:26872611 |
| MAPK10 |  |
| MAPK14 |  |
| MAPK3 |  |
| MAPK8 |  |
| MAPK9 |  |
| MARCHF4 | PMID:22683712 |
| MC1R |  |
| MC2R |  |
| MC4R |  |
| MDK |  |
| MECP2 |  |
| MED14 |  |
| MEF2D | PMID:32632093 |
| MEFV | PMID:23010357 |
| MGC4859 | PMID:29486785 |
| miR1 |  |
| miR124 |  |
| miR132 |  |
| miR16 |  |
| miR183 |  |
| miR195 |  |
| miR199a-3p |  |
| miR200 |  |
| miR206 |  |
| miR21 |  |
| miR212 |  |
| miR221 |  |
| miR222 |  |
| miR23a/b |  |
| miR29 |  |
| miR30 |  |
| miR339-3p |  |
| miR34 |  |
| miR34a |  |
| miR34b |  |
| miR365 |  |
| miR431 |  |
| miR451 |  |
| MIR4713HG | PMID:22511967 |
| miR499 |  |
| miR504 |  |
| miR551b-3p |  |
| miR7 |  |
| miR9 |  |
| MKK3 |  |
| MKK6 |  |
| MLLT10 | PMID:31194737 |
| MME |  |
| MMP1 |  |
| MMP13 |  |
| MMP16 |  |
| MMP17 | PMID:22683712 |
| MMP2 |  |
| MMP24 |  |
| MMP3 |  |
| MMP9 | PMID:31455415 |
| MN1 | PMID:34450027 |
| MNK1 |  |
| MNK2 |  |
| MNSOD |  |
| MPDZ | PMID:24275226 |
| MPPED2 | PMID:32632093 |
| MPZ |  |
| MRC2 |  |
| MRGPRE |  |
| MRVI1 |  |
| MSC-AS1 | PMID:24752136 |
| MSH2 | PMID:18676988 |
| MTA1 |  |
| MTDH | PMID:21448238 |
| MTHFD1 | PMID:21615938 |
| MTHFR | PMID:11121176 |
| MTHR |  |
| MTR | PMID:23430981 |
| MTRR | PMID:21615938 |
| MYD88 | PMID:26332828 |
| MYT1L | PMID:24582949 |
| N4BP1 |  |
| NA | PMID:33830993 |
| NACA2 | PMID:34450027 |
| NBR1 |  |
| NCAM1 | PMID:30747904 |
| NCOR2 | PMID:32632093 |
| NDN |  |
| NEAT1 |  |
| NEFM | PMID:34450027 |
| NF1 | PMID:22678113 |
| NFKB1A |  |
| NFKBIA | PMID:19773451 |
| NGF |  |
| NGF-AS1 | PMID:22074755 |
| NGFB |  |
| NGFR | PMID:21448238 |
| NGR2 |  |
| NLGN2 |  |
| NMRAL1 | PMID:34450027 |
| NMT1 | PMID:31194737 |
| NNMT | PMID:27726107 |
| NOG | PMID:34450027 |
| NOS1 | PMID:22074755 |
| NOS2 | PMID:22234503 |
| NOS3 | PMID:26098763 |
| NOTCH3 | PMID:20813781 |
| NOTCH4 |  |
| NPM1; FGF18 | PMID:33830993 |
| NPSR1 | PMID:25091462 |
| NPSR1-AS1 | PMID:25091462 |
| NPTX1 |  |
| NPY |  |
| NPY1R |  |
| NR3C1 | PMID:22074755 |
| NRG1 |  |
| NRIP1 | PMID:25315199 |
| NRXN3 |  |
| NSRP1 | PMID:19845785 |
| NTRK1 | PMID:23223113 |
| NTSR1 | PMID:24275226 |
| NTSR2 |  |
| NUMB | PMID:31194737 |
| OPRD1 | PMID:28084056 |
| OPRK1 | PMID:29120944 |
| OPRM1 | PMID:12357145 |
| OSM |  |
| OXR1 |  |
| OXT |  |
| P2RX3 |  |
| P2RX4 |  |
| P2RX7 | PMID:24934217 |
| P2RY12 |  |
| P2X3 |  |
| PACERR | PMID:23357220 |
| PANX1 |  |
| PARD6G | PMID:34450027 |
| PATJ | PMID:24275226 |
| PATL2 | PMID:25144566 |
| PAX5,LOC105376032 | PMID:29995844 |
| PCP2 | PMID:29884837 |
| PCSK5 |  |
| PCSK6 | PMID:22440827 |
| PDE10A |  |
| PDGFC | PMID:26872611 |
| PDYN |  |
| PDYN-AS1 | PMID:22730276 |
| PENK |  |
| PER2 |  |
| PGAM1P1;PLK2 | PMID:33830993 |
| PGK1 |  |
| PGR | PMID:25494303 |
| PHACTR1 | PMID:32632093 |
| PIK3C2G | PMID:26872611 |
| PIK3C3 |  |
| PIP5K1C |  |
| PIRT |  |
| PJA2 | PMID:31903573 |
| PLCB1 |  |
| PLCB3 |  |
| PLCE1 | PMID:32632093 |
| PLP1 |  |
| PNOC |  |
| PNPLA3 |  |
| POC5 | PMID:32632093 |
| POLD3 | PMID:34450027 |
| POLD4 | PMID:34450027 |
| POLE | PMID:24275226 |
| POLR1C | PMID:20482220 |
| POMC | PMID:19723618 |
| PON1 | PMID:20407783 |
| PPARA |  |
| PPP1R14C |  |
| PRDM12 |  |
| PRDM16 | PMID:32632093 |
| PRKAR1B |  |
| PRKCA | PMID:28051079 |
| PRKCA |  |
| PRKCB |  |
| PRKCD |  |
| PRKCG |  |
| PRKCG |  |
| PRKCQ |  |
| PRKCZ |  |
| PRKG1 |  |
| PRKN | PMID:22993228 |
| PRLR |  |
| PROK2 |  |
| PROKR1 |  |
| PRRT2 |  |
| PRRX1 |  |
| PRSS1 |  |
| PRX |  |
| PTGER1 |  |
| PTGER3 |  |
| PTGIR |  |
| PTGS1 | PMID:24275226 |
| PTGS2 | PMID:26081267 |
| PTN |  |
| PTPN5 |  |
| PTPRJ | PMID:34450027 |
| RAB7A |  |
| RAG1 |  |
| RALGPS1 | PMID:34450027 |
| RAMP1 | PMID:25169732 |
| RAPH1 | PMID:34450027 |
| REST |  |
| RETN | PMID:29649030 |
| RGS12 | PMID:22683712 |
| RGS9 |  |
| RHBDF2 |  |
| RLUA-1 |  |
| RLUA-2 |  |
| RNF123 | PMID:31194737 |
| RNF144B | PMID:34450027 |
| RNF213 | PMID:32632093 |
| RNU2-17P | PMID:34450027 |
| RNU2-40P | PMID:34450027 |
| RNU6-815P | PMID:34450027 |
| RNU6-962P | PMID:34450027 |
| RNU6-996P | PMID:34450027 |
| RORA | PMID:29884837 |
| RP11-115J23.1 | PMID:34450027 |
| RP11-123K19.1 | PMID:34450027 |
| RP11-274M4.1 | PMID:34450027 |
| RP11-281A20.2 | PMID:34450027 |
| RP11-284G10.1 | PMID:34450027 |
| RP11-290F24.3 | PMID:33830993 |
| RP11-332M4.1 | PMID:34450027 |
| RP11-35O15.1 | PMID:34450027 |
| RP11-501E14.1 | PMID:34450027 |
| RP11-95P13.1 | PMID:34450027 |
| RP11-993B23.1 | PMID:34450027 |
| RP11-993B23.2 | PMID:34450027 |
| RP11-993B23.3 | PMID:34450027 |
| RP1-228P16.4 | PMID:34450027 |
| RPL19P11 | PMID:34450027 |
| RSU1 | PMID:31903573 |
| RTP4 | PMID:34450027 |
| RUNX1 |  |
| RUNX2 | PMID:34450027 |
| S100A10 |  |
| SARM1 |  |
| SCH1 |  |
| SCN10A | PMID:31642403 |
| SCN11A | PMID:28953656 |
| SCN12A |  |
| SCN1A | PMID:24275226 |
| SCN1A-AS1 | PMID:20212137 |
| SCN2A |  |
| SCN3A |  |
| SCN4A |  |
| SCN5A | PMID:18676988 |
| SCN7A |  |
| SCN8A |  |
| SCN9A | PMID:26752484 |
| SCN9A,SCN1A-AS1 | PMID:26752484 |
| SCNN1A | PMID:25169732 |
| SDK1 | PMID:31194737 |
| SEPT9 |  |
| SERPINA1 | PMID:34450027 |
| SERPINA6 | PMID:19723618 |
| SFRP1 | PMID:30431558 |
| SHANK3 |  |
| SHMT1 | PMID:21615938 |
| SIGMAR1 | PMID:30266269 |
| SLC10A7 | PMID:33021770 |
| SLC11A2 | PMID:25144566 |
| SLC12A2 |  |
| SLC12A5 |  |
| SLC17A6 |  |
| SLC17A8 | PMID:22678113 |
| SLC24A3 | PMID:32632093 |
| SLC24A3;Â AL121761.1 | PMID:33830993 |
| SLC24A4 |  |
| SLC25A3 | PMID:34854908 |
| SLC27A6 | PMID:34450027 |
| SLC2A1 |  |
| SLC39A8 | PMID:34450027 |
| SLC39A9 | PMID:34450027 |
| SLC44A2 | PMID:34450027 |
| SLC6A2 | PMID:29723560 |
| SLC6A3 | PMID:29723560 |
| SLC6A4 | PMID:30904518 |
| SLCO1A2 |  |
| SMAD3 | PMID:34450027 |
| SMAD4 | PMID:34450027 |
| SMAL |  |
| SMG6 | PMID:34450027 |
| SMO | PMID:34450027 |
| SNAP25 | PMID:24885975 |
| SNAP47 | PMID:34450027 |
| SNX8 | PMID:29884837 |
| SOD2 | PMID:25818327 |
| SORCS3 | PMID:31194737 |
| SORT1 |  |
| SOX10 |  |
| SOX11 | PMID:33830993 |
| SOX5 | PMID:34450027 |
| SOX6 | PMID:34450027 |
| SP4 | PMID:31194737 |
| SPAG17 | PMID:34450027 |
| SPARC |  |
| SPINK1 |  |
| SPOCK2 | PMID:33021770 |
| SPON1 | PMID:21622719 |
| SPP1 |  |
| SPTLC1 |  |
| SPTLC2 |  |
| SQSTM1 |  |
| ST8SIA1 |  |
| STAG1 | PMID:31194737 |
| STAT6 | PMID:23793025 |
| STX1A | PMID:25169732 |
| SUGCT | PMID:32632093 |
| SUV39H2 | PMID:26220684 |
| SVEP1 | PMID:22678113 |
| SYN3 | PMID:28081267 |
| SYNE1 | PMID:25315199 |
| SYT16 | PMID:25169732 |
| TAAR1 | PMID:21905019 |
| TAAR2 | PMID:21905019 |
| TAC1 | PMID:24275226 |
| TAC4 |  |
| TACC3 | PMID:34450027 |
| TACR1 | PMID:23167532 |
| TAOK3 | PMID:30031856 |
| TBC1D7 |  |
| TBK1 |  |
| TCL1A | PMID:22405131 |
| TCP11 | PMID:34450027 |
| TDAG8 |  |
| TEAD1 | PMID:34450027 |
| TF | PMID:25144566 |
| TFRC | PMID:29351172 |
| TG | PMID:28051079 |
| TGFA | PMID:34450027 |
| TGFB1 | PMID:34450027 |
| TGFB2 | PMID:34450027 |
| TGFBR2 |  |
| TH | PMID:30904518 |
| THRB |  |
| TIPIN | PMID:23867732 |
| TLR2 | PMID:27649267 |
| TLR4 | PMID:23023380 |
| TMEM16F |  |
| TMEM18 | PMID:34450027 |
| TMEM35A |  |
| TNC | PMID:34450027 |
| TNF | PMID:25315199 |
| TNFA |  |
| TNFRSF11B | PMID:26798969 |
| TNFRSF1A | PMID:30075559 |
| TNFRSF1B | PMID:30075559 |
| TPH1 | PMID:17194593 |
| TPH2 | PMID:20740293 |
| TPSAN9 |  |
| TRMT9B | PMID:21666692 |
| TRPA1 | PMID:29620434 |
| TRPC1 | PMID:22162417 |
| TRPC4 | PMID:22162417 |
| TRPC5 |  |
| TRPC7-AS2 | PMID:22162417 |
| TRPM2 |  |
| TRPM6 | PMID:22162417 |
| TRPM8 | PMID:31873179 |
| TRPV1 | PMID:31014225 |
| TRPV2 | PMID:27079220 |
| TRPV3 | PMID:22162417 |
| TRPV4 | PMID:22162417 |
| TSC2 |  |
| TSEN15 | PMID:34450027 |
| TSKU | PMID:34450027 |
| TSPAN2 |  |
| TSPO | PMID:25582579 |
| TSSC1 | PMID:34924555 |
| TUG1 |  |
| TXNRD2 | PMID:22337325 |
| UBAP2 | PMID:34450027 |
| UFL1-AS1 | PMID:22683712 |
| UGT2B7 | PMID:31607718 |
| ULK1 |  |
| ULK4 |  |
| USP8 | PMID:34450027 |
| USP9 | PMID:34450027 |
| UTRN | PMID:31194737 |
| UTS2 | PMID:27090416 |
| VDR | PMID:23984350 |
| VDR | PMID:29467039 |
| VEPH1 | PMID:25604633 |
| VGLL4 | PMID:34450027 |
| VGLUT2 |  |
| WNK1 |  |
| WSCD1 |  |
| WSCD2 | PMID:34450027 |
| WWP2 | PMID:32587327 |
| XIST |  |
| YAP1 | PMID:32632093 |
| ZCCHC14 |  |
| ZNF429 | PMID:19207018 |
| ZNF555 | PMID:22678113 |
| ZNF618 |  |
| ZSCAN20 | PMID:26629533 |
| ZSCAN25 | PMID:31269327 |
