# Supplementary material for: Prenatal opioid exposure alters pain perception and increases long-term health risks in infants with neonatal opioid withdrawal syndrome
Source: Front Pain Res (Lausanne). 2025 Apr 17;6:1497801. doi: 10.3389/fpain.2025.1497801 (PMC12043715; doi:10.3389/fpain.2025.1497801)
Supplement: Supplementary file 7 [file Table3.docx]

**Supplementary Table S2.** Pain-Related Genes list and their and their biological roles. This table provides an extensive list of genes associated with pain, highlighting their specific biological roles. The genes are categorized based on their involvement in various physiological and pathological processes, including diabetes, telomere maintenance, glucose metabolism, ageing, and suicide, among others. Each entry details the gene’s function and its relevance to the context of pain.

| **Gene** | **Genes  activity in** | **Genes  activity in** | **Genes  activity in** | **Genes  activity in** | **Genes  activity in** | **Genes  activity in** | **Genes  activity in** | **Genes  activity in** |
| --- | --- | --- | --- | --- | --- | --- | --- | --- |
| CACNA1C |  |  | Risk of suicide | Ion channel | Ageing | Oxidative Stress | Glucose metabolism | Circadian |
| KLF11 |  |  |  |  | Ageing | Oxidative Stress | Glucose metabolism | Circadian |
| NR3C1 | Inflammation | Cytokines | Risk of suicide |  | Ageing | Oxidative Stress | Glucose metabolism |  |
| EIF2AK3 |  |  |  |  | Ageing | Oxidative Stress | Glucose metabolism |  |
| CACNA1H |  |  |  | Ion channel |  |  | Glucose metabolism |  |
| CFTR |  | Transporter |  | Telomere Maintenance |  |  | Glucose metabolism |  |
| NOTCH3 |  |  |  |  |  |  | Glucose metabolism |  |
| GJA1 |  |  |  |  |  |  | Glucose metabolism |  |
| STX1A |  |  |  |  |  |  | Glucose metabolism |  |
| NOS3 | Mood and emotional |  | Risk of suicide | Telomere Maintenance |  | Oxidative Stress | Obesity | Antioxidant |
| TNF | Inflammation | Cytokines | Risk of suicide | glial cells control | Ageing | Oxidative Stress | Obesity | Antioxidant |
| BDNF |  | Cytokines | Risk of suicide | glial cells control | Ageing | Oxidative Stress | Obesity | Autophagy |
| ESR1 | Glucose metabolism | Cytokines | Risk of suicide | Telomere Maintenance | Ageing | Oxidative Stress | Obesity ; Antioxidant | Circadian |
| ESR2 | Antioxidant |  |  | Telomere Maintenance |  | Oxidative Stress | Obesity | Autophagy |
| OPRM1 | Mood and emotional |  | Risk of suicide |  | Ageing | Oxidative Stress | Obesity |  |
| ATP1A2 | Metabolism of salt |  |  |  | Ageing | Oxidative Stress | Obesity |  |
| LMX1B |  |  |  |  |  |  | Obesity | Autophagy |
| NPY |  |  |  |  |  | Oxidative Stress | Obesity |  |
| THRB |  |  |  |  |  |  | Obesity |  |
| NRXN3 |  |  |  |  |  |  | Obesity |  |
| SPARC |  |  |  |  |  |  | Obesity |  |
| MYT1L |  |  |  |  |  |  | Obesity |  |
| BECN1 |  |  |  |  | Ageing | Oxidative Stress | Antioxidant | Autophagy |
| IL1B | Inflammation | Cytokines | Depression |  |  | Oxidative Stress | Antioxidant | Circadian |
| OXR1 |  |  |  |  |  |  | Antioxidant |  |
| ARNTL |  |  |  |  | Ageing |  |  | Circadian |
| MAPK3 | Inflammation | Cytokines |  | Telomere Maintenance | Ageing |  |  | Autophagy |
| NGF |  | Cytokines | Risk of suicide |  | Ageing | Oxidative Stress |  |  |
| NRG1 |  | Cytokines |  |  | Ageing | Oxidative Stress |  |  |
| SCN8A | Metabolism of salt |  |  | Ion channel | Ageing | Oxidative Stress |  |  |
| GRIA1 |  |  | Risk of suicide |  | Ageing | Oxidative Stress |  |  |
| NGFR |  |  | Risk of suicide |  | Ageing |  |  |  |
| MTA1 |  |  |  | Telomere Maintenance | Ageing | Oxidative Stress |  |  |
| PRLR |  |  |  |  | Ageing | Oxidative Stress |  |  |
| EIF4E |  |  |  |  | Ageing | Oxidative Stress |  |  |
| CTSS |  |  |  |  | Ageing | Oxidative Stress |  | Autophagy |
| ULK1 |  |  |  |  | Ageing | Oxidative Stress |  | Autophagy |
| CAMK4 |  | Cytokines |  |  |  |  |  | Circadian |
| OSM |  | Cytokines |  |  |  |  |  | Circadian |
| ABCA1 |  | Transporter |  |  |  |  |  | Circadian |
| SHMT1 |  |  |  | Telomere Maintenance |  |  |  | Circadian |
| DOCK4 |  |  |  |  |  |  |  | Circadian |
| GNA11 |  |  |  |  |  |  |  | Circadian |
| CRIP2 |  |  |  |  |  |  |  | Circadian |
| IL23R | Inflammation | Cytokines |  |  |  |  |  |  |
| PTGER3 | Inflammation |  |  |  |  |  |  |  |
| PTGIR | Inflammation |  |  |  |  |  |  |  |
| TRPV4 |  | Cytokines |  |  |  |  |  |  |
| RUNX1 |  | Cytokines |  |  |  |  |  |  |
| CXCL8 |  | Cytokines |  |  |  | Oxidative Stress |  |  |
| IL18R1 |  | Cytokines |  |  |  |  |  |  |
| MALAT1 |  | Cytokines |  |  |  | Oxidative Stress |  |  |
| TGFBR2 |  | Cytokines |  |  |  |  |  |  |
| KCNJ2 | Metabolism of salt |  | Risk of suicide | Ion channel |  |  |  |  |
| SCN1A | Metabolism of salt |  |  | Ion channel |  |  |  |  |
| CLIC4 |  |  |  | Ion channel |  |  |  |  |
| KCNQ5 |  |  |  | Ion channel |  |  |  |  |
| ADORA2A |  |  | Risk of suicide |  |  |  |  |  |
| ANKK1 |  |  | Risk of suicide |  |  |  |  |  |
| DLG2 |  |  | Risk of suicide | Telomere Maintenance |  |  |  |  |
| GABRG2 |  |  | Risk of suicide |  |  |  |  |  |
| IGSF9B |  |  | Risk of suicide |  |  |  |  |  |
| PCSK5 |  |  | Risk of suicide |  |  |  |  |  |
| PRDM16 |  |  | Risk of suicide | Telomere Maintenance |  |  |  |  |
| ULK4 |  |  |  |  |  |  |  | Autophagy |
| NF1 |  |  |  |  |  |  |  |  |
| ATG5 |  |  |  |  |  |  |  | Autophagy |
| SHANK3 |  |  |  |  |  |  |  |  |
| AJAP1 |  |  |  |  |  |  |  |  |
| SLC12A5 |  | Transporter |  |  |  |  |  |  |
| WNK1 | Metabolism of salt |  |  |  |  |  |  |  |
| IKBKAP |  |  |  | Telomere Maintenance |  |  |  |  |
| RUNX2 |  |  |  | Telomere Maintenance |  |  |  |  |
| REST |  |  |  | Telomere Maintenance |  |  |  |  |
| PLCE1 |  |  |  | Telomere Maintenance |  |  |  |  |
| KCNAB3 | Memory |  |  |  |  |  |  |  |
| MAPK10 |  |  |  |  |  | Oxidative Stress |  |  |
| CASP9 |  |  |  |  |  | Oxidative Stress |  |  |
| N4BP1 |  |  |  |  |  |  |  |  |
| PRKAR1B |  |  |  |  |  |  |  |  |
| NLGN2 |  |  |  |  |  |  |  |  |
| PTN |  |  |  |  |  |  |  |  |
| PDE10A |  |  |  |  |  |  |  |  |
| CCDC81 |  |  |  |  |  |  |  |  |
| KCNN3 |  |  |  |  |  |  |  |  |
| C7orf10 |  |  |  |  |  |  |  |  |
| LPAR1 |  |  |  |  |  |  |  |  |
| GNAO1 |  |  |  |  |  |  |  |  |
| SARM1 |  |  |  |  |  |  |  |  |
| ADARB2 |  |  |  |  |  |  |  |  |
| ASIC1 |  |  |  |  |  |  |  |  |
| CPQ |  |  |  |  |  |  |  |  |
| MME |  |  |  |  |  |  |  |  |
| EDNRB |  |  |  |  |  |  |  |  |
| CACNA2D1 |  |  |  |  |  |  |  |  |
| DDO |  |  |  |  |  |  |  |  |
| RHBDF2 |  |  |  |  |  |  |  |  |
| WSCD1 |  |  |  |  |  |  |  |  |
| PHACTR1 |  |  |  |  |  |  |  |  |
| GRK5 |  |  |  |  |  |  |  |  |
| ANKH |  |  |  |  |  |  |  |  |
| EHMT2 |  |  |  |  |  |  |  |  |
| CAPN1 |  |  |  |  |  |  |  |  |
| GALR1 |  |  |  |  |  |  |  |  |
| TAOK3 |  |  |  |  |  |  |  |  |
| HCN2 |  |  |  |  |  |  |  |  |
| MRC2 |  |  |  |  |  |  |  |  |
| PRKG1 |  |  |  |  |  |  |  |  |
| CALCA |  |  |  |  |  |  |  |  |
| IL6ST |  |  | Depression |  |  |  |  |  |
| PIK3C3 |  |  |  | Telomere Maintenance |  |  |  | Autophagy |
