# Supplementary material for: Prenatal opioid exposure alters pain perception and increases long-term health risks in infants with neonatal opioid withdrawal syndrome
Source: Front Pain Res (Lausanne). 2025 Apr 17;6:1497801. doi: 10.3389/fpain.2025.1497801 (PMC12043715; doi:10.3389/fpain.2025.1497801)
Supplement: Supplementary file 8 [file Table4.docx]

**Supplementary Table-S3.** Investigation into +Opioids/+NOWS and +Opioids/-NOWS highlights distinct variations in the methylation status linked with NOWS-associated pain.

| **Target ID** | **Gene** | **location** | | **p-Val** | **FDR p-Val** | | **% Methylation** | | | **AUC** | **CI** | |
| --- | --- | --- | --- | --- | --- | --- | --- | --- | --- | --- | --- | --- |
|  |  |  |  |  |  |  | **Cases** | **Control** | **Difference** |  | **lower** | **upper** |
| cg16839955 | ARNTL | 11p15.3 | | 4.02774E-13 | 3.484E-07 | | 28.33 | 20.13 | 8.20 | 0.81 | 0.71 | 0.92 |
| cg15442907 | CACNA1C | 12p13.33 | | 5.63386E-13 | 4.87329E-07 | | 45.17 | 55.79 | -10.62 | 0.78 | 0.67 | 0.90 |
| cg25015598 | CTSS | 1q21.3 | | 2.34411E-12 | 2.02766E-06 | | 47.02 | 57.33 | -10.31 | 0.73 | 0.61 | 0.85 |
| cg15118537 | PRKG1 | 10q11.23-q21.1 | | 3.09484E-12 | 2.67704E-06 | | 61.39 | 70.67 | -9.27 | 0.71 | 0.58 | 0.84 |
| cg22623236 | PDE10A | 6q27 | | 9.31447E-12 | 8.05702E-06 | | 62.35 | 71.33 | -8.98 | 0.72 | 0.60 | 0.85 |
| cg09711113 | NLGN2 | 17p13.1 | | 1.10962E-11 | 9.59824E-06 | | 53.95 | 63.63 | -9.69 | 0.75 | 0.62 | 0.87 |
| cg22197205 | CLIC4 | 1p36.11 | | 1.82731E-11 | 1.58062E-05 | | 75.08 | 82.18 | -7.10 | 0.75 | 0.64 | 0.87 |
| cg08253824 | SCN8A | 12q13.13 | | 1.85455E-11 | 1.60418E-05 | | 60.21 | 69.29 | -9.09 | 0.71 | 0.59 | 0.84 |
| cg22533025 | CFTR | 7q31.2 | | 1.97867E-11 | 1.71155E-05 | | 72.00 | 79.58 | -7.59 | 0.73 | 0.61 | 0.85 |
| cg09021274 | DLG2 | 11q14.1 | | 4.16523E-11 | 3.60292E-05 | | 76.63 | 83.36 | -6.73 | 0.80 | 0.69 | 0.91 |
| cg03652989 | ULK1 | 12q24.33 | | 5.15017E-11 | 4.4549E-05 | | 80.91 | 74.25 | 6.66 | 0.77 | 0.65 | 0.89 |
| cg11978118 | ADORA2A | 22q11.23 | | 6.94793E-11 | 6.00996E-05 | | 71.99 | 79.40 | -7.40 | 0.77 | 0.66 | 0.89 |
| cg21517792 | MTA1 | 14q32.33 | | 1.14118E-10 | 9.87118E-05 | | 77.94 | 84.31 | -6.37 | 0.78 | 0.66 | 0.89 |
| cg09778136 | ATG5 | 6q21 | | 1.31802E-10 | 0.000114009 | | 49.51 | 58.94 | -9.42 | 0.75 | 0.63 | 0.87 |
| cg25885356 | RUNX1 | 21q22.12 | | 1.38771E-10 | 0.000120037 | | 33.21 | 42.45 | -9.24 | 0.70 | 0.58 | 0.83 |
| cg24062706 | OSM | 22q12.2 | | 1.59647E-10 | 0.000138095 | | 36.26 | 28.16 | 8.10 | 0.71 | 0.59 | 0.84 |
| cg20629735 | IL18R1 | [2q12.1](https://www.omim.org/geneMap/2/512?start=-3&limit=10&highlight=512) | | 1.7238E-10 | 0.000149109 | | 73.99 | 80.95 | -6.96 | 0.76 | 0.65 | 0.88 |
| cg12339476 | MAPK10 | 4q21.3 | | 2.33796E-10 | 0.000202234 | | 63.21 | 71.53 | -8.32 | 0.73 | 0.60 | 0.85 |
| cg09031352 | PTN | 7q33 | | 5.47185E-10 | 0.000473315 | | 38.19 | 47.35 | -9.16 | 0.75 | 0.63 | 0.87 |
| cg03402505 | GRIA1 | 5q33.2 | | 5.83728E-10 | 0.000504925 | | 57.48 | 49.10 | 8.38 | 0.78 | 0.67 | 0.90 |
| cg10274696 | C7orf10 | 7p14.1 | | 5.85945E-10 | 0.000506843 | | 64.73 | 72.72 | -7.99 | 0.78 | 0.67 | 0.90 |
| cg06942027 | KCNJ2 | 17q24.3 | | 6.04073E-10 | 0.000522523 | | 20.18 | 14.00 | 6.19 | 0.70 | 0.57 | 0.83 |
| cg12578250 | PRDM16 | 1p36.32 | | 1.09771E-09 | 0.00094952 | | 58.24 | 66.72 | -8.48 | 0.73 | 0.60 | 0.85 |
| cg02081889 | PCSK5 | [9q21.13](https://www.omim.org/geneMap/9/246?start=-3&limit=10&highlight=246) | | 1.31889E-09 | 0.001140836 | | 14.37 | 9.15 | 5.22 | 0.79 | 0.68 | 0.90 |
| cg15787454 | CPQ | 8q22.1 | | 1.53272E-09 | 0.001325802 | | 76.04 | 82.37 | -6.32 | 0.74 | 0.62 | 0.86 |
| cg16910896 | LPAR1 | 9q31.3 | | 1.54708E-09 | 0.001338224 | | 64.85 | 72.65 | -7.80 | 0.72 | 0.59 | 0.84 |
| cg12056044 | IL23R | 1p31.3 | | 1.78445E-09 | 0.001543547 | | 60.52 | 68.71 | -8.20 | 0.75 | 0.63 | 0.87 |
| cg13480658 | AJAP1 | 1p36.32 | | 2.04637E-09 | 0.001770113 | | 89.37 | 93.23 | -3.87 | 0.72 | 0.59 | 0.84 |
| cg07164211 | CACNA2D1 | 7q21.11 | 4.5838E-09 | | | 0.003964985 | 79.12 | 84.79 | -5.67 | 0.75 | 0.63 | 0.87 |
| cg02695252 | PRLR | 5p13.2 | 4.70645E-09 | | | 0.004071078 | 72.17 | 78.88 | -6.71 | 0.81 | 0.70 | 0.92 |
| cg21685789 | GABRG2 | 5q34 | 6.53503E-09 | | | 0.005652798 | 57.62 | 49.72 | 7.90 | 0.73 | 0.60 | 0.85 |
| cg13999099 | IL6ST | 5q11.2 | 6.94688E-09 | | | 0.006009049 | 26.34 | 19.75 | 6.59 | 0.82 | 0.71 | 0.92 |
| cg06444178 | ANKH | 5p15.2 | 7.38904E-09 | | | 0.006391524 | 15.10 | 9.97 | 5.13 | 0.78 | 0.66 | 0.89 |
| cg17349736 | NR3C1 | 5q31.3 | 7.40444E-09 | | | 0.00640484 | 58.87 | 66.91 | -8.04 | 0.76 | 0.64 | 0.88 |
| cg23817893 | CCDC81 | 11q14.2 | 1.50267E-08 | | | 0.012998088 | 42.44 | 50.91 | -8.46 | 0.75 | 0.63 | 0.87 |
| cg08408433 | PTGIR | 19q13.32 | 1.54471E-08 | | | 0.013361725 | 71.09 | 77.74 | -6.65 | 0.72 | 0.59 | 0.84 |
| cg21486834 | RHBDF2 | 17q25.1 | 1.77139E-08 | | | 0.015322544 | 81.29 | 86.42 | -5.13 | 0.75 | 0.63 | 0.87 |
| cg09397542 | PHACTR1 | 6p24.1 | 1.96983E-08 | | | 0.017038989 | 14.23 | 9.36 | 4.87 | 0.77 | 0.65 | 0.89 |
| cg23947039 | BDNF | 11p14.1 | 2.03594E-08 | | | 0.017610846 | 7.56 | 3.79 | 3.78 | 0.87 | 0.78 | 0.96 |
| cg05931684 | EHMT2 | 6p21.33 | 2.47996E-08 | | | 0.02145164 | 17.20 | 11.93 | 5.27 | 0.86 | 0.76 | 0.95 |
| cg19753937 | NRG1 | 8p12 | 2.63357E-08 | | | 0.022780393 | 74.82 | 80.88 | -6.06 | 0.73 | 0.61 | 0.85 |
| cg08644772 | IKBKAP | [9q31.3,](https://www.omim.org/geneMap/9/392?start=-3&limit=10&highlight=392) | 2.82121E-08 | | | 0.024403441 | 83.19 | 87.94 | -4.75 | 0.76 | 0.64 | 0.88 |
| cg25426313 | PLCE1 | 10q23.33 | 2.92429E-08 | | | 0.025295131 | 66.08 | 73.18 | -7.11 | 0.73 | 0.61 | 0.86 |
| cg15929698 | NPY | 7p15.3 | 2.94761E-08 | | | 0.025496799 | 41.65 | 49.94 | -8.29 | 0.74 | 0.62 | 0.86 |
| cg09045305 | ADARB2 | 10p15.3 | 3.1982E-08 | | | 0.027664446 | 69.88 | 76.54 | -6.66 | 0.74 | 0.62 | 0.86 |
| cg01183713 | ULK4 | 3p22.1 | 3.50134E-08 | | | 0.030286572 | 67.27 | 74.21 | -6.94 | 0.71 | 0.59 | 0.84 |
| cg09070522 | REST | 4q12 | 4.17062E-08 | | | 0.036075874 | 14.41 | 9.61 | 4.81 | 0.85 | 0.75 | 0.95 |
| cg18501142 | MALAT1 | 11q13.1 | 4.46028E-08 | | | 0.038581431 | 18.71 | 13.32 | 5.39 | 0.79 | 0.68 | 0.90 |
| cg17457918 | SCN1A | 2q24.3 | 4.47891E-08 | | | 0.038742591 | 41.52 | 49.70 | -8.19 | 0.73 | 0.60 | 0.85 |
| cg17001898 | RUNX2 | 6p21.1 | 4.70884E-08 | | | 0.04073149 | 78.84 | 84.22 | -5.38 | 0.73 | 0.60 | 0.85 |

**Supplementary Table-S4**. Examining the (+Opioids/+NOWS) group, (+Opioids/-NOWS) group, and the (-Opioids/-NOWS, control) group reveals CpG targets linked with pain.

| **Target ID** | **Gene** | **location** | **p-Val** | **FDR p-Val** | **% Methylation** | | | **AUC** | **CI** | |
| --- | --- | --- | --- | --- | --- | --- | --- | --- | --- | --- |
|  |  |  |  |  | **Cases** | **Control** | **Difference** |  | **lower** | **upper** |
| cg06976250 | ANKK1 | 11q23.2 | 5.98709E-12 | 5.17884E-06 | 16.26 | 10.96 | 5.30 | 0.59 | 0.46 | 0.71 |
| cg11211173 | ATP1A2 | 1q23.2 | 1.76303E-12 | 1.52502E-06 | 75.68 | 68.59 | 7.09 | 0.73 | 0.61 | 0.84 |
| cg21295398 | BECN1 | 17q21.31 | 1.59724E-08 | 0.01381612 | 73.79 | 67.82 | 5.97 | 0.79 | 0.68 | 0.89 |
| cg21963925 | CACNA1H | 16p13.3 | 1.05854E-08 | 0.00915637 | 90.85 | 87.19 | 3.66 | 0.60 | 0.48 | 0.73 |
| cg04708753 | CASP9 | 1p36.21 | 1.06846E-13 | 9.24216E-08 | 5.83 | 11.12 | -5.28 | 0.61 | 0.49 | 0.74 |
| cg26635219 | CFTR | 7q31.2 | 4.77178E-13 | 4.12759E-07 | 20.30 | 14.19 | 6.11 | 0.64 | 0.52 | 0.76 |
| cg22952017 | CTSS | 1q21.3 | 2.98848E-08 | 0.02585036 | 31.95 | 25.90 | 6.05 | 0.76 | 0.65 | 0.87 |
| cg18302652 | CXCL8 | 4q13.3 | 1.23658E-09 | 0.00106964 | 15.36 | 21.35 | -5.99 | 0.65 | 0.53 | 0.77 |
| cg07444152 | EHMT2 | 6p21.33 | 1.37817E-10 | 0.00011921 | 75.68 | 69.17 | 6.50 | 0.72 | 0.60 | 0.83 |
| cg20583095 | ESR1 | 6q25.1-q25.2 | 3.1144E-08 | 0.02693953 | 32.60 | 26.51 | 6.09 | 0.73 | 0.62 | 0.85 |
| cg03659519 | GALR1 | 18q23 | 5.45115E-37 | 4.71524E-31 | 17.00 | 10.84 | 6.16 | 0.68 | 0.56 | 0.80 |
| cg22018329 | GNA11 | 19p13.3 | 3.13904E-11 | 2.71527E-05 | 84.28 | 78.79 | 5.49 | 0.71 | 0.60 | 0.83 |
| cg25387779 | GNAO1 | 16q13 | 2.29448E-09 | 0.00198472 | 20.01 | 26.52 | -6.51 | 0.60 | 0.48 | 0.73 |
| cg00248439 | GRK5 | 10q26.11 | 3.87423E-10 | 0.00033512 | 89.13 | 84.80 | 4.34 | 0.67 | 0.55 | 0.79 |
| cg17403731 | HCN2 | 19p13.3 | 1.57481E-08 | 0.01362207 | 67.09 | 60.59 | 6.49 | 0.69 | 0.57 | 0.81 |
| cg14117934 | IL1B | 2q14.1 | 1.8592E-09 | 0.00160821 | 75.51 | 69.36 | 6.14 | 0.75 | 0.64 | 0.86 |
| cg13999099 | IL6ST | 5q11.2 | 1.24975E-11 | 1.08103E-05 | 23.12 | 16.95 | 6.17 | 0.78 | 0.68 | 0.89 |
| cg13377102 | KCNAB3 | 17p13.1 | 9.13633E-16 | 7.90293E-10 | 71.71 | 79.30 | -7.59 | 0.75 | 0.64 | 0.86 |
| cg19137569 | KCNN3 | 1q21.3 | 1.18661E-09 | 0.00102642 | 32.94 | 40.65 | -7.70 | 0.77 | 0.67 | 0.88 |
| cg15677797 | KLF11 | 2p25.1 | 7.74444E-21 | 6.69894E-15 | 6.37 | 13.28 | -6.91 | 0.57 | 0.45 | 0.69 |
| cg23837895 | LMX1B | 9q33.3 | 2.99394E-09 | 0.00258976 | 42.44 | 35.43 | 7.02 | 0.70 | 0.59 | 0.82 |
| cg24368031 | MRC2 | 17q23.2 | 5.29246E-08 | 0.04577980 | 26.48 | 20.97 | 5.51 | 0.70 | 0.59 | 0.82 |
| cg14129053 | MYT1L | 2p25.3 | 1.02399E-08 | 0.00885747 | 72.28 | 66.10 | 6.18 | 0.65 | 0.53 | 0.77 |
| cg00263307 | NF1 | 17q11.2 | 1.59871E-10 | 0.00013829 | 80.71 | 74.86 | 5.85 | 0.73 | 0.62 | 0.84 |
| cg25067242 | NGF | 1p13.2 | 1.96948E-08 | 0.01703600 | 50.02 | 57.28 | -7.27 | 0.73 | 0.62 | 0.85 |
| cg02904605 | NOTCH3 | 19p13.12 | 1.07203E-12 | 9.27307E-07 | 67.38 | 74.86 | -7.48 | 0.72 | 0.60 | 0.83 |
| cg03349819 | NRG1 | 8p12 | 5.43655E-08 | 0.04702613 | 19.48 | 25.36 | -5.88 | 0.64 | 0.52 | 0.76 |
| cg16243402 | **OPRM1** | [6q25.2](https://www.omim.org/geneMap/6/964?start=-3&limit=10&highlight=964) | 3.24828E-11 | 2.80977E-05 | 22.90 | 30.46 | -7.56 | 0.62 | 0.50 | 0.74 |
| cg16207991 | PHACTR1 | 6p24.1 | 6.90981E-09 | 0.00597699 | 9.95 | 14.81 | -4.86 | 0.54 | 0.41 | 0.66 |
| cg24732404 | PLCB1 | 20p12.3 | 1.99834E-12 | 1.72856E-06 | 49.99 | 58.86 | -8.87 | 0.71 | 0.59 | 0.82 |
| cg26509964 | PLCE1 | 10q23.33 | 1.62472E-11 | 1.40538E-05 | 39.79 | 48.47 | -8.69 | 0.67 | 0.55 | 0.78 |
| cg13722084 | PRDM16 | 1p36.32 | 5.44487E-09 | 0.00470981 | 26.63 | 33.64 | -7.01 | 0.66 | 0.54 | 0.78 |
| cg16298405 | RUNX2 | 6p21.1 | 3.85196E-08 | 0.03331942 | 9.95 | 14.56 | -4.61 | 0.76 | 0.66 | 0.87 |
| cg06069187 | SARM1 | 17q11.2 | 1.99795E-12 | 1.72823E-06 | 20.87 | 28.62 | -7.75 | 0.66 | 0.54 | 0.78 |
| cg06422471 | SHANK3 | 22q13.33 | 4.44511E-08 | 0.03845017 | 20.88 | 15.91 | 4.98 | 0.66 | 0.54 | 0.78 |
| cg16293347 | TAOK3 | 12q24.23 | 2.43393E-09 | 0.00210535 | 79.30 | 73.63 | 5.66 | 0.72 | 0.61 | 0.84 |
| cg17546721 | TGFBR2 | 3p24.1 | 9.90267E-10 | 0.00085658 | 51.84 | 44.44 | 7.40 | 0.71 | 0.59 | 0.82 |
| cg22849544 | THRB | 3p24.2 | 1.15971E-08 | 0.01003149 | 18.73 | 24.80 | -6.07 | 0.57 | 0.45 | 0.69 |
| cg03037030 | TNF | 6p21.33 | 3.01487E-11 | 2.60787E-05 | 17.77 | 12.39 | 5.38 | 0.73 | 0.62 | 0.85 |
| cg17500968 | TRPV4 | 12q24.11 | 3.36784E-09 | 0.00291318 | 84.86 | 80.02 | 4.83 | 0.68 | 0.56 | 0.80 |
| cg26701226 | WSCD1 | 17p13.2 | 5.61785E-08 | 0.04859438 | 4.69 | 8.29 | -3.60 | 0.57 | 0.44 | 0.69 |

**Supplementary Table-S5**. Analysis of (+Opioids/+NOWS) versus (-Opioids/-NOWS, control), highlighting CpG targets showing significant methylation differences in genes associated with pain linked to NOWS.

| **Target ID** | **Gene** | **location** | **p-Val** | **FDR p-Val** | **% Methylation** | | | **AUC** | **CI** | |
| --- | --- | --- | --- | --- | --- | --- | --- | --- | --- | --- |
|  |  |  |  |  | **Cases** | **Control** | **change** |  | **lower** | **upper** |
| cg02237342 | ADORA2A | 22q11.23 | 5.13639E-08 | 0.044429736 | 89.37 | 92.97 | -3.61 | 0.77 | 0.66 | 0.89 |
| cg19621317 | ASIC1 | 12q13.12 | 2.6291E-08 | 0.022741751 | 71.62 | 78.17 | -6.56 | 0.80 | 0.70 | 0.91 |
| cg11211173 | ATP1A2 | 1q23.2 | 1.47109E-10 | 0.00012725 | 75.90 | 68.59 | 7.31 | 0.74 | 0.61 | 0.86 |
| cg15442907 | CACNA1C | 12p13.33 | 1.86658E-08 | 0.016145948 | 45.52 | 54.02 | -8.50 | 0.72 | 0.60 | 0.85 |
| cg08929188 | CALCA | 11p15.2 | 2.00477E-13 | 1.73412E-07 | 25.95 | 17.92 | 8.02 | 0.75 | 0.63 | 0.87 |
| cg19690051 | CAPN1 | 11q13.1 | 8.09803E-10 | 0.000700479 | 15.48 | 9.98 | 5.51 | 0.89 | 0.80 | 0.97 |
| cg23817893 | CCDC81 | 11q14.2 | 3.29234E-09 | 0.002847878 | 42.62 | 51.54 | -8.92 | 0.73 | 0.61 | 0.85 |
| cg26635219 | CFTR | 7q31.2 | 9.66969E-39 | 8.36428E-33 | 24.59 | 14.19 | 10.40 | 0.76 | 0.64 | 0.88 |
| cg12078872 | DDO | 6q21 | 1.33095E-08 | 0.011512755 | 80.37 | 85.75 | -5.38 | 0.80 | 0.70 | 0.91 |
| cg14972143 | EIF4E | 4q23 | 4.70268E-09 | 0.004067822 | 16.56 | 11.10 | 5.46 | 0.84 | 0.74 | 0.94 |
| cg20583095 | ESR1 | 6q25.1-q25.2 | 1.8417E-09 | 0.001593074 | 34.11 | 26.51 | 7.61 | 0.77 | 0.65 | 0.88 |
| cg01015652 | ESR2 | 14q23.2-q23.3 | 4.44223E-10 | 0.000384253 | 16.52 | 10.78 | 5.74 | 0.78 | 0.66 | 0.89 |
| cg21685789 | GABRG2 | 5q34 | 3.2526E-09 | 0.002813495 | 59.23 | 51.15 | 8.09 | 0.70 | 0.57 | 0.82 |
| cg00248439 | GRK5 | 10q26.11 | 8.52887E-11 | 7.37748E-05 | 89.72 | 84.80 | 4.93 | 0.70 | 0.57 | 0.83 |
| cg15002761 | IGSF9B | 11q25 | 1.14155E-08 | 0.009874417 | 87.39 | 91.52 | -4.14 | 0.76 | 0.64 | 0.88 |
| cg20629735 | IL18R1 | 2q12.1 | 4.15213E-08 | 0.035915968 | 75.09 | 81.10 | -6.01 | 0.71 | 0.59 | 0.84 |
| cg13999099 | IL6ST | 5q11.2 | 2.40982E-38 | 2.0845E-32 | 26.41 | 16.95 | 9.46 | 0.92 | 0.85 | 0.99 |
| cg13377102 | KCNAB3 | 17p13.1 | 6.62586E-14 | 5.73137E-08 | 70.71 | 79.30 | -8.59 | 0.80 | 0.69 | 0.91 |
| cg19137569 | KCNN3 | 1q21.3 | 3.51656E-08 | 0.030418261 | 32.61 | 40.65 | -8.04 | 0.78 | 0.66 | 0.89 |
| cg03786924 | KCNQ5 | 6q13 | 4.94018E-08 | 0.04273256 | 29.22 | 22.61 | 6.61 | 0.74 | 0.62 | 0.86 |
| cg21176263 | LMX1B | 9q33.3 | 2.65269E-09 | 0.002294578 | 64.92 | 72.69 | -7.77 | 0.75 | 0.62 | 0.87 |
| cg05313261 | MAPK3 | 16p11.2 | 5.72163E-10 | 0.000494921 | 6.60 | 11.74 | -5.14 | 0.88 | 0.80 | 0.97 |
| cg24368031 | MRC2 | 17q23.2 | 1.7516E-10 | 0.000151514 | 28.42 | 20.97 | 7.45 | 0.75 | 0.63 | 0.87 |
| cg21517792 | MTA1 | 14q32.33 | 5.79733E-15 | 5.01469E-09 | 78.15 | 85.62 | -7.46 | 0.82 | 0.71 | 0.92 |
| cg02726883 | NF1 | 17q11.2 | 2.37431E-08 | 0.020537772 | 14.59 | 9.63 | 4.96 | 0.91 | 0.84 | 0.99 |
| cg25067242 | NGF | 1p13.2 | 5.88023E-09 | 0.0050864 | 48.57 | 57.28 | -8.72 | 0.77 | 0.65 | 0.88 |
| cg17369032 | NGFR | 17q21.33 | 5.50827E-09 | 0.004764656 | 75.13 | 81.46 | -6.33 | 0.77 | 0.65 | 0.89 |
| cg02904605 | NOTCH3 | 19p13.12 | 6.00623E-17 | 5.19539E-11 | 64.42 | 74.86 | -10.43 | 0.78 | 0.67 | 0.89 |
| cg02111786 | NRXN3 | 14q24.3-q31.1 | 9.6026E-09 | 0.008306249 | 85.00 | 79.74 | 5.26 | 0.73 | 0.60 | 0.85 |
| cg27576691 | OXR1 | 8q23.1 | 1.836E-08 | 0.015881367 | 70.75 | 77.49 | -6.73 | 0.70 | 0.58 | 0.83 |
| cg24732404 | PLCB1 | 20p12.3 | 4.79624E-13 | 4.14875E-07 | 48.26 | 58.86 | -10.60 | 0.72 | 0.60 | 0.85 |
| cg26509964 | PLCE1 | 10q23.33 | 5.30528E-13 | 4.58907E-07 | 37.85 | 48.47 | -10.62 | 0.71 | 0.59 | 0.84 |
| cg27331241 | PRKAR1B | 7p22.3 | 1.77457E-11 | 1.535E-05 | 52.56 | 62.30 | -9.74 | 0.71 | 0.59 | 0.84 |
| cg00781169 | PTGER3 | 1p31.1 | 1.76058E-08 | 0.015229 | 60.07 | 67.91 | -7.85 | 0.70 | 0.57 | 0.83 |
| cg09070522 | REST | 4q12 | 1.91443E-08 | 0.01655985 | 14.44 | 9.48 | 4.96 | 0.88 | 0.79 | 0.97 |
| cg21486834 | RHBDF2 | 17q25.1 | 8.80873E-09 | 0.007619551 | 81.54 | 86.77 | -5.23 | 0.76 | 0.64 | 0.87 |
| cg06422471 | SHANK3 | 22q13.33 | 5.69456E-08 | 0.049257958 | 21.70 | 15.91 | 5.80 | 0.71 | 0.58 | 0.83 |
| cg10439765 | SLC12A5 | 20q13.12 | 2.61284E-08 | 0.022601057 | 15.53 | 21.81 | -6.27 | 0.70 | 0.57 | 0.82 |
| cg24397382 | STX1A | 7q11.23 | 1.1021E-08 | 0.009533133 | 71.48 | 64.43 | 7.05 | 0.70 | 0.57 | 0.83 |
| cg03037030 | TNF | 6p21.33 | 1.06467E-08 | 0.00920941 | 17.95 | 12.39 | 5.55 | 0.73 | 0.60 | 0.85 |

**Supplementary Table-S6.** Comparison between (+Opioids/-NOWS) and (-Opioids/-NOWS, control) elucidates CpG targets that exhibit significant differential methylation in genes linked to pain in NOWS.

| **TargetID** | **Gene** | **location** | **p-Val** | **FDR p-Val** | **% Methylation** | | | **AUC** | **CI** | |
| --- | --- | --- | --- | --- | --- | --- | --- | --- | --- | --- |
|  |  |  |  |  | **Cases** | **Control** | **change** |  | **lower** | **upper** |
| cg22018329 | GNA11 | 19p13.3 | 1.03032E-37 | 8.91224E-32 | 85.92 | 78.79 | 7.13 | 0.77 | 0.65 | 0.88 |
| cg07444152 | EHMT2 | 6p21.33 | 1.06967E-14 | 9.25264E-09 | 77.56 | 69.17 | 8.39 | 0.77 | 0.65 | 0.88 |
| cg14117934 | IL1B | 2q14.1 | 1.07108E-14 | 9.26487E-09 | 77.75 | 69.36 | 8.39 | 0.81 | 0.70 | 0.91 |
| cg15677797 | KLF11 | 2p25.1 | 3.22264E-14 | 2.78758E-08 | 6.89 | 13.28 | -6.39 | 0.57 | 0.43 | 0.72 |
| cg15929698 | NPY | 7p15.3 | 1.25316E-13 | 1.08398E-07 | 50.16 | 40.25 | 9.90 | 0.75 | 0.63 | 0.87 |
| cg18354203 | BDNF | 11p14.1 | 3.63971E-13 | 3.14835E-07 | 79.02 | 71.31 | 7.71 | 0.81 | 0.70 | 0.91 |
| cg12056044 | IL23R | 1p31.3 | 4.49673E-12 | 3.88967E-06 | 71.28 | 62.90 | 8.39 | 0.72 | 0.60 | 0.85 |
| cg25885356 | RUNX1 | 21q22.12 | 1.18691E-11 | 1.02668E-05 | 44.32 | 35.28 | 9.04 | 0.72 | 0.59 | 0.85 |
| cg17546721 | TGFBR2 | 3p24.1 | 4.99923E-11 | 4.32434E-05 | 53.38 | 44.44 | 8.95 | 0.75 | 0.63 | 0.87 |
| cg16293347 | TAOK3 | 12q24.23 | 1.15013E-10 | 9.94861E-05 | 80.32 | 73.63 | 6.68 | 0.77 | 0.65 | 0.88 |
| cg17782167 | PLCE1 | 10q23.33 | 1.19517E-10 | 0.000103382 | 63.77 | 55.31 | 8.46 | 0.75 | 0.63 | 0.87 |
| cg15118537 | PRKG1 | 10q11.23-q21.1 | 1.3056E-10 | 0.000112934 | 72.65 | 64.95 | 7.69 | 0.73 | 0.60 | 0.85 |
| cg25387779 | GNAO1 | 16q13 | 4.10377E-10 | 0.000354976 | 18.94 | 26.52 | -7.58 | 0.65 | 0.51 | 0.78 |
| cg14299235 | ABCA1 | 9q31.1 | 5.50932E-10 | 0.000476556 | 67.75 | 59.84 | 7.91 | 0.68 | 0.55 | 0.81 |
| cg11211173 | ATP1A2 | 1q23.2 | 9.90205E-10 | 0.000856527 | 75.60 | 68.59 | 7.01 | 0.72 | 0.59 | 0.84 |
| cg09397542 | PHACTR1 | 6p24.1 | 1.06673E-09 | 0.000922725 | 9.63 | 15.34 | -5.71 | 0.70 | 0.58 | 0.83 |
| cg07616332 | SHMT1 | 17p11.2 | 1.35291E-09 | 0.001170264 | 63.11 | 55.05 | 8.06 | 0.71 | 0.59 | 0.84 |
| cg12602112 | EDNRB | 13q22.3 | 1.40547E-09 | 0.00121573 | 11.09 | 17.06 | -5.97 | 0.70 | 0.58 | 0.83 |
| cg04329125 | CRIP2 | [14q32.33](https://www.omim.org/geneMap/14/596?start=-3&limit=10&highlight=596) | 2.04734E-09 | 0.001770948 | 47.33 | 39.10 | 8.23 | 0.70 | 0.58 | 0.83 |
| cg12950817 | CACNA1C | 12p13.33 | 2.56837E-09 | 0.002221644 | 78.44 | 71.96 | 6.48 | 0.73 | 0.61 | 0.86 |
| cg10274696 | C7orf10 | [7p14.1](https://www.omim.org/geneMap/7/204?start=-3&limit=10&highlight=204) | 2.78213E-09 | 0.002406545 | 74.63 | 67.68 | 6.95 | 0.69 | 0.56 | 0.82 |
| cg09021274 | DLG2 | 11q14.1 | 2.9843E-09 | 0.002581422 | 84.55 | 79.06 | 5.50 | 0.86 | 0.77 | 0.95 |
| cg09964361 | CAMK4 | 5q22.1 | 3.42272E-09 | 0.002960655 | 63.94 | 56.11 | 7.83 | 0.71 | 0.58 | 0.84 |
| cg25944168 | EIF2AK3 | 2p11.2 | 6.28034E-09 | 0.005432496 | 63.88 | 56.16 | 7.72 | 0.71 | 0.58 | 0.84 |
| cg27625200 | CLIC4 | 1p36.11 | 6.48624E-09 | 0.005610595 | 72.27 | 65.21 | 7.05 | 0.74 | 0.62 | 0.87 |
| cg00263307 | NF1 | 17q11.2 | 7.66598E-09 | 0.006631071 | 80.82 | 74.86 | 5.96 | 0.72 | 0.60 | 0.85 |
| cg13377102 | KCNAB3 | 17p13.1 | 8.73136E-09 | 0.007552628 | 72.72 | 79.30 | -6.58 | 0.71 | 0.58 | 0.83 |
| cg11590170 | GJA1 | 6q22.31 | 1.02546E-08 | 0.008870208 | 79.56 | 73.47 | 6.09 | 0.74 | 0.61 | 0.86 |
| cg23088672 | ULK1 | 12q24.33 | 1.16506E-08 | 0.010077787 | 63.61 | 55.99 | 7.61 | 0.70 | 0.57 | 0.83 |
| ch.12.28033R | WNK1 | 12p13.33 | 1.47907E-08 | 0.012793967 | 11.78 | 17.49 | -5.71 | 0.61 | 0.48 | 0.75 |
| cg09713515 | DOCK4 | 7q31.1 | 1.55798E-08 | 0.013476549 | 81.31 | 75.54 | 5.77 | 0.76 | 0.65 | 0.88 |
| cg24617390 | CAPN1 | 11q13.1 | 1.97547E-08 | 0.017087832 | 76.11 | 69.68 | 6.43 | 0.79 | 0.68 | 0.91 |
| cg07539983 | SPARC | 5q33.1 | 2.17463E-08 | 0.018810541 | 79.24 | 73.23 | 6.01 | 0.74 | 0.61 | 0.86 |
| cg04708753 | CASP9 | 1p36.21 | 2.59019E-08 | 0.022405125 | 6.54 | 11.12 | -4.57 | 0.60 | 0.46 | 0.74 |
| cg18793036 | MME | 3q25.2 | 3.01615E-08 | 0.026089656 | 14.89 | 21.02 | -6.12 | 0.65 | 0.52 | 0.79 |
| cg21295398 | BECN1 | 17q21.31 | 3.3337E-08 | 0.028836492 | 74.35 | 67.82 | 6.53 | 0.83 | 0.73 | 0.94 |
| cg08914905 | PIK3C3 | 18q12.3 | 3.43041E-08 | 0.029673043 | 75.40 | 68.99 | 6.41 | 0.71 | 0.58 | 0.84 |
| cg22762326 | OXR1 | 8q23.1 | 3.589E-08 | 0.031044881 | 82.45 | 76.99 | 5.46 | 0.75 | 0.62 | 0.87 |
| cg03037030 | TNF | 6p21.33 | 3.94761E-08 | 0.034146844 | 17.72 | 12.39 | 5.33 | 0.74 | 0.62 | 0.86 |
| cg25065716 | ADARB2 | 10p15.3 | 3.96823E-08 | 0.034325203 | 23.22 | 30.42 | -7.20 | 0.69 | 0.56 | 0.82 |
| cg06069187 | SARM1 | 17q11.2 | 4.14952E-08 | 0.035893387 | 21.62 | 28.62 | -7.00 | 0.67 | 0.54 | 0.80 |
| cg22623236 | PDE10A | 6q27 | 4.44015E-08 | 0.038407326 | 73.88 | 67.35 | 6.53 | 0.67 | 0.54 | 0.80 |
| cg05337454 | NOS3 | 7q36.1 | 4.58437E-08 | 0.039654829 | 88.15 | 83.70 | 4.45 | 0.70 | 0.57 | 0.83 |
| cg14614793 | IKBKAP | [9q31.3](https://www.omim.org/geneMap/9/392?start=-3&limit=10&highlight=392) | 4.61536E-08 | 0.039922855 | 11.28 | 16.70 | -5.42 | 0.69 | 0.56 | 0.82 |
| cg15153756 | GRK5 | 10q26.11 | 4.96835E-08 | 0.042976266 | 18.04 | 24.54 | -6.50 | 0.70 | 0.57 | 0.83 |
| cg17403731 | HCN2 | 19p13.3 | 5.11582E-08 | 0.044251849 | 67.65 | 60.59 | 7.05 | 0.70 | 0.57 | 0.83 |
| cg08238791 | RUNX2 | 6p21.1 | 5.27211E-08 | 0.045603772 | 9.33 | 14.37 | -5.03 | 0.71 | 0.58 | 0.84 |
| cg25015598 | CTSS | 1q21.3 | 5.70839E-08 | 0.049377568 | 61.41 | 54.01 | 7.40 | 0.72 | 0.59 | 0.84 |
| cg03349819 | NRG1 | 8p12 | 5.72285E-08 | 0.049502615 | 18.79 | 25.36 | -6.57 | 0.68 | 0.55 | 0.81 |
|  |  |  |  |  |  |  |  |  |  |  |
|  |  |  |  |  |  |  |  |  |  |  |
|  |  |  |  |  |  |  |  |  |  |  |
