# Supplementary material for: Prenatal opioid exposure alters pain perception and increases long-term health risks in infants with neonatal opioid withdrawal syndrome
Source: Front Pain Res (Lausanne). 2025 Apr 17;6:1497801. doi: 10.3389/fpain.2025.1497801 (PMC12043715; doi:10.3389/fpain.2025.1497801)
Supplement: Supplementary file 9 [file Table5.docx]

**Supplementary Table-S7.** Biological processes (BP) enrichment analysis**.**

| **Term** | **Genes** | **PValue2** | **FDR** |
| --- | --- | --- | --- |
| GO:0007204~positive regulation of cytosolic calcium ion concentration | *PTGIR, CALCA, EDNRB, GALR1, TRPV4, PTGER3, LPAR1, CACNA1C, OPRM1, ESR1, PRKG1* | 6.72192E-09 | 1.02576E-05 |
| GO:0098703~calcium ion import across plasma membrane | *CACNA2D1, TRPV4, SCN8A, CACNA1C, CACNA1H, SCN1A* | 1.90519E-06 | 0.001453657 |
| GO:0042311~vasodilation | *CALCA, EDNRB, ADORA2A, NOS3, TNF, ATG5* | 4.01648E-06 | 0.00204305 |
| GO:0006468~protein phosphorylation | *MAPK10, CALCA, TAOK3, GRK5, PRKAR1B, WNK1, CAMK4, EIF2AK3, ULK1, PRKG1, TGFBR2, MAPK3* | 7.07822E-06 | 0.002488862 |
| GO:0086002~cardiac muscle cell action potential involved in contraction | *CACNA2D1, SCN8A, CACNA1C, KCNJ2, SCN1A* | 8.15485E-06 | 0.002488862 |
| GO:0007422~peripheral nervous system development | *EDNRB, BDNF, SCN8A, NF1, NGF* | 1.33002E-05 | 0.003091604 |
| GO:0007200~phospholipase C-activating G-protein coupled receptor signaling pathway | *CALCA, EDNRB, PTGER3, LPAR1, PLCE1, OPRM1, ESR1* | 1.41817E-05 | 0.003091604 |
| GO:0086012~membrane depolarization during cardiac muscle cell action potential | *ATP1A2, CACNA1C, HCN2, KCNJ2* | 3.34429E-05 | 0.00637924 |
| GO:0007165~signal transduction | *GRIA1, CXCL8, NRXN3, NR3C1, ESR1, ESR2, MAPK10, GJA1, MTA1, PDE10A, GRK5, WNK1, IL1B, CAMK4, GNA11, SARM1, ULK1, IL18R1, PRKG1, ASIC1* | 4.17184E-05 | 0.007073593 |
| GO:0008217~regulation of blood pressure | *CALCA, EDNRB, NOS3, NPY, GNA11, ATP1A2* | 6.74161E-05 | 0.010287691 |
| GO:0007189~adenylate cyclase-activating G-protein coupled receptor signaling pathway | *ABCA1, PTGIR, CALCA, ADORA2A, GALR1, PTGER3, LPAR1* | 8.19789E-05 | 0.011372711 |
| GO:0008284~positive regulation of cell proliferation | *NLGN2, EDNRB, GRK5, IL1B, OSM, CRIP2, NRG1, PTN, CAPN1, IL6ST, PRLR, TGFBR2* | 9.611E-05 | 0.012221991 |
| GO:0008285~negative regulation of cell proliferation | *BECN1, KLF11, REST, CXCL8, ADORA2A, NOS3, IL1B, OSM, ULK1, OPRM1, NGF* | 0.000123397 | 0.014484962 |
| GO:0016310~phosphorylation | *ULK4, EIF2AK3, TGFBR2, MAPK10, PRKAR1B, TAOK3, GRK5, WNK1, CAMK4, PIK3C3, ANKK1, PRKG1, MAPK3* | 0.000148581 | 0.01619534 |
| GO:0021675~nerve development | *NGFR, BDNF, NGF, SCN1A* | 0.000167878 | 0.017078767 |
| GO:1900273~positive regulation of long-term synaptic potentiation | *ADORA2A, PRKAR1B, MME, SHANK3* | 0.000257716 | 0.024579641 |
| GO:0007267~cell-cell signaling | *GJA1, PTGIR, CALCA, ADORA2A, IL1B, PCSK5, HCN2, ESR2* | 0.000274317 | 0.024623985 |
| GO:0031622~positive regulation of fever generation | *IL1B, PTGER3, TNF* | 0.00029138 | 0.024702569 |
| GO:0035725~sodium ion transmembrane transport | *WNK1, SCN8A, ATP1A2, HCN2, SCN1A, ASIC1* | 0.00043491 | 0.034930155 |
| GO:0006954~inflammatory response | *PTGIR, CALCA, CXCL8, ADORA2A, IL1B, IL23R, CAMK4, PTGER3, TNF, IL18R1* | 0.000461842 | 0.035238558 |

**Supplementary Table-S8.** Cellular Component (CC) enrichment analysis**.**

| **Term** | **Genes** | **P-Value** | **FDR** |
| --- | --- | --- | --- |
| GO:0005886~plasma membrane | *DOCK4, CLIC4, SPARC, IL23R, TNF, GJA1, EDNRB, PLCE1, CAPN1, PRKG1, SCN1A, PTGIR, MME, CACNA2D1, RHBDF2, NRG1, OPRM1, KCNAB3, PRLR, GABRG2, TGFBR2, AJAP1, ADORA2A, PRKAR1B, SCN8A, KCNQ5, IL6ST, SHANK3, STX1A, CFTR, GRIA1, NOTCH3, NLGN2, PTGER3, NRXN3, LPAR1, ATP1A2, PTN, CACNA1C, CACNA1H, GRK5, ANKH, GALR1, GNA11, KCNN3, KCNJ2, ASIC1, MAPK3, ABCA1, NGFR, SLC12A5, NOS3, ESR1, GNAO1, MAPK10, DLG2, TAOK3, TRPV4, NF1, HCN2, IL18R1* | 3.71713E-11 | 7.47143E-09 |
| GO:0030424~axon | *MME, BDNF, SCN8A, SARM1, NF1, ULK1, OPRM1, NGF, HCN2, STX1A, GABRG2, ATG5* | 2.16191E-06 | 0.000194619 |
| GO:0043025~neuronal cell body | *GRIA1, CALCA, SLC12A5, ADORA2A, MME, LPAR1, ATP1A2, KCNN3, IL6ST, TNF, KCNJ2, SCN1A* | 2.90477E-06 | 0.000194619 |
| GO:0098978~glutamatergic synapse | *GRIA1, ADORA2A, PRKAR1B, SCN8A, NRXN3, NF1, LPAR1, NRG1, EIF4E, SHANK3, KCNJ2, MAPK3* | 1.00718E-05 | 0.000506107 |
| GO:0030425~dendrite | *GRIA1, BECN1, ADORA2A, MME, BDNF, SARM1, NF1, CACNA1C, OPRM1, NGF, IL6ST, HCN2* | 1.77255E-05 | 0.000712565 |
| GO:0009986~cell surface | *GRIA1, NGFR, NOTCH3, CLIC4, NLGN2, SPARC, MME, LPAR1, PRLR, TNF, AJAP1, TRPV4, CFTR, ASIC1* | 2.77593E-05 | 0.000929937 |
| GO:0005891~voltage-gated calcium channel complex | *CACNA2D1, SCN8A, CACNA1C, CACNA1H, SCN1A* | 3.89531E-05 | 0.00111851 |
| GO:0016020~membrane | *DOCK4, CLIC4, SPARC, IL23R, NR3C1, PCSK5, MRC2, CAPN1, PTGIR, MME, CACNA2D1, RHBDF2, NRG1, OPRM1, GABRG2, TGFBR2, ADORA2A, SCN8A, KCNQ5, PIK3C3, IL6ST, STX1A, CFTR, GRIA1, NLGN2, PTGER3, ATP1A2, CACNA1C, CACNA1H, ANKH, GALR1, GNA11, KCNN3, KCNJ2, ATG5, ASIC1, ABCA1, NGFR, SLC12A5, BDNF, EIF2AK3, ESR1, GNAO1, DLG2, WNK1, TRPV4, NF1, IGSF9B, IL18R1* | 5.13386E-05 | 0.001289883 |
| GO:0045211~postsynaptic membrane | *GRIA1, NLGN2, ADORA2A, LPAR1, IGSF9B, SHANK3, GABRG2, KCNJ2* | 8.37241E-05 | 0.001774456 |
| GO:0008076~voltage-gated potassium channel complex | *DLG2, KCNQ5, KCNAB3, HCN2, STX1A, KCNJ2* | 8.82814E-05 | 0.001774456 |
| GO:0043005~neuron projection | *SLC12A5, DLG2, IGSF9B, KCNN3, OPRM1, SHANK3, STX1A, GABRG2* | 0.001099793 | 0.020096213 |
| GO:0005737~cytoplasm | *BECN1, DOCK4, CLIC4, CALCA, SPARC, CPQ, SHMT1, LPAR1, ATP1A2, CACNA1C, NR3C1, ADARB2, DDO, CASP9, GJA1, MTA1, GRK5, GNA11, KCNN3, CAPN1, EIF4E, PRKG1, ATG5, MAPK3, MME, NOS3, BDNF, EIF2AK3, KCNAB3, ESR1, RUNX2, GNAO1, MAPK10, REST, TAOK3, WNK1, CAMK4, NF1, SARM1, ULK1, PIK3C3, ANKK1, CCDC81, SHANK3, CFTR* | 0.00122784 | 0.020336207 |
| GO:0030315~T-tubule | *ATP1A2, CACNA1C, KCNJ2, SCN1A* | 0.001328347 | 0.020336207 |
| GO:0043197~dendritic spine | *GRIA1, NGFR, LPAR1, ATP1A2, SHANK3, KCNJ2* | 0.001416452 | 0.020336207 |
| GO:0014704~intercalated disc | *GJA1, ATP1A2, KCNJ2, SCN1A* | 0.00195693 | 0.026222861 |
| GO:0005829~cytosol | *BECN1, NOTCH3, DOCK4, CLIC4, SHMT1, NR3C1, DDO, CASP9, GJA1, MTA1, GRK5, PRDM16, PLCE1, PHACTR1, CAPN1, EIF4E, N4BP1, PRKG1, ATG5, MAPK3, NGFR, KLF11, PTGIR, NOS3, EIF2AK3, NGF, ESR1, RUNX2, TGFBR2, MAPK10, REST, DLG2, PDE10A, PRKAR1B, WNK1, IL1B, NF1, SARM1, ULK1, PIK3C3, SHANK3, STX1A, CFTR* | 0.003089937 | 0.03881733 |

**Supplementary Table-S9.** Molecular function (MF) enrichment analysis.

| **Term** | **Genes** | **P-Value** | **FDR** |
| --- | --- | --- | --- |
| GO:0097110~scaffold protein binding | *GRIA1, GJA1, NOS3, IL6ST, SHANK3, CACNA1H* | 4.38732E-05 | 0.015969851 |
| GO:0005516~calmodulin binding | *NGFR, ADORA2A, NOS3, TRPV4, CAMK4, KCNN3, CACNA1C, ESR1* | 0.000157816 | 0.028263388 |
| GO:0004672~protein kinase activity | *MAPK10, TAOK3, GRK5, WNK1, CAMK4, ULK4, EIF2AK3, PIK3C3, ANKK1, PRKG1* | 0.000297556 | 0.028263388 |
| GO:0008331~high voltage-gated calcium channel activity | *SCN8A, CACNA1C, CACNA1H, SCN1A* | 0.000310587 | 0.028263388 |
| GO:0042802~identical protein binding | *GRIA1, BECN1, NOTCH3, CALCA, NLGN2, SHMT1, EIF2AK3, NR3C1, ESR1, TNF, CASP9, REST, ADORA2A, TRPV4, SARM1, ULK1, IL6ST, HCN2, KCNJ2, STX1A, PRKG1, MAPK3* | 0.000501536 | 0.031621305 |
| GO:0005515~protein binding | *DOCK4, CLIC4, SPARC, THRB, CXCL8, EHMT2, IL23R, NR3C1, PCSK5, TNF, MRC2, CASP9, GJA1, EDNRB, PLCE1, PHACTR1, CAPN1, PRKG1, KLF11, MME, RHBDF2, OPRM1, NGF, KCNAB3, PRLR, GABRG2, RUNX2, TGFBR2, AJAP1, RUNX1, ADORA2A, PRKAR1B, SCN8A, IL1B, KCNQ5, PIK3C3, ULK1, LMX1B, ANKK1, CCDC81, IL6ST, SHANK3, STX1A, CFTR, GRIA1, BECN1, NOTCH3, CALCA, SHMT1, LPAR1, ATP1A2, CRIP2, PTN, CACNA1C, DDO, CACNA1H, MTA1, GRK5, GALR1, GNA11, NPY, PRDM16, EIF4E, KCNJ2, N4BP1, ATG5, ASIC1, MAPK3, ABCA1, NGFR, NOS3, BDNF, OSM, EIF2AK3, ESR1, ESR2, GNAO1, MAPK10, REST, DLG2, TAOK3, WNK1, TRPV4, NF1, SARM1, HCN2, IL18R1* | 0.00060179 | 0.031621305 |
| GO:0005496~steroid binding | *ATP1A2, NR3C1, ESR1, ESR2* | 0.000608102 | 0.031621305 |
| GO:0005524~ATP binding | *ABCA1, ULK4, EIF2AK3, ATP1A2, RUNX2, TGFBR2, RUNX1, MAPK10, TAOK3, GRK5, WNK1, TRPV4, CAMK4, SCN8A, PIK3C3, ULK1, ANKK1, CFTR, PRKG1, MAPK3* | 0.000774543 | 0.035241708 |
| GO:0019899~enzyme binding | *NOTCH3, THRB, ADORA2A, EIF2AK3, PLCE1, ESR1, EIF4E, ESR2, CFTR* | 0.001153712 | 0.041768557 |
| GO:0005245~voltage-gated calcium channel activity | *CACNA2D1, CACNA1C, OPRM1, CACNA1H* | 0.001421361 | 0.041768557 |
| GO:0005261~cation channel activity | *TRPV4, SCN8A, CACNA1H, SCN1A* | 0.001421361 | 0.041768557 |
| GO:0004674~protein serine/threonine kinase activity | *TAOK3, GRK5, WNK1, CAMK4, ULK4, EIF2AK3, ULK1, ANKK1, MAPK3* | 0.001433079 | 0.041768557 |
| GO:0019901~protein kinase binding | *CASP9, GRIA1, BECN1, SLC12A5, WNK1, TRPV4, PTN, NR3C1, PRLR, ESR1* | 0.001491734 | 0.041768557 |
| GO:0034056~estrogen response element binding | *NR3C1, ESR1, ESR2* | 0.001630231 | 0.042386014 |

**Supplementary Table-S10.** Gene ontology enrichment analysis and KEGG pathway

| **Term** | **Genes** | **P-Value** | **FDR** |
| --- | --- | --- | --- |
| hsa04024:cAMP signaling pathway | *GRIA1, BDNF, PTGER3, ATP1A2, CACNA1C, MAPK10, PDE10A, ADORA2A, CAMK4, NPY, PLCE1, HCN2, CFTR, MAPK3* | 1.36406E-07 | 2.22342E-05 |
| hsa04010:MAPK signaling pathway | *NGFR, BDNF, CACNA2D1, CACNA1C, NGF, TNF, CACNA1H, TGFBR2, MAPK10, TAOK3, IL1B, NF1, MAPK3* | 2.05304E-05 | 0.001181253 |
| hsa04929:GnRH secretion | *GNA11, KCNN3, CACNA1C, CACNA1H, HCN2, ESR2, MAPK3* | 2.69842E-05 | 0.001181253 |
| hsa04080:Neuroactive ligand-receptor interaction | *GRIA1, PTGIR, CALCA, THRB, PTGER3, LPAR1, OPRM1, NR3C1, PRLR, GABRG2, EDNRB, ADORA2A, NPY, GALR1* | 3.14088E-05 | 0.001181253 |
| hsa04933:AGE-RAGE signaling pathway in diabetic complications | *MAPK10, CXCL8, NOS3, IL1B, PLCE1, TNF, TGFBR2, MAPK3* | 3.82598E-05 | 0.001181253 |
| hsa05142:Chagas disease | *MAPK10, GNAO1, CXCL8, IL1B, GNA11, TNF, TGFBR2, MAPK3* | 4.34817E-05 | 0.001181253 |
| hsa04210:Apoptosis | *MAPK10, CASP9, EIF2AK3, CAPN1, NGF, TNF, CTSS, MAPK3* | 0.000266425 | 0.006203905 |
| hsa05200:Pathways in cancer | *NOTCH3, CXCL8, IL23R, PTGER3, LPAR1, ESR1, ESR2, TGFBR2, RUNX1, MAPK10, CASP9, EDNRB, GNA11, IL6ST, MAPK3* | 0.000359633 | 0.006750825 |
| hsa04060:Cytokine-cytokine receptor interaction | *NGFR, CXCL8, IL1B, IL23R, OSM, NGF, IL6ST, PRLR, TNF, IL18R1, TGFBR2* | 0.000419716 | 0.006750825 |
| hsa05022:Pathways of neurodegeneration - multiple diseases | *GRIA1, BECN1, BDNF, EIF2AK3, CACNA1C, TNF, MAPK10, CASP9, IL1B, PIK3C3, ULK1, CAPN1, STX1A, MAPK3* | 0.00042788 | 0.006750825 |
| hsa05131:Shigellosis | *MAPK10, BECN1, CXCL8, IL1B, PLCE1, PIK3C3, CAPN1, TNF, ATG5, MAPK3* | 0.000469019 | 0.006750825 |
| hsa04659:Th17 cell differentiation | *MAPK10, IL1B, IL23R, IL6ST, TGFBR2, MAPK3, RUNX1* | 0.000496993 | 0.006750825 |
| hsa04020:Calcium signaling pathway | *EDNRB, ADORA2A, NOS3, CAMK4, GNA11, PTGER3, PLCE1, CACNA1C, NGF, CACNA1H* | 0.000558324 | 0.00700053 |
| hsa04725:Cholinergic synapse | *GNAO1, CAMK4, GNA11, KCNQ5, CACNA1C, KCNJ2, MAPK3* | 0.00063237 | 0.007362593 |
| hsa05010:Alzheimer disease | *MAPK10, CASP9, BECN1, MME, IL1B, EIF2AK3, ULK1, PIK3C3, CACNA1C, CAPN1, TNF, MAPK3* | 0.000834142 | 0.008693455 |
| hsa05417:Lipid and atherosclerosis | *MAPK10, ABCA1, CASP9, CXCL8, NOS3, IL1B, EIF2AK3, TNF, MAPK3* | 0.000858234 | 0.008693455 |
| hsa04919:Thyroid hormone signaling pathway | *CASP9, NOTCH3, THRB, PLCE1, ATP1A2, ESR1, MAPK3* | 0.000906679 | 0.008693455 |
| hsa05152:Tuberculosis | *MAPK10, CASP9, MRC2, IL1B, PIK3C3, TNF, CTSS, MAPK3* | 0.001418439 | 0.012844752 |
| hsa04270:Vascular smooth muscle contraction | *PTGIR, CALCA, ADORA2A, GNA11, CACNA1C, PRKG1, MAPK3* | 0.001538281 | 0.013196834 |
| hsa04713:Circadian entrainment | *GNAO1, GRIA1, CACNA1C, CACNA1H, PRKG1, MAPK3* | 0.002078153 | 0.016936948 |
| hsa04730:Long-term depression | *GNAO1, GRIA1, GNA11, PRKG1, MAPK3* | 0.002325882 | 0.018053272 |
| hsa04921:Oxytocin signaling pathway | *GNAO1, NOS3, CACNA2D1, CAMK4, CACNA1C, KCNJ2, MAPK3* | 0.003104128 | 0.022777448 |
| hsa04215:Apoptosis - multiple species | *MAPK10, CASP9, NGFR, BECN1* | 0.003213996 | 0.022777448 |
| hsa04015:Rap1 signaling pathway | *GNAO1, NGFR, DOCK4, ADORA2A, LPAR1, PLCE1, NGF, MAPK3* | 0.003393964 | 0.02305067 |
| hsa04917:Prolactin signaling pathway | *MAPK10, PRLR, ESR1, ESR2, MAPK3* | 0.004075846 | 0.026574518 |
| hsa04140:Autophagy - animal | *MAPK10, BECN1, EIF2AK3, ULK1, PIK3C3, ATG5, MAPK3* | 0.004359355 | 0.027104269 |
| hsa04022:cGMP-PKG signaling pathway | *EDNRB, NOS3, GNA11, ATP1A2, CACNA1C, PRKG1, MAPK3* | 0.004489664 | 0.027104269 |
| hsa05163:Human cytomegalovirus infection | *GNAO1, CASP9, CXCL8, IL1B, GNA11, PTGER3, TNF, MAPK3* | 0.004953454 | 0.028227106 |
| hsa04722:Neurotrophin signaling pathway | *MAPK10, NGFR, BDNF, CAMK4, NGF, MAPK3* | 0.005022 | 0.028227106 |
| hsa05133:Pertussis | *MAPK10, CXCL8, IL1B, TNF, MAPK3* | 0.005467587 | 0.029707225 |
| hsa04151:PI3K-Akt signaling pathway | *CASP9, NGFR, BDNF, NOS3, OSM, LPAR1, NGF, PRLR, EIF4E, MAPK3* | 0.00611344 | 0.032144862 |
| hsa04926:Relaxin signaling pathway | *MAPK10, GNAO1, EDNRB, NOS3, TGFBR2, MAPK3* | 0.007040766 | 0.0358639 |
| hsa05016:Huntington disease | *MAPK10, CASP9, GRIA1, BECN1, REST, BDNF, ULK1, PIK3C3, STX1A* | 0.007594898 | 0.037514193 |
| hsa04911:Insulin secretion | *GNA11, ATP1A2, KCNN3, CACNA1C, STX1A* | 0.008436613 | 0.038046075 |
| hsa04380:Osteoclast differentiation | *MAPK10, IL1B, CAMK4, TNF, TGFBR2, MAPK3* | 0.008493266 | 0.038046075 |
| hsa04930:Type II diabetes mellitus | *MAPK10, CACNA1C, TNF, MAPK3* | 0.008957191 | 0.038046075 |
| hsa04915:Estrogen signaling pathway | *GNAO1, NOS3, OPRM1, ESR1, ESR2, MAPK3* | 0.00902044 | 0.038046075 |
| hsa04540:Gap junction | *GJA1, GNA11, LPAR1, PRKG1, MAPK3* | 0.009135398 | 0.038046075 |
| hsa05167:Kaposi sarcoma-associated herpesvirus infection | *MAPK10, CASP9, BECN1, CXCL8, PIK3C3, IL6ST, MAPK3* | 0.009436322 | 0.038046075 |
| hsa04211:Longevity regulating pathway | *EHMT2, CAMK4, ULK1, EIF4E, ATG5* | 0.009498546 | 0.038046075 |
| hsa04371:Apelin signaling pathway | *BECN1, NOTCH3, NOS3, CAMK4, PIK3C3, MAPK3* | 0.009569872 | 0.038046075 |
| hsa05032:Morphine addiction | *GNAO1, PDE10A, GRK5, OPRM1, GABRG2* | 0.010252809 | 0.039323541 |
| hsa05130:Pathogenic Escherichia coli infection | *MAPK10, CASP9, CXCL8, IL1B, LPAR1, TNF, MAPK3* | 0.010373695 | 0.039323541 |
| hsa04657:IL-17 signaling pathway | *MAPK10, CXCL8, IL1B, TNF, MAPK3* | 0.011455435 | 0.042437181 |
| hsa04723:Retrograde endocannabinoid signaling | *MAPK10, GNAO1, GRIA1, CACNA1C, GABRG2, MAPK3* | 0.012329428 | 0.044659928 |
| hsa01522:Endocrine resistance | *MAPK10, NOTCH3, ESR1, ESR2, MAPK3* | 0.013195399 | 0.044809376 |
| hsa04750:Inflammatory mediator regulation of TRP channels | *MAPK10, IL1B, TRPV4, NGF, ASIC1* | 0.013195399 | 0.044809376 |
| hsa04925:Aldosterone synthesis and secretion | *CAMK4, GNA11, ATP1A2, CACNA1C, CACNA1H* | 0.013195399 | 0.044809376 |

**Supplementary Table-S11.** Differentially Methylated Genes Identified Across Four Analyses and Their Overlap (Venn Diagram Representation).

| **Set Combination** | **Genes** |
| --- | --- |
| Analysis I | *AJAP1, ANKH, ARNTL, ATG5, CACNA2D1, CPQ, GRIA1, KCNJ2, LPAR1, MALAT1, N4BP1, MAPK10, NLGN2, NR3C1, OSM, PCSK5, PRLR, PTGIR, PTN, SCN1A, SCN8A, ULK4* |
| Analysis II | *ANKK1, CACNA1H, CXCL8, MYT1L, OPRM1, THRB, TRPV4, WSCD1* |
| Analysis III | *ADCYAP1, ASIC1, CALCA, DDO, EIF4E, ESR2, IGSF9B, KCNQ5, MAPK3,  NGFR, NRXN3, PRKAR1B, PTGER3, SLC12A5, STX1A* |
| Analysis IV | *ABCA1, CAMK4, CRIP2, DOCK4, EDNRB, EIF2AK3, GJA1, MME, NOS3, PIK3C3,  SHMT1, SPARC, WNK1* |
| Analysis I & Analysis II | *PRDM16* |
| Analysis I & Analysis III | *ADORA2A, CCDC81, GABRG2, IL18R1, MTA1, REST, RHBDF2* |
| Analysis I & Analysis IV | *BDNF, C7orf10, CLIC4, DLG2, IKBKAP, IL23R, NPY, PDE10A, PRKG1,  RUNX1, ULK1* |
| Analysis II & Analysis III | *ESR1, KCNN3, LMX1B, MRC2, NGF, NOTCH3, PLCB1, SHANK3* |
| Analysis II & Analysis IV | *BECN1, CASP9, GALR1, GNA11, GNAO1, HCN2, IL1B, KLF11, SARM1,  TAOK3, TGFBR2* |
| Analysis III & Analysis IV | *CAPN1, OXR1* |
| Analysis I & Analysis II & Analysis III | *CFTR, IL6ST* |
| Analysis I & Analysis II & Analysis IV | *ADARB2, CTSS, EHMT2, NRG1, PHACTR1, RUNX2* |
| Analysis II & Analysis III & Analysis IV | *ATP1A2, GRK5, KCNAB3, NF1, TNF* |
| Analysis I & Analysis III & Analysis IV | *CACNA1C* |
| Analysis I & Analysis II & Analysis III & Analysis IV | *PLCE1* |

**Analysis I: Comparison between +Opioids/+NOWS and +Opioids/-NOWS**

**Analysis II: Examination of (+Opioids/+NOWS), (+Opioids/-NOWS), and (-Opioids/-NOWS, control)**

**Analysis III: Comparison of (+Opioids/+NOWS) versus (-Opioids/-NOWS, control)**

**Analysis IV: Comparison between (+Opioids/-NOWS) and (-Opioids/-NOWS, control)**
