# Supplementary material for: Prenatal opioid exposure alters pain perception and increases long-term health risks in infants with neonatal opioid withdrawal syndrome
Source: Front Pain Res (Lausanne). 2025 Apr 17;6:1497801. doi: 10.3389/fpain.2025.1497801 (PMC12043715; doi:10.3389/fpain.2025.1497801)
Supplement: Supplementary file 10 [file Table6.docx]

**Prenatal opioid exposure alters pain perception and increases long-term health risks in infants with** **neonatal opioid withdrawal syndrome.**

**Running title:** Persistent pain and neonatal opioid withdrawal syndrome

Uppala Radhakrishna*^1,8^, Rupa Radhakrishnan^2^, Lavanya V Uppala^3^, Tithi S. Trivedi^4^

Jignesh Prajapati^5^, Rakesh M Rawal^6^, Srinivas B Muvvala^7^, Ray O Bahado-Singh^8,^ Senthilkumar Sadhasivam^1^.

_._

1. Department of Anesthesiology and Perioperative Medicine, University of Pittsburgh, Pittsburgh, PA, United States.
2. Department of Radiology and Imaging Sciences, Indiana University School of Medicine, Indianapolis, IN, United States.
3. School of Medicine, Creighton University, Omaha, NE, USA
4. Department of Botany, Bioinformatics and Climate Change Impacts Management, School of Sciences, Gujarat University, Ahmedabad, Gujarat, 380009, India
5. Department of Biochemistry & Forensic Sciences, Gujarat University, Ahmedabad – 380009
6. Department of Medical Biotechnology, Gujarat Biotechnology University, GIFT City, Gandhinagar-382355, Gujarat, India.
7. Department of Psychiatry, Yale School of Medicine, New Haven, CT, 06510, USA
8. Department of Obstetrics and Gynecology, Corewell Health William Beaumont University Hospital, Royal Oak, MI, USA.

**Correspondence:** Uppala Radhakrishna, Ph.D.

**Email:** [UPPALAR99@gmail.com](mailto:UPPALAR99@gmail.com)

**Abstract**

**Background**: Opioids are often prescribed for pain relief, yet they pose risks such as addiction, dependence, and overdose. Pregnant women have unique vulnerabilities to opioids and infants born to opioid-exposed mothers could develop neonatal opioid withdrawal syndrome (NOWS). The study of opioid-induced epigenetic changes in chronic pain is in its early stages. This study aimed to identify epigenetic changes in genes associated with chronic pain resulting from maternal opioid exposure during pregnancy.

**Methods**: We analyzed DNA methylation of chronic pain-related genes in 96 placental tissues using Illumina Infinium Methylation EPIC BeadChips. These samples comprised 32 from mothers with infants prenatally exposed to opioids who needed pharmacologic NOWS management (+Opioids/+NOWS), 32 from mothers with prenatally opioid-exposed infants not needing NOWS pharmacologic treatment (+Opioids/-NOWS)., and 32 from unexposed control subjects (-Opioids/-NOWS).

**Results**: The study identified significant methylation changes at 111 CpG sites in pain-related genes among opioid-exposed infants, with 54 CpGs hypomethylated and 57 hypermethylated. These genes play a crucial role in various biological processes, including telomere length regulation (*NOS3, ESR1, ESR2, MAPK3*); inflammation (*TNF, MAPK3, IL1B, IL23R*); glucose metabolism (*EIF2AK3, CACNA1H, NOTCH3, GJA1*); ion channel function (*CACNA1C, CACNA1H, CLIC4, KCNQ5*); autophagy (*CTSS, ULK1, ULK4, ATG5*); oxidative stress (*NGF, NRG1, OPRM1, ATP1A2*); aging (*GRIA1, NGFR, PRLR, EIF4E*); cytokine activity (*TRPV4, RUNX1, CXCL8, IL18R1*); and the risk of suicide (*ADORA2A, ANKK1, GABRG2, IGSF9B*). These epigenetic changes may influence 48 signaling pathways—including cAMP, MAPK, GnRH secretion, estrogen signaling, morphine addiction, circadian rhythms, and insulin secretion—profoundly affecting pain and inflammation-related processes.

**Conclusion**: The identified methylation alterations may shed light on pain, neurodevelopmental changes, and other biological mechanisms in opioid-exposed infants and mothers with OUD, offering insights into NOWS and maternal-infant health. These findings may also pave the way for targeted interventions and improved pain management, highlighting the potential for integrated care strategies to address the interconnected health of mothers and infants.

**Keywords:** Pain, biomarker, opioid use, epigenetics, DNA methylation, neonatal opioid withdrawal syndrome

**Introduction**

Chronic pain, also known as persistent pain, is a complex condition influenced by physical injuries, underlying medical conditions, and neurological issues (1). It can result from injuries, post-surgery complications, cancer, and pregnancy-related discomfort (2). Pain is categorized into nociceptive, neuropathic, and hyperalgesia types. Nociceptive pain stems from tissue damage like injuries or inflammation, felt as sharp or aching, that persists longer than the normal healing time (3). Neuropathic pain, frequently persistent, emerges from nerve system damage, characterized by enduring burning sensations observed in conditions like diabetic neuropathy lasting beyond 3 months. Hyperalgesia, marked by heightened pain sensitivity to even mild stimuli, can occur locally or globally due to factors like injury, inflammation, or certain neurological conditions (4-6). Pain management uses diverse medications and therapies, both prescription and nonprescription, to ease severe pain (7). Opioids are recognized for their pain-relieving properties and euphoric effects, making them susceptible to misuse (8).

Prolonged opioid use during pregnancy can induce genetic and epigenetic alterations in both mother and fetus, increasing addiction vulnerability and impacting neurodevelopment (9-13). These changes may contribute to birth defects, miscarriage, premature birth, low birth weight, respiratory and feeding issues, maternal health risks, NOWS, and future behavioral challenges (14-16).

The relationship between epigenetic dysregulation and persistent pain is an evolving research domain, with ongoing studies enhancing our understanding of its impact on pain tolerance. (17). Recent studies have highlighted the role of epigenetic changes in chronic pain (18), particularly through DNA methylation, which influences genes regulating pain signaling, inflammation, and stress responses (19). The relationship between chronic pain and DNA methylation is bidirectional. Pain can induce DNA methylation changes that affect pain sensitivity, creating a complex feedback loop that perpetuates chronic pain states (20). Opioids alleviate pain by acting on the central nervous system, rather than directly influencing pain-related genes (21). Chronic pain is influenced by a complex interplay of factors, including inflammation, nerve sensitization, changes in the central nervous system, genetics, epigenetics, environmental influences, and gender differences (22, 23). These factors can significantly impact pain perception, tolerance, and the effectiveness of pain management strategies, as well as the side effects experienced by individuals.

This study aims to identify key gene networks and epigenetic changes associated with opioid-associated pain dysregulation through a genome-wide analysis of methylation and transcriptomic data. The findings may provide a foundation for advancing pain management strategies.

**Materials and Methods**

Patients diagnosed with OUD were identified based on the criteria outlined in the Diagnostic and Statistical Manual of Mental Disorders, Fifth Edition (DSM-5) (24). Demographic and clinical-pathological data, such as age, sex, ethnicity, gestational age, and history of drug exposure, were gathered and this information has been previously published (25, 26). The study received approval from the Institutional Review Board of Beaumont Health System, Royal Oak, MI, USA (HIC#: 2019-086). Pregnant women were identified retrospectively via chart review from William Beaumont Hospital, Royal Oak, MI. Informed consent was waived since the study solely involved collecting discarded placental tissues and obtaining limited de-identified demographic data from hospital records.

The diagnosis of NOWS, coded as P96.1, was established by neonatologists following clinical criteria. Newborns born to mothers with a history of opioid or illicit drug use underwent monitoring in the inpatient unit for 4-5 days to detect signs of NOWS. Assessment of the infant's condition was conducted using the Finnegan Neonatal Abstinence Scoring Tool (FNAST), administered by postpartum and/or NICU nurses. If the FNAST scores indicated the need for pharmacologic intervention, the infant was transferred to the NICU for continued monitoring, scoring, and treatment. Throughout the treatment process, parental involvement was encouraged to optimize non-pharmacologic approaches, which remained the primary focus before and during pharmacologic treatment, regardless of the need for medication. The FNAST scoring system guided decisions regarding the initiation of pharmacologic management with morphine.

A total of 96 formalin-fixed, paraffin-embedded (FFPE) placental tissue biopsies were processed for DNA extraction. These samples were categorized into three distinct groups for analysis. Group 1 comprised 32 placental tissue samples from newborns exposed prenatally to opioids and requiring pharmacologic treatment for NOWS (+Opioids/+NOWS). Group 2 consisted of 32 placental tissue samples from newborns prenatally exposed to opioids but not requiring pharmacologic treatment for NOWS (+Opioids/-NOWS). Lastly, Group 3 served as the control, comprising placental tissue samples from newborns with no prenatal opioid exposure and no NOWS (-Opioids/-NOWS, control).

**Illumina Infinium MethylationEPIC BeadChip*.*** We performed genome-wide DNA methylation analysis on bisulfite-treated genomic DNA using the Illumina Infinium EPIC BeadChip (850K) according to the manufacturer’s protocol at the Beaumont Health Genomics Core Facility. The detailed methodology has been previously described (25, 26). To ensure the integrity of our microarray experiments, sample placement on arrays was randomized for both cases and controls age and gender (27). The EPIC array encompasses over 850,000 individual CpG sites across the genome at a single-nucleotide resolution, encompassing multiple genes linked to pain. Following scanning with Illumina iScan scanners, intensity data (iDAT) files were imported into Illumina’s Genome Studio methylation analysis package program.

**Statistical and bioinformatic analysis.** The IDAT files containing data were normalized using Genome Studio software's functional normalization method to ascertain Cytosine methylation levels (represented as ß-values) for each CpG site. Before analysis, CpG-probes with missing ß-values were excluded. Differential methylation was evaluated by comparing the ß-values of cytosines at individual CpG loci between NOWS and control groups. To minimize confounding variables, probes targeting sex chromosomes, non-specific regions, and CpG sites within 10 base pairs of SNPs were eliminated (28-30). Additionally, SNPs with a minor allele frequency of ≤0.05 were exclusively considered for subsequent analysis.

Distinguishing methylated CpG sites between individuals with NOWS and controls was determined using predetermined cutoff criteria with a false discovery rate (FDR) threshold of p<0.05. When multiple CpG sites were found within a gene, the one with the highest area under the curve (AUC) receiver operating characteristic (ROC) ranking and the lowest p-value was selected. The calculation of p-values for methylation disparities between the case and control groups at each locus followed previously established methods (25, 31). Both raw p-values and FDR-adjusted p-values, corrected for multiple testing using the Benjamini-Hochberg method, were computed. Additionally, the AUC for combinations of loci was computed using the "ROCR" package (version 3.5.0) in the 'R' programming environment.

**Chronic Pain-Associated Gene Selections.** Pain-associated genes were identified through a comprehensive analysis using an integrated genetic database. This database combines data from genome-wide association studies (GWAS), gene expression profiles, and curated literature on pain phenotypes (32, 33). The focus was on genes with robust GWAS evidence linking them to pain perception or chronic pain, genes documented in known pain pathways, and those associated with opioid use, particularly in the context of chronic pain management or opioid-induced hyperalgesia. Detailed information on these genes, including their functions, pathways, and relevant SNPs or mutations, was carefully extracted and cross-referenced with multiple databases to ensure accuracy and relevance, particularly in the context of opioid use, addiction, and chronic pain. This comprehensive approach highlights the genetic underpinnings that link opioid use disorder with pain-related pathways and addiction susceptibility. Additionally, to identify potential genes implicated in pain, relevant review articles were consulted and reviewed (34-38). These articles offered valuable insights by synthesizing existing research, with a particular focus on genes involved in pain perception, chronic pain, and opioid use. A total of 897 pain-associated genes were retrieved including genes from the Human Pain Genetics Database (<https://diatchenko.lab.mcgill.ca/hpgdb/>), a hand-curated resource of genetic associations with human pain phenotypes (Supplementary Table S1). Genes overlapping between this database and published literature were included only once.

Supplementary Table S2 presents details on several key genes and their biological roles associated with pain. The table categorizes these genes based on their involvement in various physiological and pathological processes, including diabetes, telomere maintenance, glucose metabolism, aging, and suicide, among others. Each entry provides information on the gene’s function and its relevance to the context of pain.

**Heatmap.** The heatmap was created with the Complex Heatmap module (v1.6.0) in the R package (v3.2.2). It displays the distribution of methylated CpG sites among pain-associated CpGs, where each site represents a single data point. This analysis aims to visually represent methylation patterns across these regions. Hierarchical clustering of the samples was performed using Ward's method (39). Significantly different methylated CpG sites between NOWS and control groups were determined using FDR P-values ≤0.05. The area under the receiver operating characteristic (AUC-ROC) was calculated based on methylation levels at the most significant CpG loci.

**Protein-Protein Interaction (PPI) Network Analysis.** We performed a PPI network analysis on the 111 differently methylated genes implicated in pain perception in NOWS using the STRING database (version 12.0). Parameters set for the analysis included a maximum false discovery rate (FDR) of 0.05 to focus on statistically significant interactions, a minimum interaction strength of 0.01 to include relevant but not necessarily strong interactions, and a minimum count of 2 to be displayed in the network, ensuring that only genes with at least one connection were included. Notably, the *MALAT1* gene (lncRNA), identified as a noncoding gene, was excluded from this analysis due to its inability to encode protein and hence participate in PPIs.

**Gene Ontology (GO) and KEGG Pathway Analysis**. The functional implications of these genes were further investigated through Gene Ontology (GO) and Kyoto Encyclopedia of Genes and Genomes (KEGG) pathway analyses via the DAVID v6.8 tool. Enrichment analysis was conducted, focusing on biological processes (BP), cellular components (CC), molecular functions (MF), and metabolic pathways. Significant terms and pathways (p-value < 0.05, Benjamini-Hochberg corrected) were identified to elucidate the biological basis of NOWS pain perception. Results from the GO and KEGG analyses were visualized using bubble plots in R using the ggplot2 package. Each bubble represents a GO term or KEGG pathway, with size indicating gene count and color showing significance, providing an intuitive view of the enriched biological themes.

**Venn Diagram Analysis.** A Venn diagram analysis was performed to identify unique and shared methylation patterns among genes associated with NOWS-associated pain. Gene lists from four independent analyses were compiled and visualized using InteractiVenn (40). This approach enabled the identification of genes unique to each analysis as well as those common across multiple analyses.

**Results**

The study's four analyses identified differentially methylated CpGs linked to pain-related genes, opioid exposure, and NOWS. The first analysis compared the (+Opioids/+NOWS) group with the (+Opioids/-NOWS), identifying 50 significant genes (Supplementary Table S3). The second analysis, which compared the combined (+Opioids/+NOWS) and (+Opioids/-NOWS) groups with the (-Opioids/-NOWS, control), yielded 42 significant genes (Supplementary Table S4). The third analysis compared the (+Opioids/+NOWS) group with the (-Opioids/-NOWS, control), identifying 40 dysregulated genes (Supplementary Table S5). Finally, the fourth analysis compared the (+Opioids/-NOWS) group with the (-Opioids/-NOWS, control) group, revealing 49 significant markers (Supplementary Table S6).

After combining and overlapping the results from the four analyses, a total of 111 CpG sites (FDR p-values ≤0.05), associated with 111 pain-related genes in individuals with NOWS, were identified. When multiple genes overlapped in the analyses, each was included only once. Of these, 54 CpG sites exhibited hypomethylation, while 57 showed hypermethylation, with each site included only once. (Table 1).

**Heatmap assessment.** The heatmap reveals two distinct clusters of CpGs, (i.e. NOWS and control) utilizing CpG methylation markers associated with pain sensation. One cluster is characteristic of NOWS patients, while the other corresponds to the control group. This strong observation provides significant backing to the idea that these methylation markers are dependable indicators for discriminating between individuals with NOWS and those without. These results validate the precision and effectiveness of the methylation markers in distinguishing between the two patient groups. Supplementary Figures S1-S4 illustrate four distinct analysis combinations.

**PPI Network Characteristics**. The Protein-Protein Interaction (PPI) network analysis, which incorporated 110 nodes after excluding the noncoding *MALAT1* gene, revealed significant connectivity among proteins implicated in pain perception in NOWS. The network consisted of 350 edges, which is notably higher than the expected number of edges (154), suggesting a non-random pattern of protein interactions. The average node degree was calculated to be 6.36, indicating a moderate level of interaction per protein, while the average local clustering coefficient was 0.402, reflecting the network's tendency to form clusters. Remarkably, the PPI enrichment p-value was less than 1.0e-16, indicating highly significant enrichment beyond chance. This suggests that the differentially methylated genes in NOWS are interconnected and likely play a substantial role in pain perception. Visual analysis of the PPI network revealed several highly connected nodes that may be key regulators in NOWS pathophysiology (Figure 1).

**Gene Ontology (GO) Analysis**. The GO analysis identified significant terms across Biological Process (BP) (Figure 2), Cellular Component (CC) (Figure 3), and Molecular Function (MF) (Figure 4), that provide insights into the molecular basis of pain perception in NOWS. Key Biological Processes highlighted include the positive regulation of cytosolic calcium ion concentration, calcium ion import across the plasma membrane, and vasodilation, which suggest alterations in ion regulation and vascular function may play a critical role in NOWS-associated pain. Protein phosphorylation and signal transduction were also prominent, indicative of complex signaling pathways involved in pain response. Terms related to cardiac muscle function and nerve development were notably enriched, reflecting the potential influence of these genes on the neurophysiological adaptations in NOWS.

In Cellular Components a significant enrichment in genes associated with the plasma membrane points to the importance of membrane-bound proteins and receptors in nociception. Other notable components included axons, neuronal cell bodies, and various synapse structures, such as glutamatergic synapses, suggesting a focus on neuron-specific structures and interactions. Significant Molecular Functions implicated in the data include scaffold protein binding and calmodulin binding, which may influence intracellular signaling pathways related to pain. The activity of voltage-gated calcium channels and protein kinase activities were also highlighted, suggesting potential targets for modulating neuronal excitability and signal transduction in pain pathways specific to NOWS.

The GO terms identified suggest that the differently methylated genes in NOWS are significantly involved in neural signaling and development, membrane dynamics, and intracellular signaling mechanisms These biological themes provide a molecular framework that may explain the altered perception of pain in NOWS and could help in guiding further research into targeted therapies for pain management in affected neonates (Supplementary Tables S7-S9).

**Pathway Analysis.** KEGG pathway analysis of the 111 differently methylated genes identified their involvement in 48 pathways (FDR p-value <0.05), with a primary focus on neuronal function and substance use—both highly relevant to pain (Supplementary Table S10). Notably, the cAMP and MAPK signaling pathways, which exhibited high gene counts, play crucial roles in cellular responses to external stimuli and pain modulation. The neuroactive ligand-receptor interaction pathway, also enriched with a substantial number of genes, suggests alterations in neurotransmitter dynamics that may influence pain perception. Additionally, the enrichment of multiple disease-related pathways, including cancer and various infections, underscores the broader impact of epigenetic modifications on cellular stress and immune responses, which may have implications for pain regulation and opioid withdrawal (Figure 5).

**Venn diagram.** The Venn diagram analysis revealed distinct and overlapping methylation patterns, illustrating the complex epigenetic landscape of NOWS-associated pain (Figure 6). Each analysis identified a unique set of differentially methylated genes, while several genes exhibited overlapping methylation changes, suggesting their involvement in shared biological pathways. Notably, the *PLCE1* gene was consistently methylated across all analyses, highlighting it as a core epigenetic marker for NOWS-related pain. These findings reveal key regulatory networks with therapeutic potential. See Supplementary Table S11 for the full gene list.

**Discussion**

Pregnant women may use opioids for pain relief, addiction, or treatment, but misuse can lead to epigenetic changes that impact fetal development, affecting genes related to pain sensitivity and neurological functions (41, 42). Epigenetic changes during critical periods of fetal development can shape pain perception and increase the likelihood of severe health issues that may arise in adulthood, often rooted in early infancy (33). Yet, how prenatal opioid exposure disrupts epigenetic regulation and its lasting effects on pain remains unknown.

**Autophagy genes contribute to chronic pain.** We identified ten dysregulated autophagy-related genes—*BDNF, LMX1B, ESR2, ULK4, ATG5, BECN1, MAPK3, PIK3C3, CTSS,* and *ULK1*—that affect neuronal development, pain sensitivity, and long-term health. *BDNF* is essential for neuronal survival and plasticity (43). *LMX1B* and *ESR2* support serotonergic neuron development and pain modulation. Dysregulation of *LMX1B* increases pain sensitivity and mood disorder risk, while *ESR2* affects pain thresholds and mood via estrogen signaling, underscoring their role in the complex interplay of pain and mood regulation (44). *ULK4* and *ULK1* are critical enzymes that initiate autophagy, dysregulation of *ULK1* or *ULK4* impairs autophagy, causing damaged proteins to accumulate in neurons. This can lead to neurodegeneration, increased cellular stress, inflammation, and heightened pain sensitivity, raising the risk of chronic pain (45). *ATG5* is vital for autophagosome formation; abnormal expression can impair autophagy, causing neuroinflammation and chronic pain (46). *BECN1* regulates autophagy initiation; dysregulation may result in neuron damage and an increased risk of neurodegenerative diseases (47). *MAPK3,* part of the *MAPK/ERK* pathway, affects pain perception and neural plasticity, impacting cognitive function (48). *PIK3C3* is involved in autophagy and endocytic trafficking; dysfunction may lead to neuroinflammation and chronic pain (49). *CTSS*, linked to neuropathic pain, may increase neuroinflammation and affect immune function (50).

**Cytokines may affect pain perception.** Dysregulation of cytokine genes, such as *IL23R*, can alter pain perception through immune modulation (51); while *NR3C1*, encoding the glucocorticoid receptor, affects pain pathways and inflammation, with its dysregulation potentially leading to inflammatory pain (52). Reduced *NR3C1* expression is associated with chronic cocaine use and linked to anxiety disorders and depression (53); *MAPK3* (*ERK1*) is key in the MAPK pathway, facilitating pain signal transmission and modulating the nociceptive pathway, crucial for pain perception regulation (48); *TNF* and *IL-1β*, key pro-inflammatory cytokines, play crucial roles in glial cell function, aging, obesity, depression, and pain modulation by heightening oxidative stress, exacerbating neuroinflammation, and increasing pain perception (54); *NGF* promotes neuropathic pain through nerve sensitization and sprouting (55); *IL-18* and its receptor *IL18R1* may contribute to pain signaling, diverging from their traditional roles in the immune system (56); *CXCL8* promotes inflammatory pain through neutrophil recruitment (57); *BDNF*, a neurotrophin, is crucial in numerous pain conditions, especially neuropathic pain (58), and is linked to obesity, type 2 diabetes mellitus, and other facets of metabolic syndrome (59); *TRPV4*, a cation channel, plays a role in nociception and pain sensation (60); The lncRNA *MALAT1* is significantly upregulated in the spinal cord and microglia, making it a key player in pain regulation (61), Its modulation of miR-129-5p and *HMGB1* further highlights its role in neuropathic pain mechanisms (62). Estrogen receptors *ERα* and *ERβ*, encoded by *ESR1* and *ESR2*, are essential for pain modulation and metabolic functions, including glucose metabolism and insulin sensitivity with their dysregulation affecting both pain sensitivity and overall metabolic processes (63). Variants in *ESR1* and *ESR2* genes affect menopause timing and symptom severity, with *ESR1* influencing the onset and *ESR2* affecting symptoms like hot flashes and bone density (64). In women with opioid addiction, these variants are linked to irregular menstruation, early menopause, worsened symptoms, and increased anxiety, depression, and chronic pain (65, 66); *CAMK4*, a kinase in calcium signaling, is linked to pain processing and synaptic plasticity (67); *OSM*, a cytokine, contributes to inflammatory and neuropathic pain (68); while *TGFBR2*, a receptor for *TGF-β*, is involved in pain-related cellular processes (69). Also, *RUNX1*, a transcription factor, affects sensory neuron development and pain processing (70).

**Chronic pain can trigger addiction.** Chronic pain frequently leads to opioid and alcohol use, disrupting brain chemistry and causing dependence (71). We identified 22 dysregulated pain-related genes in infants exposed to opioids in utero, affecting neurodevelopment, stress response, and reward pathways, thereby increasing the likelihood of addiction and opioid use later in life. *SCN8A* affects neuronal excitability, increasing addiction risk (72), while *BDNF* influences dopamine signaling, with lower levels linked to vulnerability (73). Changes in *OPRM1* may alter opioid sensitivity (74), and variants in *ANKK1/DRD2* disrupt dopamine signaling, increasing susceptibility to addiction(75). *NPY* impacts stress responses (76), and *MAPK* dysregulation affects synaptic plasticity (77). Variants in *NR3C1*, calcium channels *(CACNA1C, CACNA1H, CACNA2D1*), and GABAergic genes (*ABRG2, GRIA1*) further influence addiction pathways*. PRKAR1B, NRXN3, SHANK3*, and immune genes (*TGFBR2, IL1B*, *TNF*) contribute to addiction risk, with autophagy-related genes (*BECN1, ULK1, ULK4*) may contribute to neurodegeneration, as previously detailed (78-80).

**Chronic pain can lead to depression and suicidality.** Chronic pain can lead to depression, a major risk factor for suicidality (81). We found multiple deregulated genes involved in pain, such as *TNF, IL1B*, and *IL6ST* (82-84), were identified as being associated with both depression and suicide. Gene variations affecting inflammation play a key role in depression, linking chronic pain, mood disorders, and suicide risk. Suicide is also reported in conditions like major depressive disorder, Alzheimer's disease, and Autism Spectrum Disorder. In our study, we identified several key genes associated with suicidal behavior, such as *BDNF, KCNJ2, NOS3, CACNA1C, PCSK5, GRIA1, ESR1, ANKK1, DLG2, GABRG2, IGSF9B, PRDM16, and NR3C1*. Variations in these genes may contribute to the complex interaction of biological, psychological, and environmental factors that may increase suicide risk in individuals in later life (83, 85-88).

**Telomere genes in Pain.** Telomere maintenance genes, essential for preserving telomere length and integrity, have emerged as potential modulators of pain sensitivity (89). As telomeres shorten with age and stress, they are linked to cellular aging, and increased inflammation, and can influence pain perception (90). The dysregulated genes identified in this study, including *NOS3, CFTR, ESR1, MAPK3, MTA1, ESR2, PRDM16, IKBKAP, RUNX2, REST, DLG2, PIK3C3, SHMT1,* and *PLCE1*, may impact pain perception by affecting inflammation, oxidative stress, and neuronal excitability pathways.

**Pain can promote** **impulsivity.** "Pain can lead to impulsive behavior by affecting decision-making and self-control (91). Those experiencing pain may engage in risky activities, such as overusing opioids, which can result in addiction(1). Pain also amplifies emotional distress and weakens self-regulation, further increasing impulsivity (92). We identified five dysregulated genes associated with impulsivity: *BDNF, MAPK10, NRG1, NRXN3,* and *PRKG1*. *BDNF,* a key biomarker for impulsivity, regulates impulse control by shaping neural circuits for decision-making (93). *MAPK10* influences impulsivity through stress pathways and brain regions controlling behavior. *NRG1* affects synaptic networks tied to impulse control (94), while *NRXN3* variants alter synaptic efficacy, contributing to impulsivity (95). *PRKG1* disrupts synaptic plasticity and neurotransmitter release, impacting impulse control (96).

**Glucose metabolism.** Glucose metabolism is crucial for physiological functions, including pain perception, possibly due to glucose level fluctuations (97, 98). Glucose metabolism and pain are bidirectional; elevated glucose levels can reduce pain tolerance, as seen when glucose infusion lowers pain thresholds (98). Diabetic patients often experience heightened pain sensitivity (hyperalgesia), linking altered glucose metabolism to pain perception (98). Conversely, acute severe pain decreases insulin sensitivity by affecting nonoxidative glucose metabolism, indicating that pain influences glucose metabolism (99). The hormonal response to pain, involving stress hormones, underscores the importance of pain management in maintaining metabolic balance (99). The study identified genes *like NR3C1, CACNA1C, CACNA1H, ESR1, CFTR, KLF11, EIF2AK3, NOTCH3, GJA1,* and *STX1A* as linked to glucose metabolism and pain sensitivity. *NR3C1* mediates glucocorticoids' anti-inflammatory and analgesic effects, affecting glucose metabolism and pain (100). *CACNA1C* and *CACNA1H*, which encode voltage-gated calcium channels, influence neurotransmitter release and pain sensitivity through glucose balance disruption (101). CFTR, linked to cystic fibrosis, causes metabolic irregularities that impact pain (102)*. KLF11* regulates glucose metabolism and insulin, with variations affecting pain sensitivity and diabetes (103). *EIF2AK3*, involved in ER stress and glucose metabolism, is associated with chronic pain (104). *NOTCH3*, a cell signaling receptor, may modulate pain through interactions with metabolic regulators (105). *GJA1,* a gap junction protein, influences glucose metabolism and pain signaling. *STX1A*, important for neurotransmitter release, affects pain sensitivity and glucose metabolism (106).

**Obesity**. Obesity worsens chronic pain by increasing inflammation and aggravating conditions like rheumatoid arthritis and fibromyalgia, while also raising the risk of metabolic syndrome, heart issues, diabetes, and cancer (107). This bidirectional relationship fuels a cycle where obesity heightens pain and reduces physical function, while chronic pain limits activity, contributing to weight gain and further exacerbating both conditions (108). Our study identifies that dysregulation in genes like *NOS3, TNF, BDNF, ESR1, ESR2, LMX1B, ATP1A2, OPRM1, NPY, THRB, NRXN3, SPARC,* and *MYT1L* significantly impacts the interplay between obesity and chronic pain. Earlier discussions covered *NOS3, TNF, BDNF, ESR1,* and *ESR2. LMX1B* plays a key role in obesity by regulating adipogenesis and energy metabolism (109). *LMX1B* mutations linked to nail-patella syndrome (NPS) may also contribute to chronic pain, suggesting heightened pain sensitivity (110); *ATP1A2* helps regulate metabolism by maintaining ion gradients across cell membranes and is linked to pain disorders such as neuropathic pain and fibromyalgia. The *OPRM1* gene, linked to the mu-opioid receptor, affects obesity and pain, especially in NOWS infants, with prolonged opioid use disrupting appetite and metabolism, leading to weight gain (111); *NPY* regulates appetite and energy balance, promoting feeding behavior and fat storage, thereby contributing to obesity development; *THRB* regulates metabolism and energy expenditure through thyroid hormones, affecting metabolic rate and potentially causing obesity if dysregulated. It also influences pain sensitivity and inflammation; *NRXN3* affects obesity traits like BMI and adiposity through appetite-regulating neural circuits and is linked to pain sensitivity (112). *SPARC* regulates tissue remodeling, impacting obesity-related processes and pain modulation. *MYT1L*, a transcription factor important for neural development, is associated with intellectual disability, autism, and obesity (113).

**Circadian Rhythm**. Circadian rhythms, which regulate body processes over a 24-hour cycle, significantly affect pain perception and management (114). Disrupted rhythms can worsen pain and alter opioid processing, while pain and opioids can also impact circadian rhythms (115, 116). Disruptions affect factors like endogenous opioid levels, sleep, gene expression, and hormonal regulation. We identified 12 genes linking circadian rhythms to pain regulation: *ESR1, CACNA1C, KLF11, IL1B, ABCA1, CAMK4, OSM, SHMT1, ARNTL, DOCK4, GNA11,* and *CRIP2.*  *ESR1* affects females' circadian systems via estrogen levels. *CACNA1C* modulates calcium influx, potentially disrupting rhythms. *KLF11* influences glucose metabolism, impacting circadian patterns. *IL1B*'s proinflammatory effects affect the *SCN*, disrupting rhythms. *ABCA1* affects lipid signaling, possibly disrupting rhythms (117). *CAMK4* synchronizes peripheral clocks. *OSM* modulates rhythms through inflammatory pathways (68). *SHMT1* impacts metabolic processes. *ARNTL*, the core clock gene, regulates other clock genes, and mutations cause disorders. *DOCK4*, associated with neuronal development, may regulate rhythms. *GNA11* influences light signaling pathways. *CRIP2* affects rhythmic gene expression in the heart, potentially disrupting circadian rhythms (118).

**Essential Minerals and Electrolytes in Pain Management.** Minerals and electrolytes are essential for nerve function and pain regulation, governing nerve signaling and muscle contractions, while dysregulated metal-associated genes can disrupt DNA methylation and exacerbate chronic pain conditions (119). Calcium ions, crucial for neurotransmitter release and signaling, are disrupted by dysregulated genes such as *CACNA1C* and *CACNA1H*, heightening pain sensitivity and altering pain thresholds (120). Potassium channels (e.g., *KCNJ2, KCNQ5*) regulate neuronal excitability by maintaining membrane potential (121), while sodium channels (e.g., *SCN1A, SCN8A*) are vital for action potential transmission and pain signaling (122). Disruptions in these channels due to genetic variations or prenatal opioid exposure can lead to abnormal pain signaling. Selenium (*PCSK5, BDNF*) aids in antioxidant defense (123) and neurotransmitter regulation, potentially reducing chronic pain risk and neurodevelopmental issues from opioid withdrawal. Lithium (*BDNF*) influences neurogenesis and pain regulation by affecting BDNF levels, impacting the nervous system's adaptation to pain in opioid-exposed infants (124). *ATP1A2* encodes a Na+/K+ ATPase subunit crucial for ion balance, and disruptions can impact pain processing and heighten the risk of pain or neurological issues such as migraine later in life (125).

**Transporters can influence pain perception.** Dysregulated transporters like *ABCA1, SLC12A5*, and *ABCC7 (CFTR),* critically affect infants exposed to opioids in utero by altering neurotransmitter and ion movement across cell membranes (9). *ABCA1* affects lipid metabolism and cholesterol transport. Altered *ABCA1* can disrupt lipid balance, increasing inflammation and pain sensitivity, and potentially impacting nervous system development and long-term pain processing (126). *SLC12A5* encodes the K+-Cl- cotransporter KCC2, essential for maintaining chloride homeostasis in neurons, which is critical for proper synaptic inhibition and neuronal excitability. Impaired *SLC12A5* function can lead to altered pain perception and increased sensitivity, effects that may be aggravated in individuals with a history of opioid exposure (127). *ABCC7 (CFTR)* regulates chloride ion transport across epithelial membranes, impacting tissue homeostasis and inflammation. While primarily associated with cystic fibrosis, *CFTR* abnormalities can indirectly influence pain pathways by promoting chronic inflammation and epithelial dysfunction, leading to altered pain sensitivity and management (128).

**Pathways.** Our study identified 48 significant canonical pathways (FDR < 0.05). We highlight key findings relevant to pain and infants born after prenatal opioid exposure.

**cAMP signaling pathway**. The cAMP signaling pathway is crucial for pain perception and transmission in the nervous system. Activating the cAMP pathway in the central nervous system induces hyperalgesia, whereas inhibiting this pathway alleviates hyperalgesia in inflammatory, non-inflammatory, and neuropathic pain models (129). There were 14 genes identified in this pathway.

**MAPK signaling pathway.** The MAPK pathway regulates diverse cellular functions and is crucial for transmitting pain signals from the injury site to the brain, where they are interpreted as pain (48). Thirteen differentially methylated genes have been identified in this pathway.

**AGE-RAGE Signaling Pathway in Diabetic Complications:** The AGE-RAGE pathway is critical in diabetic complications, including pain (130). Advanced Glycation End-products (AGEs) accumulate in tissues due to hyperglycemia, activating RAGE and initiating signaling cascades (131). In diabetic pain, this pathway contributes to neuroinflammation, oxidative stress, and nerve damage, which are key factors in diabetic neuropathy. RAGE activation leads to the production of pro-inflammatory cytokines, chemokines, and reactive oxygen species (ROS), exacerbating neuroinflammation and contributing to nerve dysfunction and pain hypersensitivity (132). Eight genes are involved in this pathway. Neurodegeneration pathways in NOWS, like oxidative stress, inflammation, and apoptosis, may alter long-term pain sensitivity and modulation (133). Infants with NOWS might experience increased pain sensitivity or altered pain responses, potentially persisting into childhood and adulthood. Calcium signaling and Alzheimer's disease-related pathways, essential for neural function, are disrupted in NOWS (134, 135). These disruptions, combined with epigenetic changes, may contribute to heightened pain sensitivity and neurological dysfunction in affected infants. The PI3K-Akt pathway, vital for cell survival and growth, significantly influences pain processes. It can drive both adaptive responses that manage pain and maladaptive processes that worsen chronic pain (136). The enrichment of oxytocin signaling and circadian entrainment pathways in NOWS affects both pain perception and broader physiological systems in neonates, necessitating comprehensive care strategies (137, 138).

**Limitations.** Current human placental studies are inadequate for identifying biomarkers, and the underlying epigenetic mechanisms are not well understood. While these studies offer preliminary evidence linking therapeutic opioid use to epigenetic changes, further research is essential to investigate the temporal dynamics of these modifications in response to both prescription opioids and pain. Establishing causality will require additional factors to be considered. This study provides an intriguing potential proof-of concept, but larger cohort studies are necessary to validate these findings. Moreover, the differentially methylated genes identified have not been subjected to further validation.

In conclusion, our cohort study revealed that opioid use for pain management during pregnancy leads to disruptions in epigenetic factors. These alterations could serve as biomarkers, offering potential therapeutic targets and insights into the molecular mechanisms underlying prescription opioid use and pain. Notably, further investigation is needed to understand how epigenetic changes in genes related to chronic pain, due to maternal opioid use during pregnancy, affect NOWS. Looking ahead, our future research will focus on validating key epigenetic markers through functional studies, exploring their therapeutic potential in preclinical models, and assessing the long-term impact of these markers on individuals exposed to opioids.

**Data Availability Statement**: The published article and its supplementary materials contain all the data generated during this study.

**Conflicts of Interest:** The authors report there are no competing interests to declare. Efforts of Drs. Sadhasivam and Radhakrishnan are supported by their collaborative NIH grants (R01HD096800 PI: Sadhasivam) and R01DA059321 PI: Dr. Radhakrishnan). Dr. Sadhasivam’s efforts are also supported by R44DA055407, R44DA056280, R41DA053877 (MPI: Sadhasivam), R01DA054513 (MPI: Chelly/ Sadhasivam) and U01TR003719 (PI: Sadhasivam).

**Figure Legends**

**Figure 1:** Protein-Protein Interaction (PPI) Network Analysis Depicting 110 Nodes and 350 Edges. The network demonstrates a higher-than-expected level of connectivity with an average node degree of 6.36 and an average local clustering coefficient of 0.402, indicative of a tightly knit network. This is supported by a PPI enrichment p-value of less than 1.0e-16.

**Figure 2:** Displays the enrichment analysis for Biological Processes (BP). The size of each bubble corresponds to the number of genes associated with that term, and the color gradient represents the FDR value, with darker shades indicating greater significance.

**Figure 3:** Shows the Cellular Components (CC) with significant gene involvement. In all subfigures, the size of each bubble corresponds to the number of genes associated with that term, and the color gradient represents the FDR value, with darker shades indicating greater significance.

**Figure-4.** Bubble plots of molecular functions (MF) enrichment analysis

**Figure-5.** Gene ontology enrichment and KEGG pathway analysis of differentially methylated genes in NOWS cases, compared to normal controls, highlight significant involvement in pain-related pathways. The size of the bubble corresponds to the number of enriched genes within each pathway.

**Figure-6.** Venn diagram showing the overlap of differentially methylated genes across four analyses, each comparing methylation patterns related to NOWS-associated pain.

- Analysis I: Compares (+Opioids/+NOWS) vs. (+Opioids/-NOWS) to identify methylation differences linked to NOWS-associated pain.
- Analysis II: Compares (+Opioids/+NOWS) and (+Opioids/-NOWS) vs. (-Opioids/-NOWS, control) to identify CpG targets associated with pain.
- Analysis III: Compares (+Opioids/+NOWS) vs. (-Opioids/-NOWS, control) to pinpoint methylation changes in NOWS-related pain genes.
- Analysis IV: Compares (+Opioids/-NOWS) vs. (-Opioids/-NOWS, control) to identify CpG targets with differential methylation in pain-related genes.

**Table headings**

**Table-1.** A comprehensive list of 111 CpG targets exhibiting significant methylation differences in NOWS is provided. This includes CpG sites with Target ID, pain-associated gene IDs, chromosome locations, p-values, FDR p-values, and the percentage of methylation difference.

**Supplementary Figures**

**Supplementary** **Figure-1**. Heatmap of selected highly differentially methylated CpGs between +Opioids/+NOWS versus +Opioids/-NOWS. DNA methylation profiling, through unsupervised hierarchical clustering, delineated distinctive clusters with unique methylation patterns. The accompanying figure illustrates the directionality, probe relationships, and annotations of differentially methylated CpG sites.

**Supplementary Figure-2**. Unsupervised hierarchical clustering and a heatmap were generated to compare the methylation levels of significantly differentially methylated regions associated with pain among +Opioids/+NOWS, +Opioids/-NOWS, and -Opioids/-NOWS (control) groups.

**Supplementary Figure-3**. Unsupervised hierarchical clustering and heatmap analysis compared methylation levels related to pain between (+Opioids/+NOWS) and (-Opioids/-NOWS, control), highlighting significant CpG targets in genes linked to NOWS-induced pain.

**Supplementary Figure-4.** Unsupervised hierarchical clustering and heatmap analysis compared methylation levels of differentially methylated regions related to pain between (+Opioids/-NOWS) and (-Opioids/-NOWS, control), revealing significant methylation differences in genes associated with NOWS-induced pain.

**Supplementary Tables**

**Supplementary Table S1.** A comprehensive list of pain-associated genes was compiled from published data and relevant references and compared with our study findings.

**Supplementary Table S2**. Pain-Related Genes list and their and their biological roles. This table provides an extensive list of genes associated with pain, highlighting their specific biological roles. The genes are categorized based on their involvement in various physiological and pathological processes, including diabetes, telomere maintenance, glucose metabolism, aging, and suicide, among others. Each entry details the gene’s function and its relevance to the context of pain.

**Supplementary Table-S3**. Investigation into +Opioids/+NOWS and +Opioids/-NOWS highlights distinct variations in the methylation status linked with NOWS-induced pain.

**Supplementary Table-S4**. Examining the (+Opioids/+NOWS) group, (+Opioids/-NOWS) group, and the (-Opioids/-NOWS, control) group reveals CpG targets linked with pain.

**Supplementary Table-S5**. Analysis of (+Opioids/+NOWS) versus (-Opioids/-NOWS, control), highlighting CpG targets showing significant methylation differences in genes associated with pain linked to NOWS.

**Supplementary Table-S6.** Comparison between (+Opioids/-NOWS) and (-Opioids/-NOWS, control) elucidates CpG targets that exhibit significant differential methylation in genes linked to pain in NOWS.

**Supplementary Table-S7.** Biological processes (BP) enrichment analysis**.**

**Supplementary Table-S8.** Cellular Component (CC) enrichment analysis**.**

**Supplementary Table-S9.** Molecular function (MF) enrichment analysis.

**Supplementary Table-S10.** Gene ontology enrichment analysis and KEGG pathway

**Supplementary Table-S11.** Differentially Methylated Genes Identified Across Four Analyses and Their Overlap (Venn Diagram Representation).

**Analysis 1**. **Description of opioid use in opioid‐exposed infants with and without NOWS:** Comparison of infants requiring pharmacologic management for NOWS +Opioids/+NOWS) versus those who did not require treatment (+Opioids/–NOWS). This analysis identifies epigenetic and molecular differences between opioid-exposed newborns with NOWS requiring treatment and those without withdrawal, pinpointing factors that trigger withdrawal.

**Analysis 2.** **Comparison of Opioid-Exposed Infants vs. Unexposed Controls:**This analysis compares opioid-exposed infants—both those requiring pharmacologic management for NOWS (+Opioids/+NOWS) and those not requiring treatment (+Opioids/-NOWS)—with unexposed controls (-Opioids/-NOWS). Understanding these variations helps uncover prenatal opioid exposure and its influence on the newborn's molecular and epigenetic makeup.

**Analysis 3.** **NOWS vs unexposed controls.** (opioid-exposed infants who required pharmacologic management for NOWS versus unexposed controls (+Opioids/+NOWS versus -opioids/-NOWS, control), revealing key markers underlying withdrawal symptoms.

**Analysis 4.** **Distinguishing opioid-induced epigenetic changes**: Comparison between NOWS infants who do not require pharmacologic management (+Opioids/-NOWS) versus (-Opioids/-NOWS, control) revealing key opioid-induced alterations that illuminate early molecular mechanisms.

**References**

1. Dowell D, Ragan KR, Jones CM, Baldwin GT, Chou R. Cdc Clinical Practice Guideline for Prescribing Opioids for Pain - United States, 2022. *MMWR Recomm Rep* (2022) 71(3):1-95. Epub 2022/11/04. doi: 10.15585/mmwr.rr7103a1.

2. Dourson AJ, Willits A, Raut NGR, Kader L, Young E, Jankowski MP, et al. Genetic and Epigenetic Mechanisms Influencing Acute to Chronic Postsurgical Pain Transitions in Pediatrics: Preclinical to Clinical Evidence. *Can J Pain* (2022) 6(2):85-107. Epub 2022/05/17. doi: 10.1080/24740527.2021.2021799.

3. Amaya F, Izumi Y, Matsuda M, Sasaki M. Tissue Injury and Related Mediators of Pain Exacerbation. *Curr Neuropharmacol* (2013) 11(6):592-7. Epub 2014/01/08. doi: 10.2174/1570159X11311060003.

4. Bonezzi C, Fornasari D, Cricelli C, Magni A, Ventriglia G. Not All Pain Is Created Equal: Basic Definitions and Diagnostic Work-Up. *Pain Ther* (2020) 9(Suppl 1):1-15. Epub 2020/12/15. doi: 10.1007/s40122-020-00217-w.

5. Campbell JN, Meyer RA. Mechanisms of Neuropathic Pain. *Neuron* (2006) 52(1):77-92. Epub 2006/10/04. doi: 10.1016/j.neuron.2006.09.021.

6. Reddy D, Wickman JR, Ajit SK. Epigenetic Regulation in Opioid Induced Hyperalgesia. *Neurobiology of Pain* (2023) 14:100146. doi: <https://doi.org/10.1016/j.ynpai.2023.100146>.

7. Alorfi NM. Pharmacological Methods of Pain Management: Narrative Review of Medication Used. *Int J Gen Med* (2023) 16:3247-56. Epub 2023/08/07. doi: 10.2147/IJGM.S419239.

8. Savage SR, Kirsh KL, Passik SD. Challenges in Using Opioids to Treat Pain in Persons with Substance Use Disorders. *Addict Sci Clin Pract* (2008) 4(2):4-25. Epub 2008/05/24. doi: 10.1151/ascp08424.

9. Radhakrishna U, Radhakrishnan R, Uppala LV, Muvvala SB, Prajapati J, Rawal RM, et al. Prenatal Opioid Exposure Significantly Impacts Placental Protein Kinase C (Pkc) and Drug Transporters, Leading to Drug Resistance and Neonatal Opioid Withdrawal Syndrome. *Front Neurosci* (2024) 18:1442915. Epub 2024/09/06. doi: 10.3389/fnins.2024.1442915.

10. Radhakrishna U, Sadhasivam S, Radhakrishnan R, Forray A, Muvvala SB, Metpally RP, et al. Placental Cytochrome P450 Methylomes in Infants Exposed to Prenatal Opioids: Exploring the Effects of Neonatal Opioid Withdrawal Syndrome on Health Horizons. *Front Genet* (2023) 14:1292148. Epub 2024/01/24. doi: 10.3389/fgene.2023.1292148.

11. Radhakrishna U, Nath SK, Uppala LV, Veerappa A, Forray A, Muvvala SB, et al. Placental Microrna Methylome Signatures May Serve as Biomarkers and Therapeutic Targets for Prenatally Opioid-Exposed Infants with Neonatal Opioid Withdrawal Syndrome. *Front Genet* (2023) 14:1215472. Epub 2023/07/12. doi: 10.3389/fgene.2023.1215472.

12. Conradt E, Flannery T, Aschner JL, Annett RD, Croen LA, Duarte CS, et al. Prenatal Opioid Exposure: Neurodevelopmental Consequences and Future Research Priorities. *Pediatrics* (2019) 144(3). Epub 2019/08/30. doi: 10.1542/peds.2019-0128.

13. Metpally RP, Krishnamurthy S, Moran KM, Weller AE, Crist RC, Reiner BC, et al. The Imperative of Clinical and Molecular Research on Neonatal Opioid Withdrawal Syndrome. *Mol Psychiatry* (2019) 24(11):1568-71. Epub 2019/09/12. doi: 10.1038/s41380-019-0522-1.

14. Wanner NM, Colwell ML, Faulk C. The Epigenetic Legacy of Illicit Drugs: Developmental Exposures and Late-Life Phenotypes. *Environ Epigenet* (2019) 5(4):dvz022. Epub 2019/11/30. doi: 10.1093/eep/dvz022.

15. Etemadi-Aleagha A, Akhgari M. Psychotropic Drug Abuse in Pregnancy and Its Impact on Child Neurodevelopment: A Review. *World J Clin Pediatr* (2022) 11(1):1-13. Epub 2022/02/01. doi: 10.5409/wjcp.v11.i1.1.

16. Gouin K, Murphy K, Shah PS, Knowledge Synthesis group on Determinants of Low Birth W, Preterm B. Effects of Cocaine Use During Pregnancy on Low Birthweight and Preterm Birth: Systematic Review and Metaanalyses. *Am J Obstet Gynecol* (2011) 204(4):340 e1-12. Epub 2011/01/25. doi: 10.1016/j.ajog.2010.11.013.

17. Seo S, Grzenda A, Lomberk G, Ou XM, Cruciani RA, Urrutia R. Epigenetics: A Promising Paradigm for Better Understanding and Managing Pain. *J Pain* (2013) 14(6):549-57. Epub 2013/04/23. doi: 10.1016/j.jpain.2013.01.772.

18. Nirvanie-Persaud L, Millis RM. Epigenetics and Pain: New Insights to an Old Problem. *Cureus* (2022) 14(9):e29353. Epub 2022/09/27. doi: 10.7759/cureus.29353.

19. Descalzi G, Ikegami D, Ushijima T, Nestler EJ, Zachariou V, Narita M. Epigenetic Mechanisms of Chronic Pain. *Trends Neurosci* (2015) 38(4):237-46. Epub 2015/03/15. doi: 10.1016/j.tins.2015.02.001.

20. Xiong HY, Wyns A, Campenhout JV, Hendrix J, De Bruyne E, Godderis L, et al. Epigenetic Landscapes of Pain: DNA Methylation Dynamics in Chronic Pain. *Int J Mol Sci* (2024) 25(15). Epub 2024/08/10. doi: 10.3390/ijms25158324.

21. Lee MC, Wanigasekera V, Tracey I. Imaging Opioid Analgesia in the Human Brain and Its Potential Relevance for Understanding Opioid Use in Chronic Pain. *Neuropharmacology* (2014) 84(100):123-30. Epub 2013/07/31. doi: 10.1016/j.neuropharm.2013.06.035.

22. Massart R, Dymov S, Millecamps M, Suderman M, Gregoire S, Koenigs K, et al. Overlapping Signatures of Chronic Pain in the DNA Methylation Landscape of Prefrontal Cortex and Peripheral T Cells. *Sci Rep* (2016) 6:19615. Epub 2016/01/29. doi: 10.1038/srep19615.

23. De Ridder D, Adhia D, Vanneste S. The Anatomy of Pain and Suffering in the Brain and Its Clinical Implications. *Neurosci Biobehav Rev* (2021) 130:125-46. Epub 2021/08/20. doi: 10.1016/j.neubiorev.2021.08.013.

24. Hasin DS, O'Brien CP, Auriacombe M, Borges G, Bucholz K, Budney A, et al. Dsm-5 Criteria for Substance Use Disorders: Recommendations and Rationale. *Am J Psychiatry* (2013) 170(8):834-51. Epub 2013/08/02. doi: 10.1176/appi.ajp.2013.12060782.

25. Radhakrishna U, Vishweswaraiah S, Uppala LV, Szymanska M, Macknis J, Kumar S, et al. Placental DNA Methylation Profiles in Opioid-Exposed Pregnancies and Associations with the Neonatal Opioid Withdrawal Syndrome. *Genomics* (2021) 113(3):1127-35. Epub 2021/03/13. doi: 10.1016/j.ygeno.2021.03.006.

26. Radhakrishna U, Nath SK, Vishweswaraiah S, Uppala LV, Forray A, Muvvala SB, et al. Maternal Opioid Use Disorder: Placental Transcriptome Analysis for Neonatal Opioid Withdrawal Syndrome. *Genomics* (2021) 113(6):3610-7. Epub 2021/08/06. doi: 10.1016/j.ygeno.2021.08.001.

27. Verdugo RA, Deschepper CF, Munoz G, Pomp D, Churchill GA. Importance of Randomization in Microarray Experimental Designs with Illumina Platforms. *Nucleic Acids Res* (2009) 37(17):5610-8. Epub 2009/07/21. doi: 10.1093/nar/gkp573.

28. Liu Y, Aryee MJ, Padyukov L, Fallin MD, Hesselberg E, Runarsson A, et al. Epigenome-Wide Association Data Implicate DNA Methylation as an Intermediary of Genetic Risk in Rheumatoid Arthritis. *Nat Biotechnol* (2013) 31(2):142-7. doi: 10.1038/nbt.2487.

29. Zhang C, Wang L, Chen L, Ren W, Mei A, Chen X, et al. Two Novel Mutations of the Ncstn Gene in Chinese Familial Acne Inverse. *J Eur Acad Dermatol Venereol* (2013) 27(12):1571-4. doi: 10.1111/j.1468-3083.2012.04627.x.

30. Wilhelm-Benartzi CS, Koestler DC, Karagas MR, Flanagan JM, Christensen BC, Kelsey KT, et al. Review of Processing and Analysis Methods for DNA Methylation Array Data. *Br J Cancer* (2013) 109(6):1394-402. doi: 10.1038/bjc.2013.496.

31. Vishweswaraiah S, Swierkowska J, Ratnamala U, Mishra NK, Guda C, Chettiar SS, et al. Epigenetically Dysregulated Genes and Pathways Implicated in the Pathogenesis of Non-Syndromic High Myopia. *Scientific reports* (2019) 9(1):4145. doi: 10.1038/s41598-019-40299-x.

32. Parisien M, Khoury S, Chabot-Dore AJ, Sotocinal SG, Slade GD, Smith SB, et al. Effect of Human Genetic Variability on Gene Expression in Dorsal Root Ganglia and Association with Pain Phenotypes. *Cell Rep* (2017) 19(9):1940-52. Epub 2017/06/01. doi: 10.1016/j.celrep.2017.05.018.

33. James S. Human Pain and Genetics: Some Basics. *Br J Pain* (2013) 7(4):171-8. Epub 2013/11/01. doi: 10.1177/2049463713506408.

34. Bai G, Ren K, Dubner R. Epigenetic Regulation of Persistent Pain. *Transl Res* (2015) 165(1):177-99. Epub 2014/06/21. doi: 10.1016/j.trsl.2014.05.012.

35. Linnstaedt SD, Rueckeis CA, Riker KD, Pan Y, Wu A, Yu S, et al. Microrna-19b Predicts Widespread Pain and Posttraumatic Stress Symptom Risk in a Sex-Dependent Manner Following Trauma Exposure. *Pain* (2020) 161(1):47-60. Epub 2019/10/01. doi: 10.1097/j.pain.0000000000001709.

36. Zorina-Lichtenwalter K, Meloto CB, Khoury S, Diatchenko L. Genetic Predictors of Human Chronic Pain Conditions. *Neuroscience* (2016) 338:36-62. Epub 2016/10/27. doi: 10.1016/j.neuroscience.2016.04.041.

37. Wistrom E, Chase R, Smith PR, Campbell ZT. A Compendium of Validated Pain Genes. *WIREs Mech Dis* (2022) 14(6):e1570. Epub 2022/06/28. doi: 10.1002/wsbm.1570.

38. Sexton JE, Cox JJ, Zhao J, Wood JN. The Genetics of Pain: Implications for Therapeutics. *Annu Rev Pharmacol Toxicol* (2018) 58:123-42. Epub 2017/10/03. doi: 10.1146/annurev-pharmtox-010617-052554.

39. Gu. Z. Complexheatmap: Making Complex Heatmaps. R Package Version 1.6.0. [*https://githubcom/jokergoo/ComplexHeatmap*](https://githubcom/jokergoo/ComplexHeatmap) (2015).

40. Heberle H, Meirelles GV, da Silva FR, Telles GP, Minghim R. Interactivenn: A Web-Based Tool for the Analysis of Sets through Venn Diagrams. *BMC Bioinformatics* (2015) 16(1):169. Epub 2015/05/23. doi: 10.1186/s12859-015-0611-3.

41. Lo JO, D'Mello RJ, Watch L, Schust DJ, Murphy SK. An Epigenetic Synopsis of Parental Substance Use. *Epigenomics* (2023) 15(7):453-73. Epub 2023/06/07. doi: 10.2217/epi-2023-0064.

42. Lessans S, Dorsey SG. The Role for Epigenetic Modifications in Pain and Analgesia Response. *Nurs Res Pract* (2013) 2013:961493. Epub 2013/11/15. doi: 10.1155/2013/961493.

43. Huang EJ, Reichardt LF. Neurotrophins: Roles in Neuronal Development and Function. *Annu Rev Neurosci* (2001) 24:677-736. Epub 2001/08/25. doi: 10.1146/annurev.neuro.24.1.677.

44. Paredes S, Cantillo S, Candido KD, Knezevic NN. An Association of Serotonin with Pain Disorders and Its Modulation by Estrogens. *Int J Mol Sci* (2019) 20(22). Epub 2019/11/17. doi: 10.3390/ijms20225729.

45. Lee EJ, Tournier C. The Requirement of Uncoordinated 51-Like Kinase 1 (Ulk1) and Ulk2 in the Regulation of Autophagy. *Autophagy* (2011) 7(7):689-95. Epub 2011/04/05. doi: 10.4161/auto.7.7.15450.

46. Chen Y, Chen J, Xing Z, Peng C, Li D. Autophagy in Neuroinflammation: A Focus on Epigenetic Regulation. *Aging Dis* (2024) 15(2):739-54. Epub 2023/08/07. doi: 10.14336/AD.2023.0718-1.

47. Lienard C, Pintart A, Bomont P. Neuronal Autophagy: Regulations and Implications in Health and Disease. *Cells* (2024) 13(1). Epub 2024/01/11. doi: 10.3390/cells13010103.

48. Ji RR, Gereau RWt, Malcangio M, Strichartz GR. Map Kinase and Pain. *Brain Res Rev* (2009) 60(1):135-48. Epub 2009/01/20. doi: 10.1016/j.brainresrev.2008.12.011.

49. Funderburk SF, Wang QJ, Yue Z. The Beclin 1-Vps34 Complex--at the Crossroads of Autophagy and Beyond. *Trends Cell Biol* (2010) 20(6):355-62. Epub 2010/04/02. doi: 10.1016/j.tcb.2010.03.002.

50. Smyth P, Sasiwachirangkul J, Williams R, Scott CJ. Cathepsin S (Ctss) Activity in Health and Disease - a Treasure Trove of Untapped Clinical Potential. *Mol Aspects Med* (2022) 88:101106. Epub 2022/07/23. doi: 10.1016/j.mam.2022.101106.

51. Korta A, Kula J, Gomulka K. The Role of Il-23 in the Pathogenesis and Therapy of Inflammatory Bowel Disease. *Int J Mol Sci* (2023) 24(12). Epub 2023/06/28. doi: 10.3390/ijms241210172.

52. Wiley JW, Higgins GA, Athey BD. Stress and Glucocorticoid Receptor Transcriptional Programming in Time and Space: Implications for the Brain-Gut Axis. *Neurogastroenterol Motil* (2016) 28(1):12-25. Epub 2015/12/23. doi: 10.1111/nmo.12706.

53. Schote AB, Jager K, Kroll SL, Vonmoos M, Hulka LM, Preller KH, et al. Glucocorticoid Receptor Gene Variants and Lower Expression of Nr3c1 Are Associated with Cocaine Use. *Addict Biol* (2019) 24(4):730-42. Epub 2018/05/16. doi: 10.1111/adb.12632.

54. Zhang JM, An J. Cytokines, Inflammation, and Pain. *Int Anesthesiol Clin* (2007) 45(2):27-37. Epub 2007/04/12. doi: 10.1097/AIA.0b013e318034194e.

55. Schmelz M, Mantyh P, Malfait AM, Farrar J, Yaksh T, Tive L, et al. Nerve Growth Factor Antibody for the Treatment of Osteoarthritis Pain and Chronic Low-Back Pain: Mechanism of Action in the Context of Efficacy and Safety. *Pain* (2019) 160(10):2210-20. Epub 2019/05/31. doi: 10.1097/j.pain.0000000000001625.

56. Alboni S, Cervia D, Sugama S, Conti B. Interleukin 18 in the Cns. *J Neuroinflammation* (2010) 7:9. Epub 2010/02/02. doi: 10.1186/1742-2094-7-9.

57. Cambier S, Gouwy M, Proost P. The Chemokines Cxcl8 and Cxcl12: Molecular and Functional Properties, Role in Disease and Efforts Towards Pharmacological Intervention. *Cell Mol Immunol* (2023) 20(3):217-51. Epub 2023/02/02. doi: 10.1038/s41423-023-00974-6.

58. Xiong HY, Hendrix J, Schabrun S, Wyns A, Campenhout JV, Nijs J, et al. The Role of the Brain-Derived Neurotrophic Factor in Chronic Pain: Links to Central Sensitization and Neuroinflammation. *Biomolecules* (2024) 14(1). Epub 2024/01/23. doi: 10.3390/biom14010071.

59. Bathina S, Das UN. Brain-Derived Neurotrophic Factor and Its Clinical Implications. *Arch Med Sci* (2015) 11(6):1164-78. Epub 2016/01/21. doi: 10.5114/aoms.2015.56342.

60. Mickle AD, Shepherd AJ, Mohapatra DP. Sensory Trp Channels: The Key Transducers of Nociception and Pain. *Prog Mol Biol Transl Sci* (2015) 131:73-118. Epub 2015/03/07. doi: 10.1016/bs.pmbts.2015.01.002.

61. Xu S, Dong H, Zhao Y, Feng W. Differential Expression of Long Non-Coding Rnas and Their Role in Rodent Neuropathic Pain Models. *J Pain Res* (2021) 14:3935-50. Epub 2022/01/11. doi: 10.2147/JPR.S344339.

62. Ma X, Wang H, Song T, Wang W, Zhang Z. Lncrna Malat1 Contributes to Neuropathic Pain Development through Regulating Mir-129-5p/Hmgb1 Axis in a Rat Model of Chronic Constriction Injury. *Int J Neurosci* (2020) 130(12):1215-24. Epub 2020/02/18. doi: 10.1080/00207454.2020.1731508.

63. Chen Q, Zhang W, Sadana N, Chen X. Estrogen Receptors in Pain Modulation: Cellular Signaling. *Biology of Sex Differences* (2021) 12(1):22. doi: 10.1186/s13293-021-00364-5.

64. Ahmed F, Kamble PG, Hetty S, Fanni G, Vranic M, Sarsenbayeva A, et al. Role of Estrogen and Its Receptors in Adipose Tissue Glucose Metabolism in Pre- and Postmenopausal Women. *J Clin Endocrinol Metab* (2022) 107(5):e1879-e89. Epub 2022/01/28. doi: 10.1210/clinem/dgac042.

65. Seyfried O, Hester J. Opioids and Endocrine Dysfunction. *Br J Pain* (2012) 6(1):17-24. Epub 2012/02/01. doi: 10.1177/2049463712438299.

66. Alonso C, Loevinger BL, Muller D, Coe CL. Menstrual Cycle Influences on Pain and Emotion in Women with Fibromyalgia. *J Psychosom Res* (2004) 57(5):451-8. Epub 2004/12/08. doi: 10.1016/j.jpsychores.2004.05.003.

67. Takao K, Tanda K, Nakamura K, Kasahara J, Nakao K, Katsuki M, et al. Comprehensive Behavioral Analysis of Calcium/Calmodulin-Dependent Protein Kinase Iv Knockout Mice. *PLoS One* (2010) 5(3):e9460. Epub 2010/03/09. doi: 10.1371/journal.pone.0009460.

68. Wolf CL, Pruett C, Lighter D, Jorcyk CL. The Clinical Relevance of Osm in Inflammatory Diseases: A Comprehensive Review. *Front Immunol* (2023) 14:1239732. Epub 2023/10/16. doi: 10.3389/fimmu.2023.1239732.

69. Wu M, Wu S, Chen W, Li Y-P. The Roles and Regulatory Mechanisms of Tgf-Β and Bmp Signaling in Bone and Cartilage Development, Homeostasis and Disease. *Cell Research* (2024) 34(2):101-23. doi: 10.1038/s41422-023-00918-9.

70. Chen CL, Broom DC, Liu Y, de Nooij JC, Li Z, Cen C, et al. Runx1 Determines Nociceptive Sensory Neuron Phenotype and Is Required for Thermal and Neuropathic Pain. *Neuron* (2006) 49(3):365-77. Epub 2006/02/01. doi: 10.1016/j.neuron.2005.10.036.

71. Kosten TR, George TP. The Neurobiology of Opioid Dependence: Implications for Treatment. *Sci Pract Perspect* (2002) 1(1):13-20. Epub 2008/06/24. doi: 10.1151/spp021113.

72. Bunton-Stasyshyn RKA, Wagnon JL, Wengert ER, Barker BS, Faulkner A, Wagley PK, et al. Prominent Role of Forebrain Excitatory Neurons in Scn8a Encephalopathy. *Brain* (2019) 142(2):362-75. Epub 2019/01/03. doi: 10.1093/brain/awy324.

73. Berton O, McClung CA, Dileone RJ, Krishnan V, Renthal W, Russo SJ, et al. Essential Role of Bdnf in the Mesolimbic Dopamine Pathway in Social Defeat Stress. *Science* (2006) 311(5762):864-8. Epub 2006/02/14. doi: 10.1126/science.1120972.

74. Taqi MM, Faisal M, Zaman H. Oprm1 A118g Polymorphisms and Its Role in Opioid Addiction: Implication on Severity and Treatment Approaches. *Pharmgenomics Pers Med* (2019) 12:361-8. Epub 2019/12/11. doi: 10.2147/PGPM.S198654.

75. Koeneke A, Ponce G, Troya-Balseca J, Palomo T, Hoenicka J. Ankyrin Repeat and Kinase Domain Containing 1 Gene, and Addiction Vulnerability. *Int J Mol Sci* (2020) 21(7). Epub 2020/04/09. doi: 10.3390/ijms21072516.

76. Reichmann F, Holzer P. Neuropeptide Y: A Stressful Review. *Neuropeptides* (2016) 55:99-109. Epub 2015/10/07. doi: 10.1016/j.npep.2015.09.008.

77. Mao LM, Wang JQ. Synaptically Localized Mitogen-Activated Protein Kinases: Local Substrates and Regulation. *Mol Neurobiol* (2016) 53(9):6309-15. Epub 2016/11/01. doi: 10.1007/s12035-015-9535-1.

78. Kreek MJ, Nielsen DA, LaForge KS. Genes Associated with Addiction: Alcoholism, Opiate, and Cocaine Addiction. *Neuromolecular Med* (2004) 5(1):85-108. Epub 2004/03/06. doi: 10.1385/NMM:5:1:085.

79. Crews FT, Vetreno RP. Mechanisms of Neuroimmune Gene Induction in Alcoholism. *Psychopharmacology (Berl)* (2016) 233(9):1543-57. Epub 2015/03/20. doi: 10.1007/s00213-015-3906-1.

80. Volkow ND, Morales M. The Brain on Drugs: From Reward to Addiction. *Cell* (2015) 162(4):712-25. Epub 2015/08/16. doi: 10.1016/j.cell.2015.07.046.

81. Themelis K, Gillett JL, Karadag P, Cheatle MD, Giordano NA, Balasubramanian S, et al. Mental Defeat and Suicidality in Chronic Pain: A Prospective Analysis. *J Pain* (2023) 24(11):2079-92. Epub 2023/07/02. doi: 10.1016/j.jpain.2023.06.017.

82. Kim EY, Ahn HS, Lee MY, Yu J, Yeom J, Jeong H, et al. An Exploratory Pilot Study with Plasma Protein Signatures Associated with Response of Patients with Depression to Antidepressant Treatment for 10 Weeks. *Biomedicines* (2020) 8(11). Epub 2020/11/01. doi: 10.3390/biomedicines8110455.

83. Sokolowski M, Wasserman D. Genetic Origins of Suicidality? A Synopsis of Genes in Suicidal Behaviours, with Regard to Evidence Diversity, Disorder Specificity and Neurodevelopmental Brain Transcriptomics. *Eur Neuropsychopharmacol* (2020) 37:1-11. Epub 2020/07/09. doi: 10.1016/j.euroneuro.2020.06.002.

84. Gananca L, Oquendo MA, Tyrka AR, Cisneros-Trujillo S, Mann JJ, Sublette ME. The Role of Cytokines in the Pathophysiology of Suicidal Behavior. *Psychoneuroendocrinology* (2016) 63:296-310. Epub 2015/11/08. doi: 10.1016/j.psyneuen.2015.10.008.

85. Garcia-Quinones JA, Sanchez-Dominguez CN, Serna-Rodriguez MF, Marino-Martinez IA, Rivas-Estilla AM, Perez-Maya AA. Genetic Variants Associated with Suicide Risk in the Mexican Population: A Systematic Literature Review. *Arch Suicide Res* (2024) 28(1):71-89. Epub 2023/02/12. doi: 10.1080/13811118.2023.2176269.

86. Punzi G, Ursini G, Chen Q, Radulescu E, Tao R, Huuki LA, et al. Genetics and Brain Transcriptomics of Completed Suicide. *Am J Psychiatry* (2022) 179(3):226-41. Epub 2022/03/04. doi: 10.1176/appi.ajp.2021.21030299.

87. Coon H, Darlington TM, DiBlasi E, Callor WB, Ferris E, Fraser A, et al. Genome-Wide Significant Regions in 43 Utah High-Risk Families Implicate Multiple Genes Involved in Risk for Completed Suicide. *Mol Psychiatry* (2020) 25(11):3077-90. Epub 2018/10/26. doi: 10.1038/s41380-018-0282-3.

88. Tombacz D, Maroti Z, Kalmar T, Csabai Z, Balazs Z, Takahashi S, et al. High-Coverage Whole-Exome Sequencing Identifies Candidate Genes for Suicide in Victims with Major Depressive Disorder. *Sci Rep* (2017) 7(1):7106. Epub 2017/08/05. doi: 10.1038/s41598-017-06522-3.

89. Sibille KT, Witek-Janusek L, Mathews HL, Fillingim RB. Telomeres and Epigenetics: Potential Relevance to Chronic Pain. *Pain* (2012) 153(9):1789-93. Epub 2012/07/10. doi: 10.1016/j.pain.2012.06.003.

90. Hassett AL, Epel E, Clauw DJ, Harris RE, Harte SE, Kairys A, et al. Pain Is Associated with Short Leukocyte Telomere Length in Women with Fibromyalgia. *J Pain* (2012) 13(10):959-69. Epub 2012/10/04. doi: 10.1016/j.jpain.2012.07.003.

91. Apkarian VA, Hashmi JA, Baliki MN. Pain and the Brain: Specificity and Plasticity of the Brain in Clinical Chronic Pain. *Pain* (2011) 152(3 Suppl):S49-S64. Epub 2010/12/15. doi: 10.1016/j.pain.2010.11.010.

92. Schreiber LR, Grant JE, Odlaug BL. Emotion Regulation and Impulsivity in Young Adults. *J Psychiatr Res* (2012) 46(5):651-8. Epub 2012/03/06. doi: 10.1016/j.jpsychires.2012.02.005.

93. Pasyk S, Sanger N, Kapczinski F, Samaan Z. Evaluation of Bdnf as a Biomarker for Impulsivity in a Psychiatric Population. *Diagnostics (Basel)* (2020) 10(6). Epub 2020/06/25. doi: 10.3390/diagnostics10060419.

94. Mei L, Xiong W-C. Neuregulin 1 in Neural Development, Synaptic Plasticity and Schizophrenia. *Nature Reviews Neuroscience* (2008) 9(6):437-52. doi: 10.1038/nrn2392.

95. Stoltenberg SF, Lehmann MK, Christ CC, Hersrud SL, Davies GE. Associations among Types of Impulsivity, Substance Use Problems and Neurexin-3 Polymorphisms. *Drug Alcohol Depend* (2011) 119(3):e31-8. Epub 2011/06/17. doi: 10.1016/j.drugalcdep.2011.05.025.

96. Khadka S, Narayanan B, Meda SA, Gelernter J, Han S, Sawyer B, et al. Genetic Association of Impulsivity in Young Adults: A Multivariate Study. *Transl Psychiatry* (2014) 4(9):e451. Epub 2014/10/01. doi: 10.1038/tp.2014.95.

97. Shiraishi S, Kobayashi H, Nihashi T, Kato K, Iwano S, Nishino M, et al. Cerebral Glucose Metabolism Change in Patients with Complex Regional Pain Syndrome: A Pet Study. *Radiat Med* (2006) 24(5):335-44. Epub 2006/09/09. doi: 10.1007/s11604-006-0035-0.

98. Morley GK, Mooradian AD, Levine AS, Morley JE. Mechanism of Pain in Diabetic Peripheral Neuropathy. Effect of Glucose on Pain Perception in Humans. *Am J Med* (1984) 77(1):79-82. Epub 1984/07/01. doi: 10.1016/0002-9343(84)90439-x.

99. Greisen J, Juhl CB, Grofte T, Vilstrup H, Jensen TS, Schmitz O. Acute Pain Induces Insulin Resistance in Humans. *Anesthesiology* (2001) 95(3):578-84. Epub 2001/09/29. doi: 10.1097/00000542-200109000-00007.

100. Newton R. Anti-Inflammatory Glucocorticoids: Changing Concepts. *Eur J Pharmacol* (2014) 724:231-6. Epub 2013/06/12. doi: 10.1016/j.ejphar.2013.05.035.

101. Heyes S, Pratt WS, Rees E, Dahimene S, Ferron L, Owen MJ, et al. Genetic Disruption of Voltage-Gated Calcium Channels in Psychiatric and Neurological Disorders. *Prog Neurobiol* (2015) 134:36-54. Epub 2015/09/20. doi: 10.1016/j.pneurobio.2015.09.002.

102. Sims-Lucas S, Goetzman ES, Kleyman TR. Cystic Fibrosis-Related Metabolic Defects: Crosstalk between Ion Channels and Organs. *J Clin Invest* (2024) 134(13). Epub 2024/07/01. doi: 10.1172/JCI182329.

103. Lomberk G, Grzenda A, Mathison A, Escande C, Zhang JS, Calvo E, et al. Kruppel-Like Factor 11 Regulates the Expression of Metabolic Genes Via an Evolutionarily Conserved Protein Interaction Domain Functionally Disrupted in Maturity Onset Diabetes of the Young. *J Biol Chem* (2013) 288(24):17745-58. Epub 2013/04/17. doi: 10.1074/jbc.M112.434670.

104. Wang D, He X, Zheng C, Wang C, Peng P, Gao C, et al. Endoplasmic Reticulum Stress: An Emerging Therapeutic Target for Intervertebral Disc Degeneration. *Front Cell Dev Biol* (2021) 9:819139. Epub 2022/02/19. doi: 10.3389/fcell.2021.819139.

105. Zhang Y, Wang T, Wu S, Tang L, Wang J, Yang J, et al. Notch Signaling Pathway: A New Target for Neuropathic Pain Therapy. *J Headache Pain* (2023) 24(1):87. Epub 2023/07/16. doi: 10.1186/s10194-023-01616-y.

106. Qi C, Guo B, Ren K, Yao H, Wang M, Sun T, et al. Chronic Inflammatory Pain Decreases the Glutamate Vesicles in Presynaptic Terminals of the Nucleus Accumbens. *Mol Pain* (2018) 14:1744806918781259. Epub 2018/05/18. doi: 10.1177/1744806918781259.

107. Saltiel AR, Olefsky JM. Inflammatory Mechanisms Linking Obesity and Metabolic Disease. *J Clin Invest* (2017) 127(1):1-4. Epub 2017/01/04. doi: 10.1172/JCI92035.

108. Okifuji A, Hare BD. The Association between Chronic Pain and Obesity. *J Pain Res* (2015) 8:399-408. Epub 2015/07/24. doi: 10.2147/JPR.S55598.

109. Vourdoumpa A, Paltoglou G, Charmandari E. The Genetic Basis of Childhood Obesity: A Systematic Review. *Nutrients* (2023) 15(6). Epub 2023/03/30. doi: 10.3390/nu15061416.

110. McIntosh I, Dreyer SD, Clough MV, Dunston JA, Eyaid W, Roig CM, et al. Mutation Analysis of Lmx1b Gene in Nail-Patella Syndrome Patients. *Am J Hum Genet* (1998) 63(6):1651-8. Epub 1998/12/05. doi: 10.1086/302165.

111. Yen E, Maron JL. Aberrant Feeding and Growth in Neonates with Prenatal Opioid Exposure: Evidence of Neuromodulation and Behavioral Changes. *Front Pediatr* (2021) 9:805763. Epub 2022/02/08. doi: 10.3389/fped.2021.805763.

112. Heard-Costa NL, Zillikens MC, Monda KL, Johansson A, Harris TB, Fu M, et al. Nrxn3 Is a Novel Locus for Waist Circumference: A Genome-Wide Association Study from the Charge Consortium. *PLoS Genet* (2009) 5(6):e1000539. Epub 2009/06/27. doi: 10.1371/journal.pgen.1000539.

113. Wohr M, Fong WM, Janas JA, Mall M, Thome C, Vangipuram M, et al. Myt1l Haploinsufficiency Leads to Obesity and Multifaceted Behavioral Alterations in Mice. *Mol Autism* (2022) 13(1):19. Epub 2022/05/11. doi: 10.1186/s13229-022-00497-3.

114. Daguet I, Raverot V, Bouhassira D, Gronfier C. Circadian Rhythmicity of Pain Sensitivity in Humans. *Brain* (2022) 145(9):3225-35. Epub 2022/09/15. doi: 10.1093/brain/awac147.

115. Hagenauer MH, Crodelle JA, Piltz SH, Toporikova N, Ferguson P, Booth V, editors. The Modulation of Pain by Circadian and Sleep-Dependent Processes: A Review of the Experimental Evidence. 2017; Cham: Springer International Publishing.

116. Bumgarner JR, McCray EW, Nelson RJ. The Disruptive Relationship among Circadian Rhythms, Pain, and Opioids. *Front Neurosci* (2023) 17:1109480. Epub 2023/03/07. doi: 10.3389/fnins.2023.1109480.

117. He P, Gelissen IC, Ammit AJ. Regulation of Atp Binding Cassette Transporter A1 (Abca1) Expression: Cholesterol-Dependent and - Independent Signaling Pathways with Relevance to Inflammatory Lung Disease. *Respir Res* (2020) 21(1):250. Epub 2020/09/27. doi: 10.1186/s12931-020-01515-9.

118. Wei TC, Lin HY, Lu CC, Chen CM, You LR. Expression of Crip2, a Lim-Domain-Only Protein, in the Mouse Cardiovascular System under Physiological and Pathological Conditions. *Gene Expr Patterns* (2011) 11(7):384-94. Epub 2011/05/24. doi: 10.1016/j.gep.2011.05.001.

119. Radhakrishna U, Kuracha MR, Hamzavi I, Saiyed N, Prajapati J, Rawal RM, et al. Impaired Molecular Mechanisms Contributing to Chronic Pain in Patients with Hidradenitis Suppurativa: Exploring Potential Biomarkers and Therapeutic Targets. *Int J Mol Sci* (2025) 26(3). Epub 2025/02/13. doi: 10.3390/ijms26031039.

120. Harding EK, Zamponi GW. Central and Peripheral Contributions of T-Type Calcium Channels in Pain. *Molecular Brain* (2022) 15(1):39. doi: 10.1186/s13041-022-00923-w.

121. Wang JJ, Li Y. Kcnq Potassium Channels in Sensory System and Neural Circuits. *Acta Pharmacol Sin* (2016) 37(1):25-33. Epub 2015/12/22. doi: 10.1038/aps.2015.131.

122. O'Brien JE, Meisler MH. Sodium Channel Scn8a (Nav1.6): Properties and De Novo Mutations in Epileptic Encephalopathy and Intellectual Disability. *Front Genet* (2013) 4:213. Epub 2013/11/07. doi: 10.3389/fgene.2013.00213.

123. Bjorklund G, Shanaida M, Lysiuk R, Antonyak H, Klishch I, Shanaida V, et al. Selenium: An Antioxidant with a Critical Role in Anti-Aging. *Molecules* (2022) 27(19). Epub 2022/10/15. doi: 10.3390/molecules27196613.

124. Dwivedi T, Zhang H. Lithium-Induced Neuroprotection Is Associated with Epigenetic Modification of Specific Bdnf Gene Promoter and Altered Expression of Apoptotic-Regulatory Proteins. *Front Neurosci* (2014) 8:457. Epub 2015/02/03. doi: 10.3389/fnins.2014.00457.

125. Isaksen TJ, Lykke-Hartmann K. Insights into the Pathology of the Alpha2-Na(+)/K(+)-Atpase in Neurological Disorders; Lessons from Animal Models. *Front Physiol* (2016) 7:161. Epub 2016/05/21. doi: 10.3389/fphys.2016.00161.

126. Wang J, Xiao Q, Wang L, Wang Y, Wang D, Ding H. Role of Abca1 in Cardiovascular Disease. *J Pers Med* (2022) 12(6). Epub 2022/06/25. doi: 10.3390/jpm12061010.

127. Tong Q, Qin W, Li ZH, Liu C, Wang ZC, Chu Y, et al. Slc12a5 Promotes Hepatocellular Carcinoma Growth and Ferroptosis Resistance by Inducing Er Stress and Cystine Transport Changes. *Cancer Med* (2023) 12(7):8526-41. Epub 2023/01/17. doi: 10.1002/cam4.5605.

128. Briottet M, Shum M, Urbach V. The Role of Specialized Pro-Resolving Mediators in Cystic Fibrosis Airways Disease. *Front Pharmacol* (2020) 11:1290. Epub 2020/09/29. doi: 10.3389/fphar.2020.01290.

129. Skyba DA, Radhakrishnan R, Bement MKH, Sluka KA. The Camp Pathway and Pain: Potential Targets for Drug Development. *Drug Discovery Today: Disease Models* (2004) 1(2):115-9. doi: <https://doi.org/10.1016/j.ddmod.2004.07.003>.

130. Yamagishi S, Nakamura K, Imaizumi T. Advanced Glycation End Products (Ages) and Diabetic Vascular Complications. *Curr Diabetes Rev* (2005) 1(1):93-106. Epub 2008/01/29. doi: 10.2174/1573399052952631.

131. Ramasamy R, Yan SF, Schmidt AM. Receptor for Age (Rage): Signaling Mechanisms in the Pathogenesis of Diabetes and Its Complications. *Ann N Y Acad Sci* (2011) 1243:88-102. Epub 2012/01/04. doi: 10.1111/j.1749-6632.2011.06320.x.

132. Kierdorf K, Fritz G. Rage Regulation and Signaling in Inflammation and Beyond. *J Leukoc Biol* (2013) 94(1):55-68. Epub 2013/04/02. doi: 10.1189/jlb.1012519.

133. Wachman EM, Schiff DM, Silverstein M. Neonatal Abstinence Syndrome: Advances in Diagnosis and Treatment. *JAMA* (2018) 319(13):1362-74. Epub 2018/04/04. doi: 10.1001/jama.2018.2640.

134. Zamponi GW, Striessnig J, Koschak A, Dolphin AC. The Physiology, Pathology, and Pharmacology of Voltage-Gated Calcium Channels and Their Future Therapeutic Potential. *Pharmacological Reviews* (2015) 67(4):821-70. doi: 10.1124/pr.114.009654.

135. Popugaeva E, Pchitskaya E, Bezprozvanny I. Dysregulation of Neuronal Calcium Homeostasis in Alzheimer's Disease – a Therapeutic Opportunity? *Biochemical and Biophysical Research Communications* (2017) 483(4):998-1004. doi: <https://doi.org/10.1016/j.bbrc.2016.09.053>.

136. Liu W, Lv Y, Ren F. Pi3k/Akt Pathway Is Required for Spinal Central Sensitization in Neuropathic Pain. *Cell Mol Neurobiol* (2018) 38(3):747-55. Epub 2017/08/30. doi: 10.1007/s10571-017-0541-x.

137. Carter CS, Porges SW. The Biochemistry of Love: An Oxytocin Hypothesis. *EMBO Rep* (2013) 14(1):12-6. Epub 2012/11/28. doi: 10.1038/embor.2012.191.

138. Rivkees SA. Developing Circadian Rhythmicity in Infants. *Pediatrics* (2003) 112(2):373-81. Epub 2003/08/05. doi: 10.1542/peds.112.2.373.
